# Quinoline‐Derived Two‐Photon‐Sensitive Octupolar Probes

**DOI:** 10.1002/open.201700097

**Published:** 2017-07-20

**Authors:** Petra Dunkel, Morgane Petit, Hamid Dhimane, Mireille Blanchard‐Desce, David Ogden, Peter I. Dalko

**Affiliations:** ^1^ Laboratoire de Chimie et Biochimie Pharmacologiques et Toxicologiques Université Paris Descartes 45, rue des Saints-Pères 75270 Paris Cedex 06 France; ^2^ Université de Bordeaux ISM (CNRS UMR5255) Bâtiment A12, 351, Cours de la Libération 33405 Talence Cedex France; ^3^ Laboratoire de Physiologie Cérébrale Université Paris Descartes 45, rue des Saints-Pères 75270 Paris Cedex 06 France

**Keywords:** caged compounds, nonlinear optics, photolysis, protecting groups, reactive intermediates

## Abstract

A systematic study on quinoline‐derived light sensitive probes, having third‐order rotational symmetry is presented. The electronically linked octupolar structures show considerably improved linear and nonlinear photophysical properties under one‐ and two‐photon irradiation conditions compared to the corresponding monomers. Photolysis of the three acetate derivatives shows strong structure dependency: whereas irradiation of the 6‐ and 7‐aminoquinoline derivatives resulted in fast intramolecular cyclization and only trace amounts of fragmentation products, the 8‐aminoquinoline derivative afforded clean and selective photolysis, with a sequential release of their acetate groups (*δ*_u_^[730]^=0.67 GM).

## 1 Introduction

The combined use of two‐photon (TP)‐sensitive probes (switches, actuators and “caged” compounds) with high‐energy pulsed laser‐light excitation enables higher spatiotemporal resolution at greater depth within biological tissues than conventional one‐photon excitation.^1^, ^2^ This is a significant improvement that enables optical imaging and photoactivation at depth in tissues in vivo or in vitro. This field of interest spans from cell biology to targeted therapeutic applications.^3^ The availability of tunable ultrashort‐pulse lasers at near‐IR wavelengths and progress in our understanding of TP chromophore design^4^ have contributed to a rapid emergence of TP techniques in biological applications. In the context of caged compounds, although many TP chromophores have been developed, none satisfy the stringent conditions of TP activation of high water solubility and low pharmacological interference in the target tissues.1r, 5 We showed recently a simple algorithm to improve probe design rationally by 1) the optimization of the substitution pattern of the dipole; 2) the increase of the conjugation length, and 3) the incorporation of allowed symmetry elements in the chromophore, allowing access to improved nonlinear properties (third‐order rotational symmetry, *C*_3_, opens for allowed TP transitions, whereas third‐order central symmetry, *S*_3_, leads to TP‐forbidden transitions).^6^ Although the combination of these elements is promising, the scope for tunability is narrow and limited by biological requirements. The fact that the probe should ideally be soluble at a concentration of at least 10 mm in physiological solutions sets narrow limits for the extension of the conjugation length.

In this study, we investigated the incorporation of third‐order rotational symmetry that might further improve absorption parameters by resonance, enhancing the corresponding transition dipole moments and the magnitude of TP absorption and uncaging cross sections (*σ*_2_ and *δ*_u_, respectively). Two important parameters that permit cooperativity between branches to increase TP absorption are: 1) the density of the TP‐absorbing units per molecule; 2) their synergic interaction through electronic interactions by conjugation or through space by their close proximity, with alternating donor–acceptor or donor–π‐acceptor elements. In these structures, the symmetric charge transfer, as well as the change in quadrupole moment, appear to be important for molecules with small ground‐state mesomeric quadrupole moments. The symmetric charge transfer, from the ends of a conjugated system to the middle, or vice versa, upon excitation is expected to enhance TP absorption cross‐section values (*σ*_2_). The question of whether these *σ*_2_ values can be translated into optimized *δ*_u_ values, as is the case with fluorescent probes for *δ*_f_, remained to be addressed.

We present here a study on aminoquinoline‐derived octupolar probes having third‐order rotational symmetry. Quinolines have been successfully used for the photochemical liberation of phenols, carboxylates, phosphates and diols with examples of the physiologically relevant signaling molecules serotonin, tyrosine, glutamate or kainate.^7^ Recent structure–activity studies showed significant effects of substituents on TP uncaging and, furthermore, extension of π‐conjugation yielded a 5‐benzoyl‐8‐(dimethylamino)quinoline acetate derivative having an enhanced TP uncaging of 2.0 GM (1 GM=10^−50^ cm^4^ s per photon).6b, 6c Concerning previous symmetry‐related studies of the quinoline platform, in the case of directly linked dimers, modest one‐photon and TP uncaging was detected, resulting presumably more from a substitution by the heteroaryl groups than quadrupolar coupling.6a, 6d A modest TPA response was observed for quadrupolar derivatives having (dimethylamino)quinoline moieties directly linked to a fluorene core.6a Furthermore, these probes showed high two‐photon sensitivity (*σ*_2_ *Q*_u_, typically around 2 GM at 730 nm), whereas they were inert under femtosecond irradiation conditions. Fast and selective photolysis was observed, however, by using picosecond irradiation conditions with a remarkably high TP uncaging cross section (*δ*_u_=2.3 GM at 730 nm). At opposite, no photolysis was observed with analogous derivatives having an ethylene spacer in between the fluorene and (dimethylamino)quinoline end‐groups.6a, 8 Octupolar constructs having triphenylamine cores attached through an ethylene spacer at position C6 or C8 of the quinoline were found to have enhanced TP absorption with low (practically zero) uncaging cross sections^8^ [both the dipolar (monomer) and quadrupolar analogues showed very low uncaging quantum yields]. Remarkably, the octupolar derivatives showed much lower levels of fluorescence than their dipolar counterparts, indicating that nonradiative relaxation processes are enhanced in the branched molecules.

**Figure 1:**
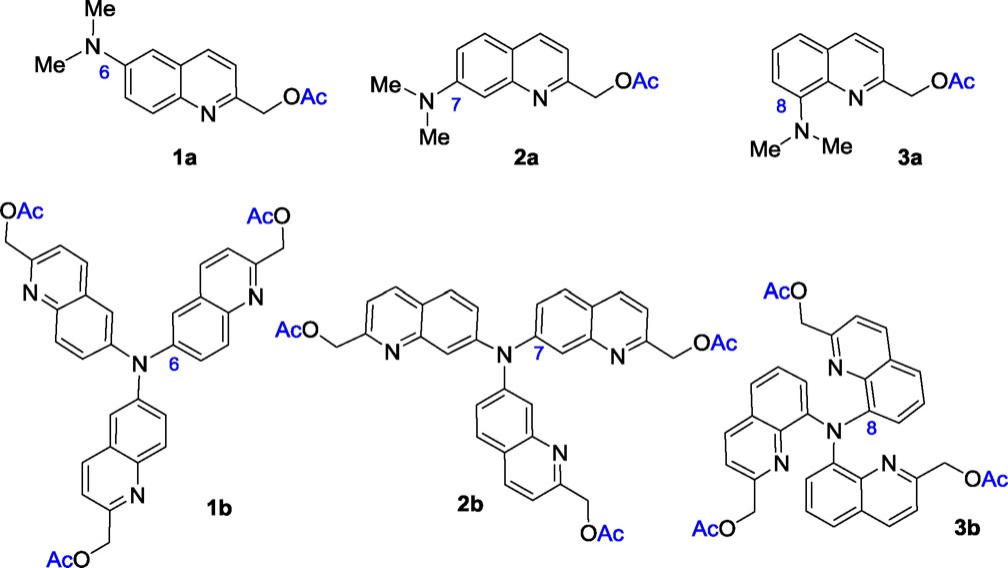
Quinoline‐derived dipolar (**1 a**–**3 a**) and octupolar probes (**1 b**–**3 b**).

As earlier studies indicated a strong influence of the photolysis efficiency on the position of the donor amino group, the synthesis of C6, C7 and C8 isomers **1 b**, **2 b** and **3 b**, respectively, was achieved (Figure 1).

## 2 Results and Discussion

**Scheme 1:**
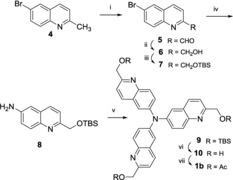
Synthesis of **1 b**. Reagents and conditions: i) SeO_2_ (1.3 equiv), dioxane, 80 °C, 3 h (97 %); ii) NaBH_4_ (1.1 equiv), EtOH, RT, 1 h (85 %); iii) TBSCl (1.1 equiv), imidazole (1.1 equiv), DMF, RT, 3 h (80 %); iv) CuI (0.2 equiv), l‐proline (0.4 equiv), K_2_CO_3_ (3.0 equiv), NH_4_OH (aq. 28 %, 10 equiv), DMSO, 80 °C, 18 h (58 %); v) **7** (2.2 equiv), [Pd_2_(dba)_3_] (0.1 equiv), *t*Bu_3_P (1 m in toluene, 0.2 equiv), *t*BuONa (2.4 equiv), toluene, 110 °C, 18 h (79 %); vi) HF–pyridine (7.5 equiv), MeCN, RT, 1 h (70 %); vii) Ac_2_O (4.5 equiv), Et_3_N (4.5 equiv), DMAP (cat), CH_2_Cl_2_, RT, 2 h (89 %).

Two‐directional Buchwald–Hartwig‐type coupling between haloquinolines and the aminoquinoline appeared to be a straightforward strategy for the preparation of compounds **1 b**–**3 b**. Although the choice of the general strategy was almost trivial, the timing of the functional group transformations (i.e. before or after the coupling) to allow the introduction of the fragmenting side chain was revealed to be a key issue in terms of stability and solubility problems of the intermediates. The optimized reaction sequence is shown in Scheme 1 (for alternative pathways, see the Supporting Information).

Compound **1 b** was prepared from the advanced intermediate **4**.6c The *tert*‐butyldimethylsilyl (TBS)‐protected alcohol **7** was obtained by an oxidation–reduction–protection sequence using standard conditions (67 % overall yield for three steps). Amination of part of the bromoquinoline, **7**, was realized in the presence of ammonia (28 % aq) by using copper(I) iodide/l‐proline catalyst and potassium carbonate, as base.^9^ Aminoquinoline **8** was subjected to bidirectional Buchwald–Hartwig‐type coupling in the presence of 2.2 equivalents of bromoquinaldine with [Pd_2_(dba)_3_]/*t*Bu_3_P as the catalyst and *t*BuONa as a base in toluene at 110 °C (79 % yield). The sequence was completed by deprotection of the alcohol by using HF–pyridine followed by acetylation under standard conditions (62 % overall yield for two steps).

**Scheme 2:**
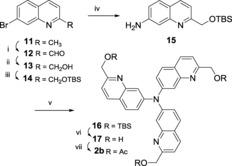
Synthesis of **2 b**. Reagents and conditions: i) SeO_2_ (1.3 equiv), dioxane, 80 °C, 3 h (95 %); ii) NaBH_4_ (1.1 equiv), EtOH, RT, 1 h (78 %); iii) TBSCl (1.2 equiv), imidazole (1.2 equiv), DMF, RT, 3 h (64 %); iv) CuI (0.2 equiv), l‐proline (0.4 equiv), K_2_CO_3_ (3.0 equiv), aq NH_4_OH (28 %, 10 equiv), DMSO, 80 °C, 18 h (35 %); v) **14** (2.2 equiv), [Pd_2_(dba)_3_] (0.2 equiv), *t*BuONa (2.2 equiv), *t*Bu_3_P (1 m in toluene, 0.8 equiv), toluene, 110 °C, 18 h (83 %); vi) HF–pyridine (7.5 equiv), MeCN, RT, 1 h (85 %); vii) Ac_2_O (4.5 equiv), Et_3_N (4.5 equiv), DMAP (cat), CH_2_Cl_2_, RT, 2 h (88 %).

The synthesis of compound **2 b** followed a similar strategy, as shown in Scheme 2. The desired aminoquinoline **15**, obtained from **11** by Cu^I^‐catalyzed amination of the protected alcohol **14** 10 was subjected to a two‐directional Buchwald–Hartwig coupling in the presence of 2.2 equiv of 7‐bromoquinoline **14**, in the presence of [Pd_2_(dba)_3_]/*t*Bu_3_P catalyst and *t*BuONa as a base in toluene (83 % yield). The desired triacetate **2 b** was isolated upon HF–pyridine deprotection and acylation (75 % overall yield for two steps).

**Scheme 3:**
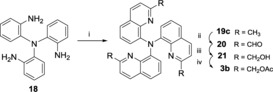
Reagents and conditions: i) crotonaldehyde (9.0 equiv), HCl (6 m), toluene, 120 °C, 3 h (41 %); ii) SeO_2_ (3.3 equiv), dioxane, 80 °C, 3 h (95 %), iii) NaBH_4_ (4.5 equiv), EtOH, RT, 1 h (47 %); iv) Ac_2_O (4.5 equiv), Et_3_N (4.5 equiv), DMAP (cat), CH_2_Cl_2_, RT, 2 h (54 %).

If the analogous Buchwald–Hartwig strategy was attempted in the synthesis of the C8 isomer **3 b**, the reaction formed exclusively the dimer (for further details see the Supporting Information). Therefore, a threefold Doebner–Miller strategy starting from 2,2′,2′′‐triamino‐triphenylamine^11^ was devised (Scheme 3). Under standard conditions (6 m HCl, toluene, 80 °C), in the presence of excess crotonaldehyde, a mixture of singly (**19 a**) and doubly (**19 b**) cyclized products were obtained. The desired triquinaldine **19 c** was obtained at a higher temperature (120 °C) albeit in modest yield (41 %). The usual sequence of oxidation (SeO_2_, 95 %), reduction (NaBH_4_, 47 %) and acetylation (54 %) yielded **3 b**.

**Figure 2:**
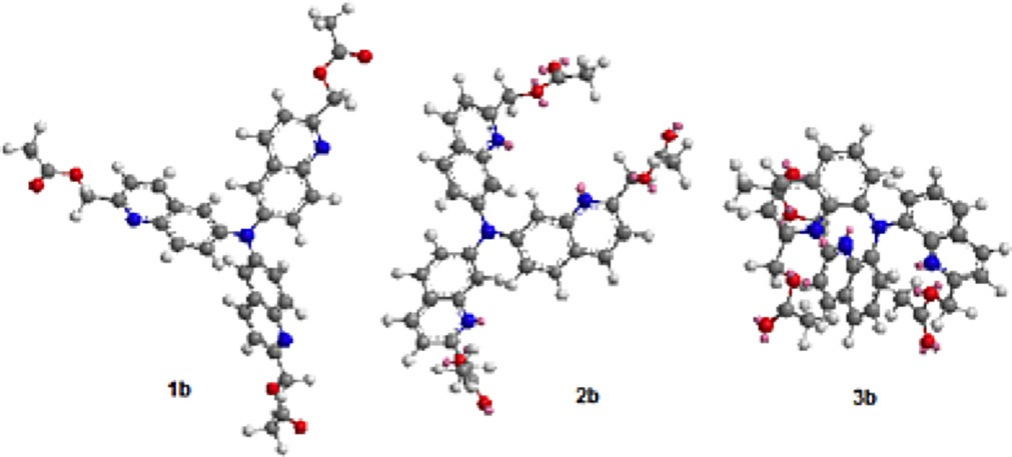
MM2 conformational analysis of compounds **1 b**–**3 b**.

MM2 conformational analysis of **1 b**–**3 b** (Figure 2) showed helix‐like arrangements around the central nitrogen atom with dihedral angles of 40° (**1 b**), 31° (**2 b**), and 34° (**3 b**). Notably, this conformation facilitates partial overlap between the nitrogen doublet and the heteroaromatic rings, resulting in reduced communication between the quinoline monomers. The central nitrogen atom appeared considerably sp^2^‐hybridized—almost planar for **1 b** with bond angles of 119.6°, and somewhat more pyramidal for **2 b** (118.5°) and **3 b** (117.7°). The closest intramolecular distance between the quinoline carbons was 3.2 Å in **1 b**, 3.3 Å in **2 b**, and somewhat larger in **3 b** at 3.7 Å.

The photophysical characteristics of the products are summarized in Table 1. The absorption spectra of **1 b**–**3 b** were recorded in a 1:1 mixture of MeCN/Tris (pH 7.4) at 293 K in the 250–600 nm wavelength range (for details see the Supporting Information). The first absorption maxima were found to be 370, 368 and 380 nm, with molar extinction coefficients (*ϵ* ^max^) of 13 900, 13 900 and 12 900 m ^−1^ cm^−1^, respectively. The absorption maximum of compound **3 b** was slightly redshifted compared to the 8‐(dimethylamino)quinoline monomer, whereas the absorption band in the near‐UV region of all three trimers was characterized by significantly higher molar extinction coefficients versus the monomers (**1 a**: *λ* ^max^=369 nm, *ϵ* ^max^=3400 m ^−1^ cm^−1^; **2 a**: *λ* ^max^=371 nm, *ϵ* ^max^=4800 m ^−1^ cm^−1^; **3 a**: *λ* ^max^=347 nm, *ϵ* ^max^=4800 m ^−1^ cm^−1^). Importantly, trimers **1 b**–**3 b** exhibited modest fluorescence in the visible region (**1 b**: *λ*_em_^max^=489 nm, Stokes shift=6.6×10^3^ cm^−1^, *Φ*_f_=0.20; **2 b**: *λ*_em_^max^=501 nm, Stokes shift=7.2×10^3^ cm^−1^, *Φ*_f_=0.21; **3 b**: *λ*_em_^max^=535 nm, Stokes shift=7.6×10^3^ cm^−1^, *Φ*_f_=0.02). The TP absorption cross sections (*σ*_2_) of **1 b**–**3 b** were measured experimentally in the 700–900 nm range by TP‐induced fluorescence in MeCN or acetone (see Ref. [19] in the Supporting Information). Chromophores **1 b**–**3 b** show TP absorption cross section (*σ*_2_^max^) values of 42 GM, for the tripod **2 b** with an absorption maxima at 705 nm, 22 GM (740 nm) for **3 b**, and 24 GM for **1 b**, with an absorption maxima at 720 nm, which are one order of magnitude larger than for the corresponding monomers (**1 a**: *λ*_TPA_^max^=740 nm, *σ*_2_^max^=3.0 GM *σ*_2_[730]=2.0 GM; **2 a**: *λ*_TPA_^max^=760 nm, *σ*_2_^max^=2.8 GM, *σ*_2_[730]=2.2 GM). In contrast, **3 b** has a comparatively lower TP absorption enhancement (*σ*_2_^max^=22 GM at 740 nm) with respect to the monomer **3 a** (*λ*_TPA_^max^=700 nm, *σ*_2_^max^=10 GM, *σ*_2_[730]=3.0 GM). Differences between the TP absorption cross sections of the tripods might be attributed to the conformational bias that reduces the electronic coupling between the branches.

**Table 1:** Photophysical properties of probes **1 b**–**3 b**.

	*λ* ^max[a]^ [nm]	*ϵ* ^max[a]^ [m ^−1^ cm^−1^]	*ϵ*[366] ^[a, b]^ [m ^−1^ cm^−1^]	*Q*_u_^[b, c]^ [%]	*ϵ*[366]*Q*_u_^[b]^ [m ^−1^ cm^−1^]	*δ*_u_[730] ^[c]^ [GM]	*λ*_TPA_^max^ [nm]	*σ*_2_^max^ ^[d]^ [GM]	*σ*_2_[730] [GM]	*σ*_2_ *Q*_u_ [GM]
**1 b**	370	13 926	13 546	N.A.	N.A.	–	720^[d]^	24^[d]^	22^[d]^	–
**2 b**	368	13 923	13 705	N.A.	N.A.	–	705^[d]^	42^[d]^	28^[d]^	–
**3 b**	380	12 853	11 693	1.5	173	0.67	740^[e]^	22^[e]^	20^[e]^	0.30

[a] Measured in MeCN/Tris buffer (20 mm) 1:1 at 293 K. [b] Measured at 366 nm. [c] Samples (0.1 mm) were prepared in MeCN/Tris buffer (1:1, pH 7.4). [d] Measured in acetone by two‐photon‐induced fluorescence. [e] Measured in acetonitrile. For a full experimental protocol, see the Supporting Information. N.A.=not applicable.

In order to evaluate the photofragmentation of the trimers, aliquot samples of **1 b**–**3 b** (0.1 mm) were irradiated in MeCN/Tris (1:1) at 366 nm. The time course of the UV photolysis was monitored by LC–MS, plotting the consumption of the starting material versus time (see the Supporting Information). No quantitative analysis of the photoreleased acetic acid was made. A rapid photolysis of **1 b** was observed, although instead of liberation of the acid, a carbazole product resulted from intramolecular cyclization. A similar cyclization product was observed from the principal photochemical path for the C7 isomer **2 b**. The photolysis of the C8 isomer **3 b** showed a different reaction course, however, with a sequential liberation of acetate, similar to the previously disclosed fluorene‐derived quadrupolar and triphenylamine‐derived octupolar constructs.6a, 6b, 12 The TP uncaging cross section *δ*_u_ of compound **3 b** was measured experimentally at 730 nm from the conversion of the acetate **3 b** to the free carbinol **21**. A 45 μL sample from a 0.1 mm solution in MeCN/Tris (1:1) was irradiated in a 3 mm path length quartz cuvette by the beam of a Ti–sapphire mode‐locked laser at a wavelength of 730 nm with 150 fs pulses at 80 MHz. The expanded beam was focused with a 32 mm lens so that the whole of the excitation volume was contained in the cuvette. Samples were irradiated for 2–4 h at an average power of 100 mW. The loss of the cage was quantified by HPLC, and the photolysis cross section was calculated from the rate of reduction of the fractional cage concentration at the laser beam parameters given above: compound **3 b** had a TP uncaging cross section of *δ*_u_[730]=0.67 GM, identical to the corresponding monomer (**3 a**: *δ*_u_[730]=0.67 GM). This result is consistent with previous observations on substituted pyridyl radicals,^13^ in which the fragmentation rate was diminished by increased delocalization. The delocalization results lowering of the π* orbital energy, and also in a decrease of σ* character, thus increasing the thermodynamic stability of the overall system. Analogously, the increased thermodynamic stability might result in less productive kinetic excited states for photolysis, as well. The stabilization effect in the present case appears comparable in magnitude to the gain of the sensitivity that results from increased TP absorption.

## 3 Conclusions

The first systematic study on the effect of third‐order rotational symmetry on the one‐photon and two‐photon fragmentation propensity of quinoline‐derived caged compounds was presented. A short synthetic path was developed allowing access to three highly congested structures, **1 b**–**3 b**, having the donor nitrogen as the linchpin between the three branches. Octupolar structures showed enhanced extinction coefficients and TP absorption cross‐section values compared to the corresponding monomers.^7^ Photolysis of **1 b**–**3 b** was strongly dependent on structure: whereas irradiation of **1 b** and **2 b** resulted in fast intramolecular cyclization and only in trace amounts of fragmentation products, compound **3 b** afforded clean and selective photolysis under one‐photon and TP irradiation conditions, with a sequential release of their acetate groups, with photolysis quantum yield and uncaging cross sections close to the parent dipolar derivative **3 a**. This observation is in accordance with the earlier observed trend that probes having higher‐order symmetry behave more like coupled dipoles, providing sequential release of the desired molecule, than a resonant system allowing the “one‐shot” release upon light excitation. A study on alternative octupolar constructs is underway.

## Experimental Section

### Materials and Methods

Thin layer chromatography was performed on aluminum‐backed Merck Kieselgel 60F 254 pre‐coated plates.

^1^H and ^13^C NMR spectra were recorded on a Bruker 250 spectrometer (250 and 63 MHz, respectively) and on a Bruker AV‐500 spectrometer (500 and 125 MHz, respectively). Chemical shifts for protons are reported in parts per million (ppm) downfield from tetramethylsilane and are referenced to residual proton in the NMR solvent (CDCl_3_: *δ*=7.26, [D_6_]acetone: *δ*=2.05, [D_6_]DMSO: *δ*=2.50, [D_4_]MeOH: *δ*=4.78 and 3.31 ppm). Chemical shifts for carbon are reported in ppm downfield from tetramethylsilane and are referenced to the carbon resonances of the solvent (CDCl_3_: *δ*=77.16, [D_6_]acetone: *δ*=29.84, [D_6_]DMSO: *δ*=39.52, [D_4_]MeOH: *δ*=49.15 ppm). Data are represented as follows: chemical shift, multiplicity (s=singlet, d=doublet, dd=double doublet, t=triplet, m=multiplet), coupling constants [Hz], integration. All solvents and inorganic reagents were obtained from commercial sources and used without purification unless otherwise noted.

HPLC analyses were performed on a Waters instrument using a 515 pump with a reverse‐phase X‐Terra MS C18 column (length: 75 mm, diameter: 4.6 mm, stationary phase: 2.5 μm) using a Waters 2487 dual absorbance detector (260–360 nm) and an isocratic system of elution [MeOH/MeCN/H_2_O 7:2:1 or H_2_O/NH_4_OAc (10 mm, pH 4.6)]. The volume of injection was 10 μL. The mass analyzer was an Agilent (model 6100). The capillary tension was 3.5 kV. The cone tension was 24 V. The temperature of the source was 130 °C and the temperature of desolvation was 350 °C. Data were analyzed with ThermoQuest.

#### UV Photolysis

Samples (0.1 mm) were irradiated for 1–2 h in MeCN/Tris buffer mixture (1:1, pH 7.4). An aliquot (1 mL) of this solution was irradiated at approximately 366 nm by using an 8 W Carl Roth lamp. One‐photon quantum yields were determined by using Equation (1):

(1) $$Q{}_{u}=\left[10{}^{3}\;\epsilon {}_{\left(\lambda \mathrm{exc}\right)}\;I{}_{0(\lambda \mathrm{exc})}\;t{}_{90\;\%}\right]{}^{-1}$$

where *ϵ*_(*λ*exc)_ is the molar extinction coefficient of the compound at the excitation wavelength [m ^−1^ cm^−1^]; *t*_90 %_ is the time at which 90 % of the product was converted [s] as determined by HPLC (from the fit of the kinetic data), and *I*_0(*λ*exc)_ is the light intensity at the excitation wavelength [einstein cm^−2^ s^−1^]. Small aliquots (20 μL) of the solution were removed at fixed intervals for analysis by reverse‐phase HPLC using dual absorbance detection at 254 and 360 nm. Dark hydrolysis rates were measured similarly except without illumination.

#### Two‐Photon Photolysis

Near‐IR irradiation experiments were performed by irradiating a solution (0.1 mm in MeCN/Tris buffer, 1:1, pH 7.4) for 1–4 h. TP uncaging cross‐sections (*δ*_u_) were calculated according to the method of Kiskin and Ogden^14^ from the fractional conversion of the cage after exposure for approximately 4 h in a 45 μL cuvette of 3 mm pathlength. Irradiation experiments were performed in a way to minimize the thermal effect: no appreciable temperature change of the samples was observed. Photolysis was achieved in a closed cuvette and the sample was recovered integrally at the end. Two‐photon excitation was calibrated from the fluorescence emission of 1 mM fluorescein (aq) solution in the same apparatus. The expanded output of a MaiTai BB (Spectra‐Physics) pulsed laser was focused with a 30 mm focal length lens into the cuvette. The TP excitation volume was entirely contained within the cuvette volume to obviate the need to measure the beam waist. Beam parameters were 730 nm with 150 fs pulse width at 80 MHz and 100 mW average power after the cuvette. Samples were centrifuged if necessary to remove particles if apparent in the transmitted beam. For reference, the TP uncaging cross section for l‐glutamate release from the widely used 4‐methoxy‐7‐nitroindolinyl‐caged glutamate determined in this way was 0.05 GM. The conversion of the product was assessed by HPLC by monitoring the remaining caged compound. The two‐photon uncaging cross section *δ*_u_ was calculated as follows [Eq. (2)]:

(2) $$\delta_{u}=3.41\;\times \;conversion\;\times \;\frac{V\tau_{P}f}{\pi n\lambda t}{\left(\frac{hc}{P}\right)}^{2}$$

where *δ*_u_ is the TP uncaging cross‐section [cm^4^ s per photon], *V* is the sample volume (45×10^−3^ cm^3^), *τ*_P_ is the pulse width, *f*=80×10^6^ Hz; *n=*1.3, *λ* is the wavelength [cm], *t* is the exposure time [s], *h*=6.6×10^−34^ J s, *c=*3×10^10^ cm s^−1^, and *P* is the average power [W].

### Synthesis of 1 b

#### 1 ‐Bromoquinoline‐2‐carbaldehyde (5)

A mixture of selenium dioxide (1.30 g, 12.0 mmol, 1.3 equiv) in dioxane (50 mL) was heated at 80 °C for 30 min, then quinaldine **4** (2.0 g, 9.0 mmol, 1.0 equiv) was introduced and the mixture was stirred 3 h at 80 °C. After cooling to RT, the mixture was filtered through Celite and eluted with CH_2_Cl_2_ to give **5** as a crude a white solid (2.10 g, 97 %), which was used without further purification for the next step. ^1^H NMR (250 MHz, CDCl_3_): *δ*=9.94 (s, 1 H), 7.96 (d, *J=*8.5 Hz, 1 H), 7.84 (d, *J=*9.0 Hz, 1 H), 7.82–7.75 (m, 2 H), 7.62 ppm (dd, *J=*9.0, 2.0 Hz, 1 H); ^13^C NMR (63 MHz, CDCl_3_): *δ*=193.3, 152.8, 146.5, 136.5, 134.2, 132.1, 131.0, 130.0, 123.7, 118.3 ppm; MS (ESI): *m*/*z*: 236.1, 238.1 [*M*+H]^+^, 268.0. 270.0 (hemiacetal), 282.2, 284.1 (acetal); HRMS (ESI): *m*/*z*: calcd for C_10_H_8_^79^BrNO+H^+^: 235.9771 [*M*+H]^+^; found: 235.9781.

#### (6‐Bromoquinolin‐2‐yl)methanol (6)

NaBH_4_ (28 mg, 0.75 mmol, 1.0 equiv) was added to a mixture of carbaldehyde **5** (177 mg, 0.75 mmol, 1.0 equiv) and EtOH (5 mL) at 0 °C. The mixture was stirred at RT for 30 min before being quenched with HCl (1 m). The EtOH was evaporated and CH_2_Cl_2_ was added to the residue. The organic layer was washed with water (twice) and brine, dried over Na_2_SO_4_, filtered and concentrated under reduced pressure to afford **7** as a white solid (152 mg, 85 %). ^1^H NMR (500 MHz, CDCl_3_): *δ*=8.03 (d, *J=*8.5 Hz, 1 H), 7.97 (d, *J=*2.0 Hz, 1 H), 7.92 (d, *J=*9.0 Hz, 1 H), 7.77 (dd, *J=*9.0, 2.0 Hz, 1 H), 7.31 (d, *J=*8.5 Hz, 1 H), 4.90 (s, 2 H), 4.36 ppm (br s, 1 H); ^13^C NMR (125 MHz, CDCl_3_): *δ*=159.8, 145.5, 135.9, 133.4, 130.5, 129.9, 128.8, 120.3, 119.4, 64.4 ppm; MS (ESI): *m*/*z*: 238.0, 240.0 [*M*+H]^+^; HRMS (ESI): *m*/*z*: calcd for C_10_H_9_BrNO: 237.9868, 239.9847 [*M*+H]^+^; found: 237.9872, 239.9852.

#### 2 ‐Bromo‐2‐{[(*tert*‐butyldimethylsilyl)oxy]methyl}quinoline (7)

A solution of alcohol **6** (2.00 g, 8.00 mmol, 1.0 equiv), TBSCl (1.40 g, 9.00 mmol, 1.1 equiv) and imidazole (623 mg, 9.00 mmol, 1.1 equiv) in DMF (20 mL) was stirred at RT overnight, and then the solvent was removed under reduced pressure. CH_2_Cl_2_ was added and the organic phase was washed with water and brine, dried over Na_2_SO_4_, filtered and concentrated under reduced pressure. The crude product was purified by column chromatography on silica gel (cyclohexane/EtOAc 9:1) to afford the corresponding protected alcohol **7** as a white solid (2.30 g, 80 %). ^1^H NMR (250 MHz, CDCl_3_): *δ*=7.98 (d, *J=*8.5 Hz, 1 H), 7.88–7.78 m, 2 H), 7.67 (d, *J=*8.0 Hz, 2 H), 4.95 (s, 2 H), 0.96 (s, 9 H), 0.13 ppm (s, 6 H); ^13^C NMR (63 MHz, CDCl_3_): *δ*=162.3, 145.8, 135.5, 132.8, 130.4, 129.6, 128.4, 119.6, 119.2, 66.6, 25.9, 18.3, −5.3 ppm; MS (ESI): *m*/*z*: 351.9, 353.9 [*M*+H]^+^; HRMS (ESI): *m*/*z*: calcd for C_16_H_23_BrNOSi: 352.0732, 354.0712 [*M*+H]^+^; found: 352.0743, 354.0726.

#### 3 ‐{[(*tert‐*Butyldimethylsilyl)oxy]methyl}quinolin‐6‐amine (8)

The protected bromoquinoline **7** (600 mg, 1.70 mmol, 1.0 equiv), copper iodide (65 mg, 0.30 mmol, 20 mol %), l‐proline (78 mg, 0.70 mmol, 40 mol %) and K_2_CO_3_ (705 mg, 5.0 mmol, 3.0 equiv) were dissolved in DMSO (20 mL). Aqueous ammonia (28 %, 1.6 mL, 45.4 mmol, 25 equiv) was then introduced and the mixture was heated at 80 °C for 18 h. After cooling to RT, CH_2_Cl_2_ was added followed by saturated NH_4_Cl solution. The aqueous layer was extracted twice with CH_2_Cl_2_, and the combined organic layers were washed again with a saturated NH_4_Cl solution. The product was purified by column chromatography on silica gel (cyclohexane/EtOAc 1:1) to afford **8** as a white solid (284 mg, 58 %). ^1^H NMR (250 MHz, CDCl_3_): *δ*=7.91 (d, *J=*8.5 Hz, 1 H), 7.83 (d, *J=*8.8 Hz, 1 H), 7.57 (d, *J=*8.5 Hz, 1 H), 7.11 (d, *J=*9.0 Hz, 1 H), 6.86 (br s, 1 H), 4.96 (s, 2 H), 3.99 (br s, 2 H), 0.98 (s, 9 H), 0.14 ppm (s, 6 H); ^13^C NMR (63 MHz, CDCl_3_): *δ*=157.9, 144.3, 142.3, 134.5, 129.7, 128.8, 121.6, 118.9, 107.6, 66.8, 26.0, 18.4, −5.2 ppm; MS (ESI): *m*/*z*: 289.2 [*M*+H]^+^; HRMS (ESI): *m*/*z*: calcd for C_16_H_25_N_2_OSi: 289.1736 [*M*+H]^+^; found: 289.1732.

#### Tris(2‐{[(*tert*‐butyldimethylsilyl)oxy]methyl}quinolin‐6‐yl)amine (9)

In a glove box, the amino derivative **8** (100 mg, 0.30 mmol, 1.0 equiv), the bromo derivative **7** (269 mg, 0.80 mmol, 2.2 equiv), [Pd_2_(dba)_3_] (70 mg, 0.07 mmol, 20 mol %) and *t*BuONa (73 mg, 0.80 mmol, 2.2 equiv) were introduced into a sealed tube. Tri‐*tert*‐butylphosphine (1 m in toluene, 64 μL, 0.30 mmol, 80 mol %) and distilled toluene (1.7 mL) were added and the tube was sealed. The mixture was heated at 110 °C for 18 h. After cooling to RT, CH_2_Cl_2_ was added and the organic layer was washed twice with water and brine, dried over Na_2_SO_4_, and concentrated under reduced pressure. The crude product was purified by column chromatography on silica gel (cyclohexane/EtOAc 9:1) to afford **9** as a yellow powder (197 mg, 79 %). ^1^H NMR (250 MHz, CDCl_3_): *δ*=8.18 (s, 1 H), 8.11 (d, *J=*8.5 Hz, 1 H), 7.69 (d, *J=*8.5 Hz, 1 H), 7.63 (d, *J=*8.5 Hz, 1 H), 7.55 (d, *J=*8.5 Hz, 1 H), 4.98 (s, 2 H), 0.97 (s, 9 H), 0.14 ppm (s, 6 H); ^13^C NMR (63 MHz, CDCl_3_): *δ*=160.9, 145.0, 144.7, 135.7, 130.2, 128.4, 128.0, 120.4, 119.1, 66.8, 26.0, 18.5, −5.2 ppm; MS (ESI): *m*/*z*: 831.3 [*M*+H]^+^; HRMS (ESI): *m*/*z*: calcd for C_48_H_67_N_4_O_3_Si_3_: 831.4521 [*M*+H]^+^; found: 831.4560.

#### [Nitrilotris(quinoline‐6,2‐diyl)]trimethanol (10)

HF–pyridine (16 μL, 0.27 mmol, 7.5 equiv) was added to the TBS‐protected alcohol **9** (35 mg, 0.04 mmol, 1.0 equiv) in MeCN (1 mL) at 0 °C, and the mixture was stirred at RT for 2 h in the dark. After completion of the reaction, saturated NaHCO_3_ solution was added and the mixture was concentrated under reduced pressure. A 1:1 mixture of CH_2_Cl_2_ and water (30 mL) was added to the residue and the organic phase was washed with water and brine, dried over Na_2_SO_4_, filtered and concentrated under reduced pressure to afford **10** as a yellow oil (14 mg, 70 %). ^1^H NMR (500 MHz, [D_4_]MeOH): *δ*=8.90 (d, *J=*2.5 Hz, 1 H), 8.46 (d, *J=*6.5 Hz, 1 H), 8.11 (s, 1 H), 8.08 (d, *J=*6.5 Hz, 1 H), 7.99 (d, *J=*3.5 Hz, 1 H), 5.22 ppm (s, 2 H); ^13^C NMR (125 MHz, [D_4_]MeOH): *δ*=161.3, 148.0, 146.8, 136.7, 133.6, 131.1, 123.9, 123.5, 121.7, 62.0 ppm; MS (ESI): *m*/*z*: 489.1 [*M*+H]^+^; HRMS (ESI): *m*/*z*: calcd for C_30_H_25_N_4_O_3_: 489.1927 [*M*+H]^+^; found: 489.1916.

#### [Nitrilotris(quinoline‐6,2‐diyl)]tris(methylene) Triacetate (1 b)

Triol **10** (11 mg, 0.02 mmol, 1.0 equiv), triethylamine (14 μL, 0.10 mmol, 4.5 equiv), acetic anhydride (10 μL, 0.10 mmol, 4.5 equiv) and a catalytic amount of DMAP were dissolved in CH_2_Cl_2_ (100 μL) and the mixture was stirred at RT for 2 h in the dark. The crude product was purified by column chromatography on silica gel (CH_2_Cl_2_/MeOH 95:5) to afford **1 b** as a yellow oil (11 mg, 89 %). ^1^H NMR (500 MHz, CDCl_3_): *δ*=8.10 (d, *J=*8.0 Hz, 1 H), 7.74 (s, 1 H), 7.74 (d, *J=*9.0 Hz, 1 H), 7.46 (dd, *J=*9.0, 2.0 Hz, 1 H), 7.37 (d, *J=*9.0 Hz, 1 H), 5.29 (s, 2 H), 2.16 ppm (s, 3 H); ^13^C NMR (125 MHz, CDCl_3_): *δ*=170.8, 155.3, 145.5, 145.1, 136.1, 130.8, 128.7, 128.3, 120.4, 120.4, 67.4, 27.2 ppm; MS (ESI): *m*/*z*: 615.3 [*M*+H]^+^; HRMS (ESI): *m*/*z*: calcd for C_36_H_31_N_4_O_6_: 615.2244 [*M*+H]^+^; found: 615.2233.

### Synthesis of 2 b

#### 1 ‐{[(*tert‐*Butyldimethylsilyl)oxy]methyl}quinolin‐7‐amine (15)

The protected bromoquinoline **14** (1.00 g, 2.84 mmol, 1.0 equiv), copper iodide (109 mg, 0.57 mmol, 20 mol %), l‐proline (116 mg, 1.14 mmol, 40 mol %) and K_2_CO_3_ (1.2 g, 8.52 mmol, 3.0 equiv) were dissolved in DMSO (4 mL). Aqueous ammonia (28 %) (1 mL, 28.4 mmol, 10 equiv) was then introduced and the mixture was heated at 80 °C for 18 h. After cooling to RT, CH_2_Cl_2_ was added followed by saturated NH_4_Cl solution. The aqueous layer was extracted twice with CH_2_Cl_2_, and the combined organic layers were washed again with a saturated NH_4_Cl solution. The product was purified by column chromatography on silica gel (cyclohexane/EtOAc 3:1) to afford **15** as a white solid (200 mg, 35 %). ^1^H NMR (500 MHz, CDCl_3_): *δ*=7.99 (d, *J=*9.0 Hz, 1 H), 7.57 (d, *J=*8.0 Hz, 1 H), 7.42 (d, *J=*9.0 Hz, 1 H), 7.14 (d, *J=*2.0 Hz, 1 H), 6.92 (dd, *J=*9.0, 2.0 Hz, 1 H), 4.95 (s, 2 H), 4.09 (br s, 2 H), 0.96 (s, 9 H), 0.13 ppm (d, 6 H); ^13^C NMR (63 MHz, CDCl_3_): *δ*=162.1, 149.2, 147.9, 136.3, 128.8, 121.4, 118.1, 115.0, 108.8, 66.9, 26.0, 18.4, −5.2 ppm; MS (ESI): *m*/*z*: 289.2 [*M*+H]^+^; HRMS (ESI): *m*/*z*: calcd for C_16_H_25_N_2_OSi: 289.1736 [*M*+H]^+^; found: 289.1742.

#### Tris(2‐{[(*tert*‐butyldimethylsilyl)oxy]methyl}quinolin‐7‐yl)amine (16)

In a glove box, the amino derivative **15** (100 mg, 0.30 mmol, 1.0 equiv), the bromo derivative **14** (269 mg, 0.80 mmol, 2.2 equiv), [Pd_2_(dba)_3_] (70 mg, 0.07 mmol, 20 mol %) and *t*BuONa (73 mg, 0.80 mmol, 2.2 equiv) were introduced into a sealed tube. Tri‐*tert*‐butylphosphine (1 m in toluene, 64 μL, 0.30 mmol, 80 mol %) and toluene (1.7 mL) were added and the tube was sealed. The mixture was heated at 110 °C for 18 h. After cooling to RT, CH_2_Cl_2_ was added and the organic layer was washed twice with water and brine, dried over Na_2_SO_4_, and concentrated under reduced pressure. The crude product was purified by column chromatography on silica gel (cyclohexane/EtOAc 9:1) to afford **16** as a yellow powder (350 mg, 83 %). ^1^H NMR (250 MHz, CDCl_3_): *δ*=8.06 (d, *J=*8.3 Hz, 1 H), 7.63 (d, *J=*9.3 Hz, 1 H), 7.60 (s, 1 H), 7.55 (d, *J=*8.3 Hz, 1 H), 7.27 (dd, *J=*8.8, 2.3 Hz, 1 H), 4.97 (s, 2 H), 0.98 (s, 9 H), 0.15 ppm (s, 6 H); ^13^C NMR (63 MHz, CDCl_3_): *δ*=162.5, 148.7, 148.4, 136.3, 128.9, 124.6, 124.5, 122.3, 117.5, 66.9, 26.1, 18.5, −5.2 ppm; MS (ESI): *m*/*z*: 831.2 [*M*+H]^+^; HRMS (ESI): *m*/*z*: calcd for C_48_H_67_N_4_O_3_Si_3_: 831.4521 [*M*+H]^+^; found: 831.4517.

#### [7,7′,7′′‐Nitrilotris(quinoline‐7,2‐diyl)]trimethanol (17)

HF–pyridine (16 μL, 0.27 mmol, 7.5 equiv) was added to the TBS‐protected alcohol **16** (30 mg, 0.04 mmol, 1.0 equiv) in MeCN (1 mL) at 0 °C, and the mixture was stirred at RT for 2 h in the dark. After completion of the reaction, saturated NaHCO_3_ solution was added and the mixture was concentrated under reduced pressure. A mixture of CH_2_Cl_2_ and water (1:1, 30 mL) was added to the residue and the organic phase was washed with water and brine, dried over Na_2_SO_4_, filtered and concentrated under reduced pressure. The crude product was purified by column chromatography on silica gel (CH_2_Cl_2_/MeOH 95:5) to afford **17** as a yellow oil (15 mg, 85 %). ^1^H NMR (250 MHz, [D_6_]DMSO): *δ*=7.82 (d, *J=*8.3 Hz, 1 H), 7.45 (d, *J=*8.5 Hz, 1 H), 7.06 (d, *J=*8.5 Hz, 1 H), 7.03 (s, 1 H), 6.93 (d, *J=*8.8 Hz, 1 H), 5.00 (t, *J=*5.5 Hz, 1 H), 4.14 ppm (d, *J=*5.5 Hz, 2 H); ^13^C NMR (125 MHz, [D_6_]DMSO): *δ*=163.1, 147.9, 147.5, 136.1, 129.3, 124.1, 123.9, 121.0, 118.0, 64.7 ppm; MS (ESI): *m*/*z*: 489.2 [*M*+H]^+^; HRMS (ESI): *m*/*z*: calcd for C_30_H_25_N_4_O_3_: 489.1927 [*M*+H]^+^; found: 489.1952.

#### [7,7′,7′′‐Nitrilotris(quinoline‐7,2‐diyl)]tris(methylene) Triacetate (2 b)

Triol **17** (15 mg, 0.03 mmol, 1.0 equiv), triethylamine (19 μL, 0.1 mmol, 4.5 equiv), acetic anhydride (13 μL, 0.1 mmol, 4.5 equiv) and a catalytic amount of DMAP were dissolved in CH_2_Cl_2_ (100 μL) and the mixture was stirred at RT for 2 h in the dark. The crude product was purified by column chromatography on silica gel (CH_2_Cl_2_/MeOH 95:5) to afford **2 b** as a yellow oil (16 mg, 88 %). ^1^H NMR (500 MHz, CDCl_3_): *δ*=8.11 (d, *J=*8.5 Hz, 1 H), 7.75 (d, *J=*8.5 Hz, 1 H), 7.75 (d, *J=*2.5 Hz, 1 H), 7.47 (dd, *J=*8.5, 2.0 Hz, 1 H), 7.38 (d, *J=*8.5 Hz, 1 H), 5.30 (s, 2 H), 2.17 ppm (s, 3 H); ^13^C NMR (125 MHz, CDCl_3_): *δ*=170.8, 156.9, 149.1, 148.4, 136.6, 129.0, 125.2, 124.9, 122.6, 118.7, 67.5, 21.1 ppm; MS (ESI): *m*/*z*: 615.1 [*M*+H]^+^; HRMS (ESI): *m*/*z*: calcd for C_36_H_31_N_4_O_6_: 615.2244 [*M*+H]^+^; found: 615.2221.

### Synthesis of 3 b

*N* ^1^,*N* ^1^‐Bis(2‐aminophenyl)benzene‐1,2‐diamine^11^ (**18**, 822 mg, 2.83 mmol, 1.0 equiv) was dissolved in a solution of HCl (6 m, 20 mL). After addition of crotonaldehyde (1.40 mL, 16.90 mmol, 6.0 equiv) the mixture was stirred for 1 h at RT. Then, toluene (5 mL) was added and the reaction was heated at 80 °C for 3 h. After cooling to RT the organic layer was removed. The aqueous layer was neutralized with NaOH pellets and the solution was extracted with CH_2_Cl_2_. Then the organic layer was washed with water and brine, dried over Na_2_SO_4_, filtered and concentrated under reduced pressure. The crude product was purified by column chromatography on silica gel (cyclohexane/EtOAc 2:1), to afford the single (**19 a**, 140 mg, 14 %) and double (**19 b**, 150 mg, 12 %) cyclization products.

Reacting the bisquinoline product **19 b** (200 mg, 0.51 mmol, 1.0 equiv) in the cyclization with crotonaldehyde (0.13 mL, 1.53 mmol, 3.0 equiv) at 120 °C for 3 h yielded, after work‐up and purification as described above, the threefold cyclization product **19 c** (92 mg, 41 %).

#### *N* ^1^‐(2‐Aminophenyl)‐*N* ^1^‐(2‐methylquinolin‐8‐yl)benzene‐1,2‐diamine (19 a)

Yellow oil. ^1^H NMR (250 MHz, CDCl_3_): *δ*=7.95 (d, *J=*8.3 Hz, 1 H), 7.48 (dd, *J=*8.3, 1.5 Hz, 1 H), 7.33 (t, *J=*7.5 Hz, 1 H), 7.22 (dd, *J=*7.5, 1.5 Hz, 1 H), 7.17 (d, *J=*8.3 Hz, 1 H), 6.97 (td, *J=*7.3, 1.5 Hz, 2 H), 6.90 (dd, *J=*7.8, 1.5 Hz, 2 H), 6.75 (dd, *J=*7.8, 1.5 Hz, 2 H), 6.64 (td, *J=*7.3, 1.5 Hz, 2 H), 3.96 (br s, 4 H), 2.47 ppm (s, 3 H); ^13^C NMR (125 MHz, CDCl_3_): *δ*=157.5, 144.6, 143.1, 142.6, 136.1, 135.1, 128.0, 126.9, 125.7, 125.5, 123.0, 122.8, 122.1, 118.5, 116.3, 25.5 ppm; MS (ESI): *m*/*z*: 341.6 [*M*+H]^+^; HRMS (ESI): *m*/*z*: calcd for C_22_H_21_N_4_: 341.1761 [*M*+H]^+^; found: 341.1748.

#### *N* ^1^,*N* ^1^‐Bis(2‐methylquinolin‐8‐yl)benzene‐1,2‐diamine (19 b)

Yellow oil. ^1^H NMR (250 MHz, CDCl_3_): *δ*=7.92 (d, *J=*8.3 Hz, 2 H), 7.40 (dd, *J=*8.3, 1.3 Hz, 2 H), 7.26 (dd, *J=*8.1, 7.5 Hz, 2 H), 7.13–7.01 (m, 6 H), 6.83 (dd, *J*=8.0, 1.5 Hz, 1 H), 6.67 (td, *J*=7.3, 1.5 Hz, 1 H), 4.55 (br s, 2 H), 2.17 ppm (s, 3 H); ^13^C NMR (63 MHz, CDCl_3_): *δ*=156.0, 146.5, 145.2, 142.8, 135.9, 135.7, 129.3, 127.7, 126.5, 125.7, 122.1, 121.6, 121.3, 118.3, 116.5, 25.1 ppm; MS (ESI): *m*/*z*: 391.7 [*M*+H]^+^; HRMS (ESI): *m*/*z*: calcd for C_26_H_23_N_4_: 391.1917 [*M*+H]^+^; found: 391.1907.

#### Tris(2‐methylquinolin‐8‐yl)amine (19 c)

Yellow oil. ^1^H NMR (250 MHz, CDCl_3_): *δ*=7.94 (d, *J=*8.5 Hz, 3 H), 7.46 (dd, *J=*6.3, 3.0 Hz, 3 H), 7.29–7.21 (m, 6 H), 7.03 (d, *J=*8.5 Hz, 3 H), 2.14 ppm (s, 9 H); ^13^C NMR (63 MHz, CDCl_3_): *δ*=156.2, 147.6, 142.7, 135.7, 127.6, 125.4, 124.8, 121.9, 121.1, 25.1 ppm; MS (ESI): *m*/*z*: 441.4 [*M*+H]^+^; HRMS (ESI): *m*/*z*: calcd for C_30_H_26_N_4_: 441.2074 [*M*+H]^+^; found: 441.2082.

#### 1 ,8′,8′′‐Nitrilotris(quinoline‐2‐carbaldehyde) (20)

A mixture of selenium dioxide (48 mg, 0.43 mmol, 3.3 equiv) and dioxane (2.5 mL) was heated at 80 °C for 30 min. Trisquinaldine **19 c** (57 mg, 0.13 mmol, 1.0 equiv) was then introduced and the mixture was stirred for 3 h at 80 °C. After cooling to RT, the mixture was filtered through Celite, eluted with CH_2_Cl_2_ and the solvents were evaporated under reduced pressure. The product **20** was obtained as a beige solid (60 mg, 95 %) and was used without further purification. ^1^H NMR (250 MHz, CDCl_3_): *δ*=9.03 (s, 1 H), 8.26 (d, *J=*8.5 Hz, 1 H), 7.83 (d, *J=*8.5 Hz, 1 H), 7.62 (dd, *J=*7.8, 1.8 Hz, 1 H), 7.49 (t, *J=*7.8 Hz, 1 H), 7.43 ppm (dd, *J=*7.8, 1.8 Hz, 1 H); ^13^C NMR (125 MHz, CDCl_3_): *δ*=193.6, 150.7, 149.0, 142.9, 137.6, 131.5, 129.6, 125.8, 123.1, 117.0 ppm; MS (ESI): *m*/*z*: 505.3 [*M*+Na]^+^, 537.3 (hemiacetal), 569.4 (hemiacetal), 601.4 (hemiacetal); HRMS (ESI): *m*/*z*: calcd for C_30_H_18_N_4_O_3_Na: 505.1271 [*M*+Na]^+^; found: 505.1293.

#### [8,8′,8′′‐Nitrilotris(quinoline‐8,2‐diyl)]trimethanol (21)

NaBH_4_ (22 mg, 0.59 mmol, 4.5 equiv) was added to trisaldehyde **20** (63 mg, 0.13 mmol, 1.0 equiv) in EtOH (0.5 mL) at 0 °C and the mixture was stirred at RT for 1 h before being quenched with aq HCl (1 m). After removing the EtOH under reduced pressure, water was added to the residue, then the mixture was extracted with CH_2_Cl_2_. The combined organic phases were washed twice with water and brine, dried over Na_2_SO_4_, filtered and evaporated to dryness. The product **21** was obtained as a beige solid (30 mg, 47 %) and was used without further purification. ^1^H NMR (250 MHz, CDCl_3_): *δ*=8.08 (d, *J=*8.5 Hz, 3 H), 7.58 (dd, *J=*5.3, 4.3 Hz, 3 H), 7.43–7.35 (m, 6 H), 7.04 (d, *J=*8.5 Hz, 3 H), 4.42 (s, 6 H), 3.27 ppm (br s, 3 H); ^13^C NMR (63 MHz, CDCl_3_): *δ*=157.3, 147.5, 141.7, 137.2, 129.1, 126.7, 125.5, 123.1, 118.2, 63.4 ppm; MS (ESI): *m*/*z*: 489.3 [*M*+H]^+^; HRMS (ESI): *m*/*z*: calcd for C_30_H_24_N_4_O_3_Na: 511.1741 [*M*+Na]^+^; found: 511.1766.

#### [8,8′,8′′‐Nitrilotris(quinoline‐8,2‐diyl)]tris(methylene) Triacetate (3 b)

Triol **21** (30 mg, 0.06 mmol, 1.0 equiv), triethylamine (38 μL, 0.27 mmol, 4.5 equiv), acetic anhydride (26 μL, 0.27 mmol, 4.5 equiv) and a catalytic amount of DMAP were dissolved in CH_2_Cl_2_ (500 μL) and the mixture was stirred at RT for 2 h in the dark. The crude product was then purified by column chromatography on silica gel (CH_2_Cl_2_/MeOH 95:5) to afford **3 b** as a yellow oil (20 mg, 54 %). ^1^H NMR (250 MHz, CDCl_3_): *δ*=8.10 (d, *J=*8.5 Hz, 3 H), 7.49 (dd, *J=*7.5, 1.5 Hz, 3 H), 7.31 (d, *J=*7.5 Hz, 3 H), 7.27–7.21 (m, 6 H), 4.67 (s, 6 H), 1.97 ppm (s, 9 H); ^13^C NMR (63 MHz, CDCl_3_): *δ*=170.6, 153.7, 147.7, 142.5, 136.8, 128.8, 126.7, 125.2, 122.4, 118.6, 67.6, 20.9 ppm; MS (ESI): *m*/*z*: 615.5 [*M*+H]^+^, 637.5 [*M*+Na]^+^; HRMS (ESI): *m*/*z*: calcd for C_36_H_31_N_4_O_6_: 615.2238 [*M*+H]^+^; found: 615.2224.

## Conflict of interest

*The authors declare no conflict of interest*.

## Supporting information

As a service to our authors and readers, this journal provides supporting information supplied by the authors. Such materials are peer reviewed and may be re‐organized for online delivery, but are not copy‐edited or typeset. Technical support issues arising from supporting information (other than missing files) should be addressed to the authors.

Supplementary
