# Supplementary material for: Quinoline‐Derived Two‐Photon‐Sensitive Octupolar Probes
Source: ChemistryOpen. 2017 Jul 20;6(5):660–7. doi: 10.1002/open.201700097 (PMC5641908; doi:10.1002/open.201700097)
Supplement: Supplementary file 1 — Supplementary [file OPEN-6-660-s001.pdf]

ChemistryOPEN

Supporting Information

© 2017 The Authors. Published by Wiley-VCH Verlag GmbH & Co. KGaA, Weinheim

Quinoline-Derived Two-Photon-Sensitive Octupolar Probes

Petra Dunkel,[a] Morgane Petit,[a] Hamid Dhimane,[a] Mireille Blanchard-Desce,[b] David Ogden,[c] and Peter I. Dalko*[a]

open_201700097_sm_miscellaneous_information.pdf

Supporting Information

Contents

1. UV absorption data of **1b**, **2b** and **3b** S 2

2. Two-photon absorptpion data of **1b**, **2b** and **3b** S 2

3. Comparative UV photolysis of **1b, 2b** and **3b** S 3

4. Alternative synthesis for **1b**, **2b** and **3b** S 4

   a) Alternative synthesis for **1b** S 4
   b) Alternative synthesis for **2b** S 8
   c) The Buchwald-Hartwig coupling strategy in the preparation of **3b** S12

5. ^{1}H and ^{13}C NMR spectra of all novel compounds S 20

1. UV absorption data of **1b**, **2b** and **3bB**

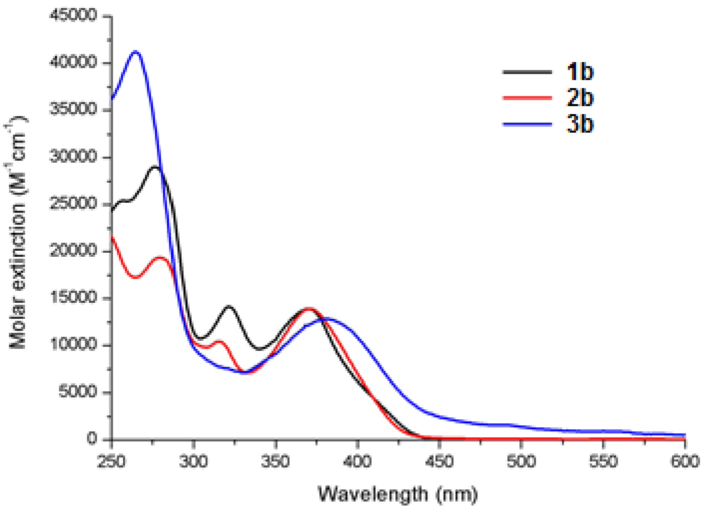

Figure 1. Absorption spectra of compounds **1b**, **2b** and **3b** in TRIS/ACN 1/1 at 293K.

2. Two-photon absortption data of **1b**, **2b** and **3b**.

1b

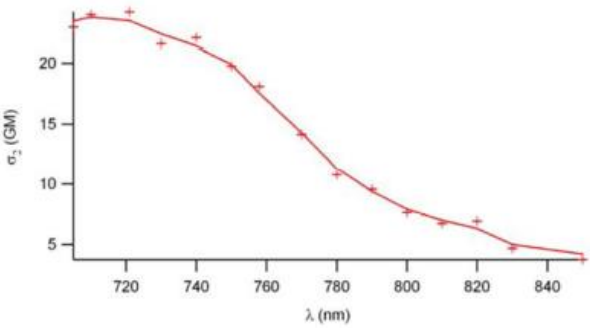

2b

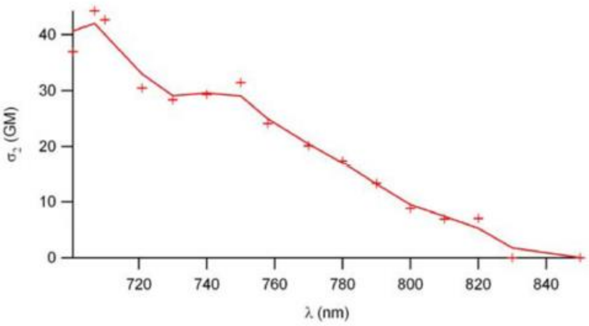

3b

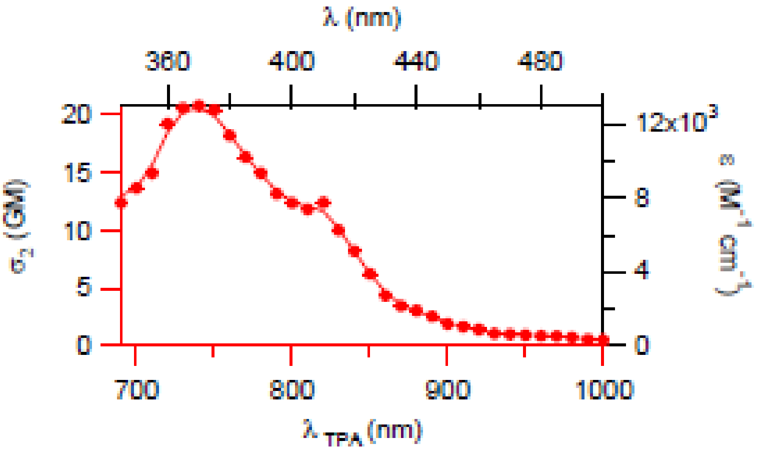

3. Comparative UV photolysis of **1b, 2b** and **3b**.

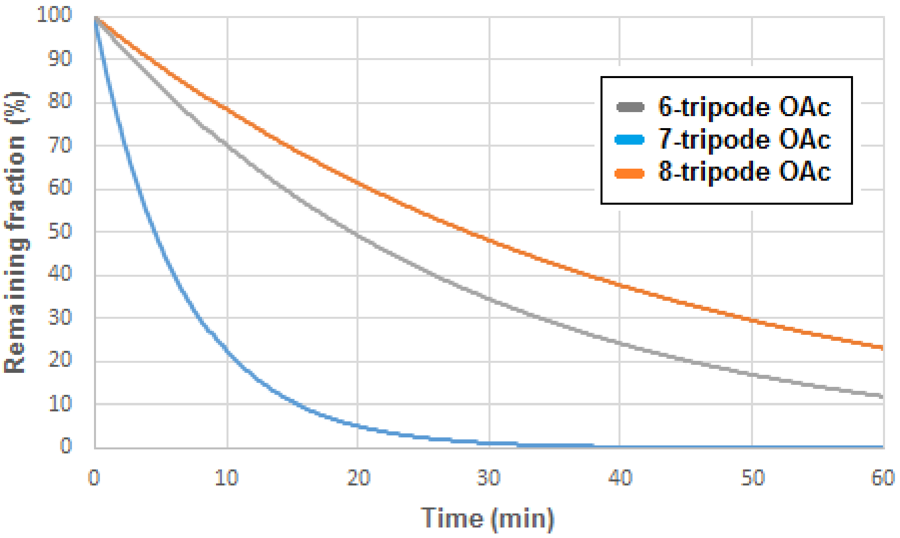

Photolysis of compounds **1b** (6-tripode OAc), **2b** (7-tripode OAc) and **3b** (8-tripode OAc) in ACN/TRIS (20 mM) 1/1 at 293 K at 366 nm with the calculated time course determined by HPLC.

4. Alternative synthesis for **1b**, **2b** and **3b**.

a) Alternative synthesis for **1b.**

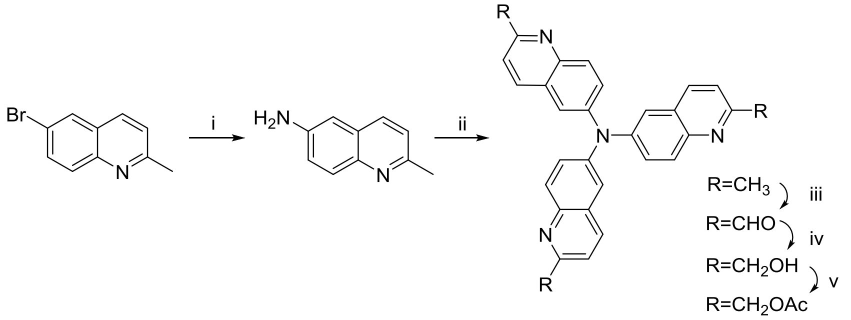

Scheme 1. Reagents and conditions: (i) CuI (0.2 equiv), *L*-proline (0.4 equiv), K_2CO_3 (3.0 equiv), NH_4OH (aq. 28%, 10 equiv), DMSO, 80 °C, 18 h (82%); (ii) 6-bromo-2-methylquinoline (2.2 equiv), Pd_2dba_3 (0.1 equiv), $NaOC(CH_3)_3$ (2.4 equiv), P^tBu_3 (1M in toluene, 0.2 equiv), toluene, 110°C 18 h (65%); (iii) SeO_2 (3.3 equiv), dioxane, 80°C, 3 h (59%); (iv) $NaBH_4$ (3.3 equiv), EtOH, rt, 30 min (50%); v) Ac_2O (4.5 equiv), Et_3N (4.5 equiv), DMAP (cat), DCM, rt, 2 h (89%).

2-Methylquinolin-6-amine

6-Bromo-2-methylquinoline (800 mg, 3.60 mmol, 1.0 equiv), copper iodide (137 mg, 0.72 mmol, 20 mol%), *L*-proline (166 mg, 1.44 mmol, 40 mol%) and K_2CO_3 (1.50 mg, 10.80 mmol, 3.0 equiv) were dissolved in DMSO (10 mL). Aqueous ammonia (28%) (2.48 mL, 36.00 mmol, 10 eq) was then introduced and the mixture was heated at 80°C for 18 h. After cooling to room temperature, dichloromethane was added followed by saturated NH_4Cl solution. The aqueous layer was extracted twice with DCM, and the combined organic layers were washed again with saturated NH_4Cl solution. The product was purified by column chromatography on silica gel (DCM:MeOH 95:5). Beige solid (467 mg, 82%). ^1H NMR (250 MHz, $CDCl_3$) δ 7.82 (d, *J*=9.0 Hz, 1H), 7.77 (d, *J*=8.8 Hz, 1H), 7.14 (d, *J*=8.3 Hz, 1H), 7.10 (dd, *J*=8.0, 2.5 Hz, 1H), 6.85 (d, *J*=2.3 Hz, 1H), 3.87 (brs, 2H), 2.65 (s, 3H). ^{13}C NMR (63 MHz, $CDCl_3$) δ 155.1, 144.1, 142.9, 134.3, 129.7, 127.9, 122.3, 121.5, 107.8, 25.0. MS (ESI): *m/z* = 180.7 $[M+Na]^+$. HRMS (ESI): *m/z* calcd for $[C_{10}H_{10}N_2+H]^+$ 159.0917, found 159.0914.

Tris(2-methylquinolin-6-yl)amine

In a sealed tube placed in a glove box, 2-methylquinolin-6-amine (100 mg, 0.63 mmol, 1.0 equiv), 6-bromo-2-methylquinoline (308 mg, 1.39 mmol, 2.2 equiv), Pd_2dba_3 (65 mg, 0.06 mmol, 10 mol%) and $NaOC(CH_3)_3$ (144 mg, 1.51 mmol, 2.4 equiv) were introduced. 1M solution (in toluene) of tri-*tert*-butylphosphine (27 µL, 0.13 mmol, 20 mol%) and distilled toluene (4 mL) were added and the tube was sealed. The mixture was heated at 110 °C for 18 h. After cooling to room temperature, the solvent was removed under reduced pressure, then DCM was added and the organic layer was washed twice with water and brine, dried over Na_2SO_4, and concentrated under reduced pressure. The product was purified by column chromatography on silica gel (DCM:MeOH 98:1→9:1). Yellow powder (180 mg, 65%). ^1H NMR (250 MHz, $CDCl_3$) δ 7.93 (d, *J*=8.8 Hz, 1H), 7.65 (d, *J*=8.3 Hz, 1H), 7.53 (d, *J*=9.3 Hz, 1H), 7.39 (s, 1H), 7.16 (d, *J*=8.3 Hz, 1H), 2.67 (s, 3H). ^{13}C NMR (63 MHz, $CDCl_3$) δ 157.8, 145.0, 144.7, 135.2, 129.9, 127.7, 127.4, 122.4, 120.1, 25.1. MS (ESI): *m/z* = 441.3 $[M+H]^+$.

6,6',6''-Nitrilotris(quinoline-2-carbaldehyde)

A mixture of selenium dioxide (84 mg, 0.76 mmol, 3.3 equiv) in dioxane (3 mL) was heated at 80°C for 30 min, then tris(2-methylquinolin-6-yl)amine (103 mg, 0.23 mmol, 1.0 equiv) was introduced and the mixture was stirred 3 h at 80°C. After cooling down to room temperature, the mixture was filtered through celite, eluted with DCM. The crude product was purified by column chromatography on silica gel (DCM:MeOH 98:2). Yellow oil (66 mg, 59%). ^1H NMR (500 MHz, acetone-d_6) δ 10.14 (s, 1H), 8.39 (d, *J*=9.0 Hz, 1H), 8.24 (d, *J*=8.5 Hz, 1H), 7.96 (d, *J*=9.0 Hz, 1H), 7.89-7.84 (m, 2H). ^{13}C NMR (125 MHz, acetone-d_6) δ 194.0, 147.9, 146.4, 137.5, 132.9, 132.4, 129.7, 121.9, 118.5.

(Nitrilotris(quinoline-6,2-diyl))trimethanol – via $NaBH_4$ reduction

To a mixture of 6,6',6''-nitrilotris(quinoline-2-carbaldehyde) (58 mg, 0.12 mmol, 1.0 equiv) and EtOH (5 mL), $NaBH_4$ (15 mg, 0.40 mmol, 3.3 equiv) was added at 0 °C. The mixture was stirred at room temperature for 30 min before being quenched with 1M HCl. The EtOH was evaporated, the residue was taken up in DCM and the organic layer was washed with water (2×) and brine, dried over Na_2SO_4, filtered and concentrated under reduced pressure. The crude product was crystallized from MeOH. Yellow crystals (29 mg, 50%).

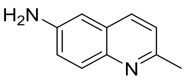
H2N
N

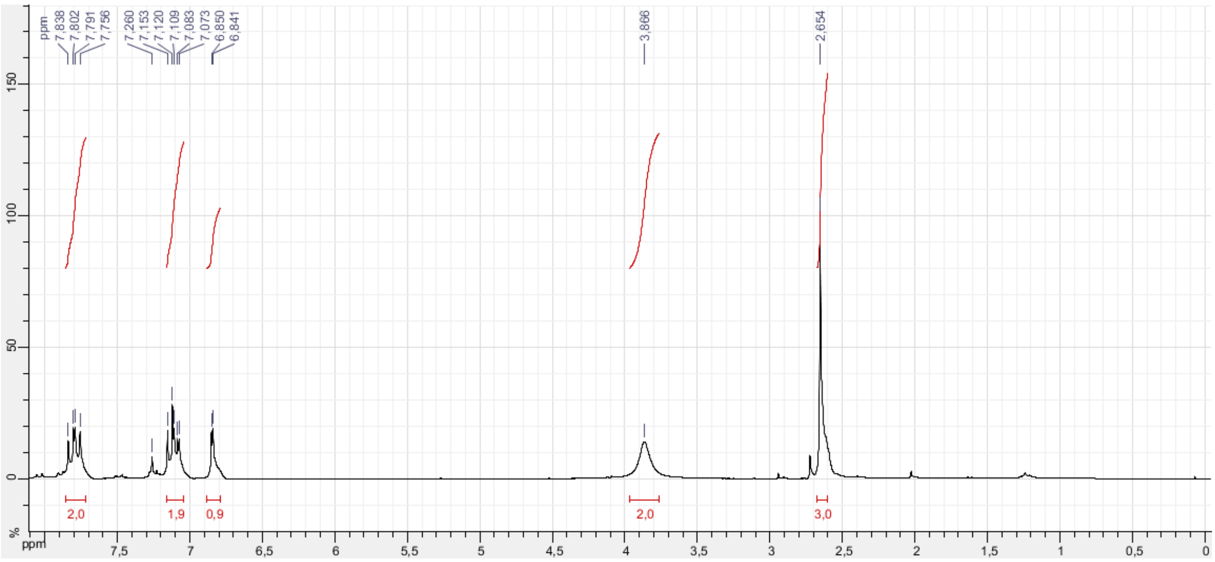
ppm
7,838
7,802
7,791
7,756
7,260
7,153
7,120
7,109
7,083
7,073
6,850
6,841
3,866
2,654
2,0
1,9
0,9
2,0
3,0
150
100
50
0
%
ppm
7,5
7
6,5
6
5,5
5
4,5
4
3,5
3
2,5
2
1,5
1
0,5
0

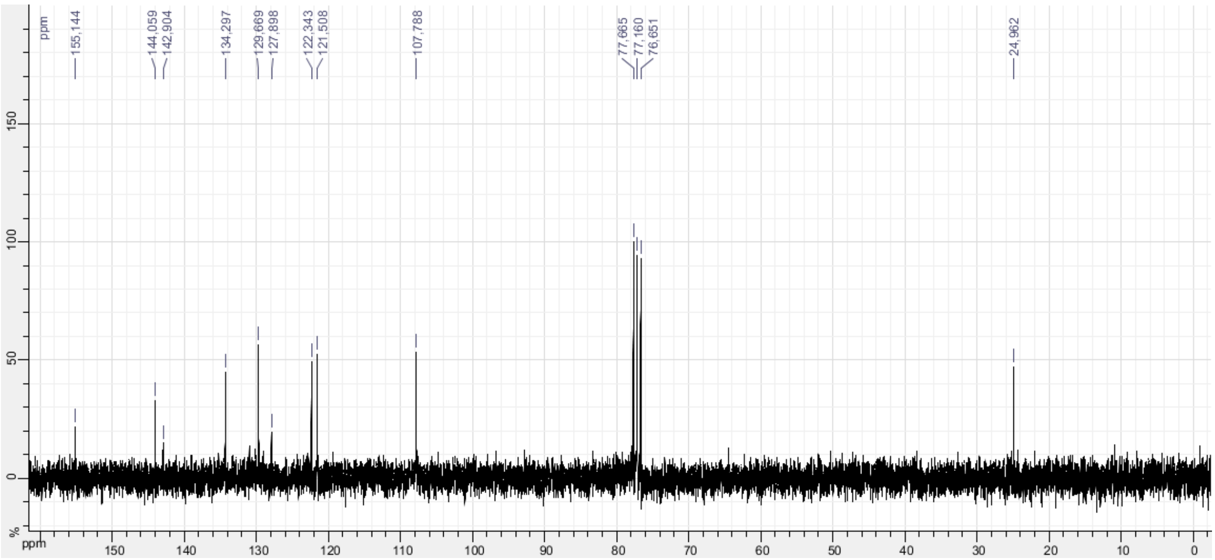
ppm
155,144
144,059
142,904
134,297
129,669
127,898
122,343
121,508
107,788
77,665
77,160
76,651
24,962
150
100
50
0
%
ppm
150
140
130
120
110
100
90
80
70
60
50
40
30
20
10
0

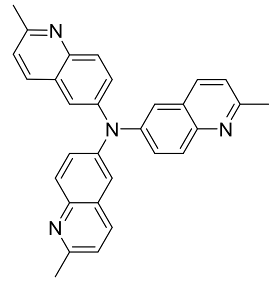
N
N
N
N

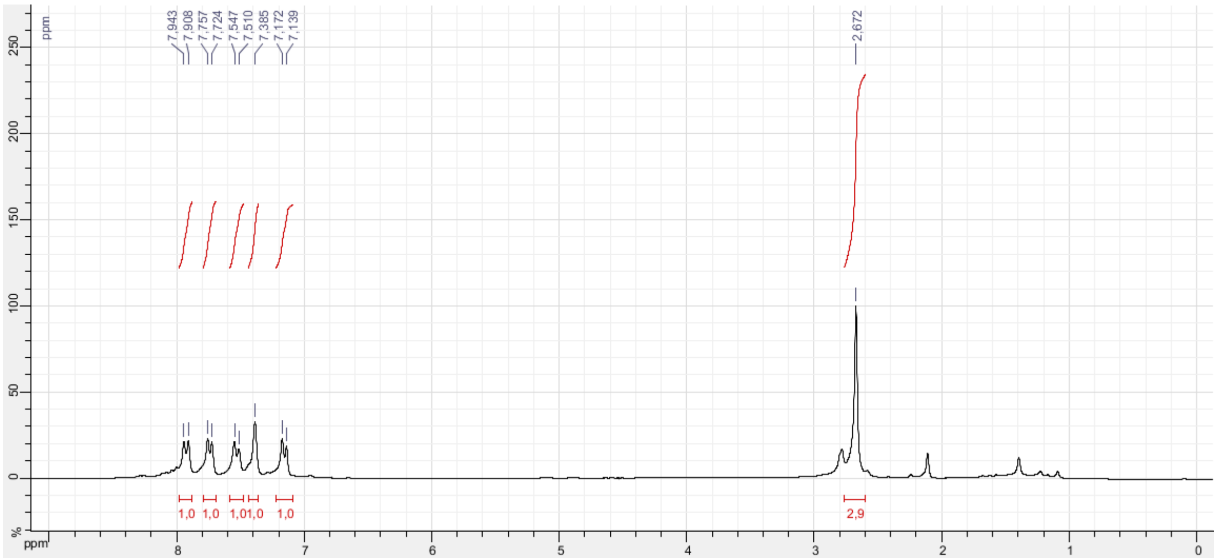
7,943
7,908
7,757
7,724
7,547
7,510
7,385
7,172
7,139
2,672
1,0
1,0
1,01,0
1,0
2,9
ppm
%
250
200
150
100
50
0
8
7
6
5
4
3
2
1
0

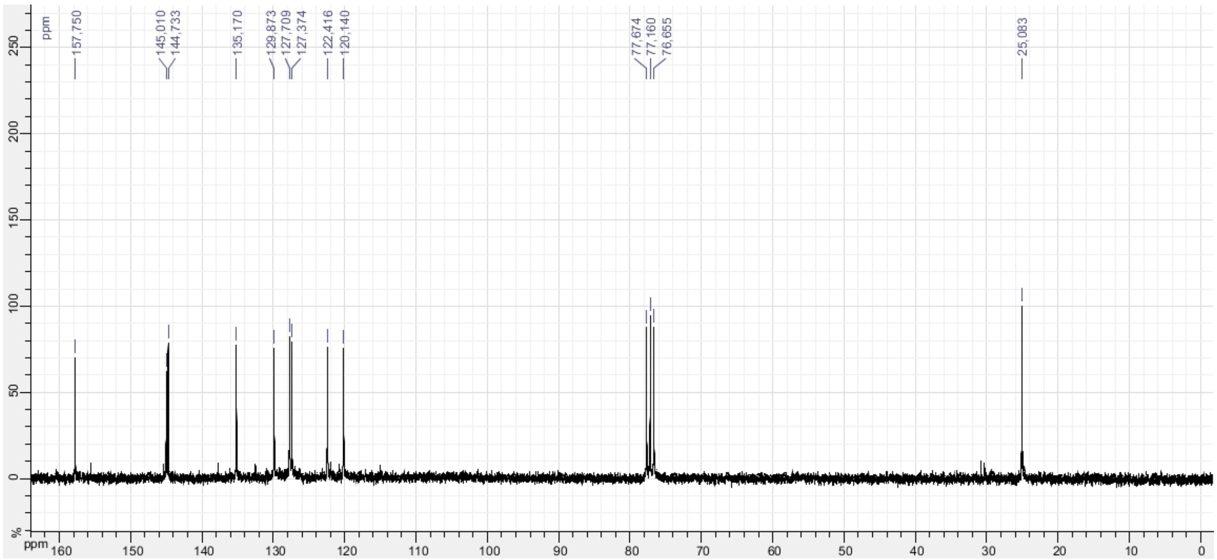
157,750
145,010
144,733
135,170
129,873
127,709
127,374
122,416
120,140
77,674
77,160
76,655
25,083
ppm
%
250
200
150
100
50
0
160
150
140
130
120
110
100
90
80
70
60
50
40
30
20
10
0

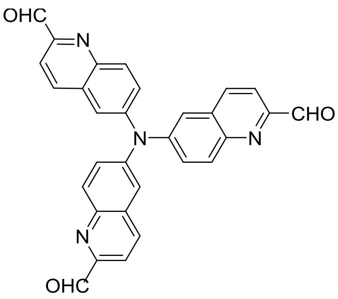
OHC
N
N
CHO
N
N
OHC

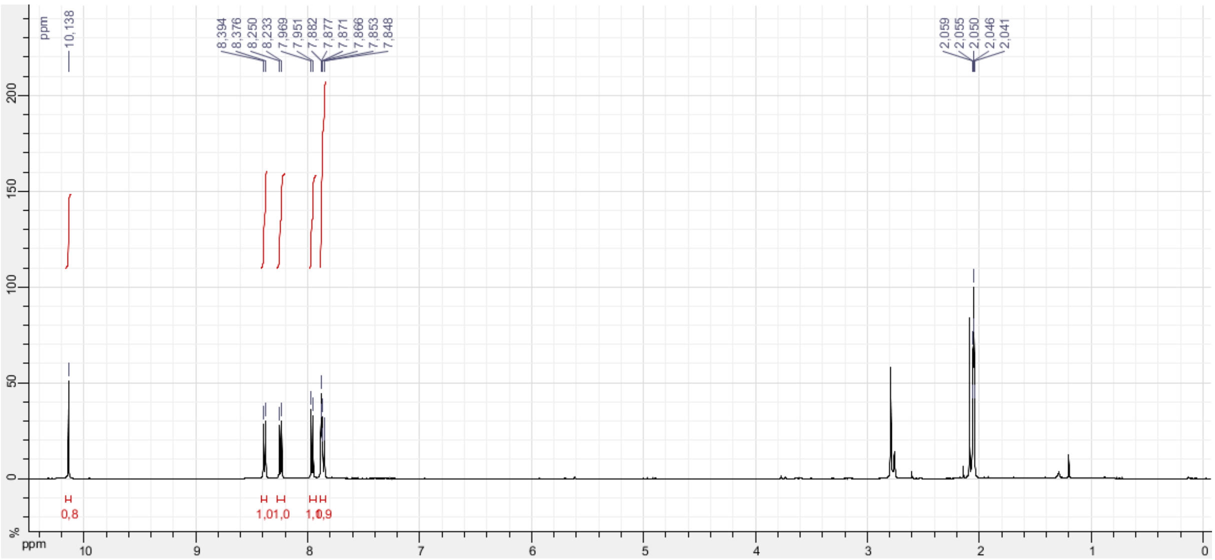
ppm
10,138
8,394
8,376
8,250
8,233
7,969
7,951
7,882
7,877
7,871
7,866
7,853
7,848
2,059
2,055
2,050
2,046
2,041
0,8
1,0
1,0
1,0
0,9
%
ppm

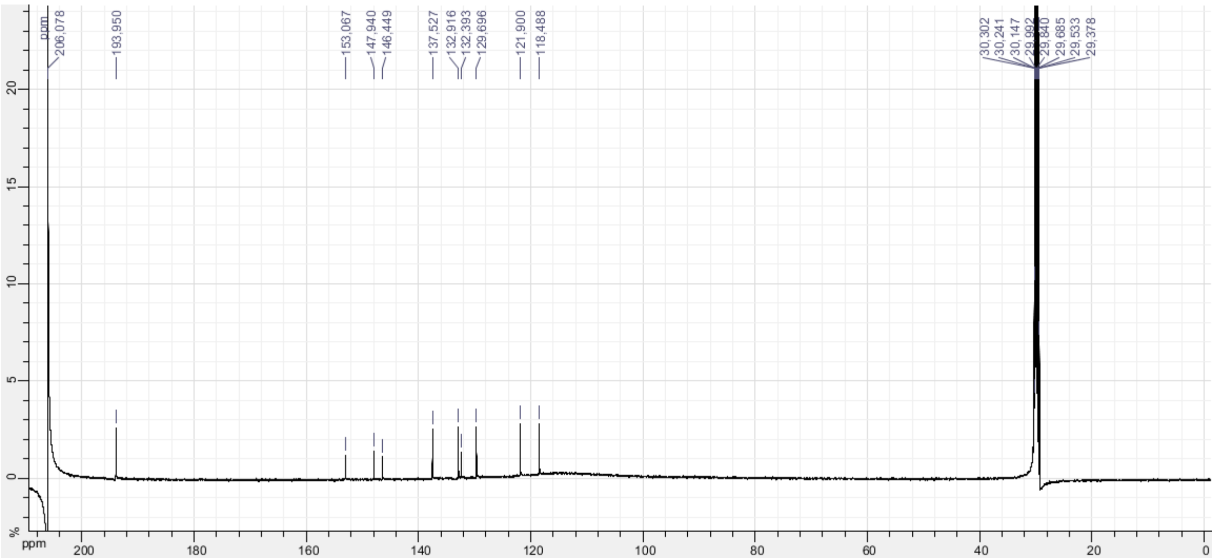
ppm
206,078
193,950
153,067
147,940
146,449
137,527
132,916
132,393
129,696
121,900
118,488
30,302
30,241
30,147
29,992
29,840
29,685
29,533
29,378
%
ppm

b) Alternative synthesis for **2b.**

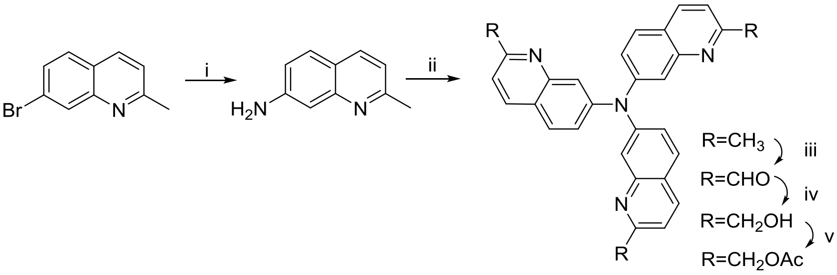

Scheme 2. Reagents and conditions: (i) CuI (0.2 equiv), *L*-proline (0.4 equiv), K_2CO_3 (3.0 equiv), NH_4OH (aq. 28%, 10 equiv), DMSO, 80 °C, 18 h (76%); (ii) 2-methylquinolin-7-amine (2.2 equiv), Pd_2dba_3 (0.1 equiv), $NaOC(CH_3)_3$ (2.4 equiv), P^tBu_3 (1M in toluene, 0.2 equiv), toluene, 110°C 18 h (60%); (iii) SeO_2 (3.3 equiv), dioxane, 80°C, 3 h (70%); (iv) $NaBH_4$ (3.3 equiv), EtOH, rt, 30 min (68%); (v) Ac_2O (4.5 equiv), Et_3N (4.5 equiv), DMAP (cat), DCM, rt, 2 h (88%).

2-Methylquinolin-7-amine

7-Bromo-2-methylquinoline (800 mg, 3.60 mmol, 1.0 equiv), copper iodide (137 mg, 0.72 mmol, 20 mol%), *L*-proline (166 mg, 1.44 mmol, 40 mol%) and K_2CO_3 (1.50 mg, 10.80 mmol, 3.0 equiv) were dissolved in DMSO (10 mL). Aqueous ammonia (28%) (2.48 mL, 36.00 mmol, 10 eq) was then introduced and the mixture was heated at 80°C for 18 h. After cooling to room temperature, dichloromethane was added followed by saturated NH_4Cl solution. The aqueous layer was extracted twice with DCM, and the combined organic layers were washed again with saturated NH_4Cl solution. The product was purified by column chromatography on silica gel (DCM:MeOH 95:5). Beige solid (434 mg, 76%). ^{1}H NMR (250 MHz) δ 7.77 (d, *J* = 8.0 Hz, 1H), 7.46 (d, *J* = 8.5 Hz, 1H), 7.11 (s, 1H), 6.94 (d, *J* = 8.5 Hz, 1H), 6.83 (dd, *J* = 8.5, 1.8 Hz, 1H), 4.10 (br s, 2H, 2.61 (s, 3H). ^{13}C NMR (63 MHz) δ 159.1, 149.6, 147.9, 135.8, 128.6, 120.3, 118.4, 117.6, 108.7, 25.2. MS (ESI): *m/z* = 159.2 $[M+H]^+$. HRMS (ESI): *m/z* calcd for $[C_{10}H_{10}N_2+H]^+$ 159.0917, found 159.0917.

Tris(2-methylquinolin-7-yl)amine

In a sealed tube placed in a glove box, 2-methylquinolin-7-amine (100 mg, 0.63 mmol, 1.0 equiv), 7-bromo-2-methylquinoline (308 mg, 1.39 mmol, 2.2 equiv), Pd_2dba_3 (65 mg, 0.06 mmol, 10 mol%) and $NaOC(CH_3)_3$ (144 mg, 1.51 mmol, 2.4 equiv) were introduced. 1M solution (in toluene) of tri-*tert*-butylphosphine (27 µL, 0.13 mmol, 20 mol%) and distilled toluene (4 mL) were added and the tube was sealed. The mixture was heated at 110 °C for 18 h. After cooling to room temperature, the solvent was removed under reduced pressure, then DCM was added and the organic layer was washed twice with water and brine, dried over Na_2SO_4, and concentrated under reduced pressure. The product was purified by column chromatography on silica gel (DCM:MeOH 98:1→9:1). Yellow powder (167 mg, 60%). ^{1}H NMR (250 MHz) δ 7.91 (d, *J* = 8.0 Hz, 1H), 7.68 (d, *J* = 4.8 Hz, 1H), 7.63 (d, *J* = 9.0 Hz, 1H), 7.38 (dd, *J* = 9.0, 2.3 Hz, 1H), 7.13 (d, *J* = 8.0 Hz, 1H), 2.62 (s, 3H). ^{13}C NMR (63 MHz) δ 159.4, 149.1, 148.3, 135.7, 128.6, 124.1, 123.5, 122.2, 120.9, 25.3. MS (ESI): *m/z* = 441.3 $[M+H]^+$. HRMS (ESI): *m/z* calcd for $[C_{30}H_{24}N_4+H]^+$ 441.2074, found 441.2061.

7,7',7''-Nitrilotris(quinoline-2-carbaldehyde)

A mixture of selenium dioxide (84 mg, 0.76 mmol, 3.3 equiv) in dioxane (3 mL) was heated at 80°C for 30 min, then tris(2-methylquinolin-7-yl)amine (103 mg, 0.23 mmol, 1.0 equiv) was introduced and the mixture was stirred 3 h at 80°C. After cooling down to room temperature, the mixture was filtered through celite, eluted with DCM. The crude product was purified by column chromatography on silica gel (DCM:MeOH 98:2). Yellow oil (78 mg, 70%). ^{1}H NMR (250 MHz) δ 10.06 (s, 1H), 8.22 (d, *J* = 8.3 Hz, 1H), 7.97 (d, *J* = 1.8 Hz, 1H), 7.91 (d, *J* = 8.3 Hz, 1H), 7.85 (d, *J* = 8.8 Hz, 1H), 7.64 (dd, J = 8.8, 1.8 Hz). ^{13}C NMR (63 MHz) δ 193.4, 153.1, 149.2, 148.4, 137.0, 129.5, 127.5, 127.3, 123.3, 116.9. MS (ESI): *m/z* = 481.5 $[M-H]^-$. HRMS (ESI): *m/z* calcd for $[C_{30}H_{18}O_3N_4-H]^-$ 481.1306, found 481.1327.

(7,7',7''-Nitrilotris(quinoline-7,2-diyl))trimethanol – via $NaBH_4$ reduction

To a mixture of 7,7',7''-nitrilotris(quinoline-2-carbaldehyde) (58 mg, 0.12 mmol, 1.0 equiv) and EtOH (5 mL), $NaBH_4$ (15 mg, 0.40 mmol, 3.3 equiv) was added at 0 °C. The mixture was stirred at room temperature for 30 min before being quenched with 1M HCl. The EtOH was evaporated, the residue was taken up in DCM and the organic layer was washed with water (2×) and brine, dried over Na_2SO_4, filtered and concentrated under reduced pressure. The crude product was crystallized from MeOH. Yellow crystals (40 mg, 68%).

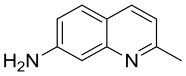
H_2N
N

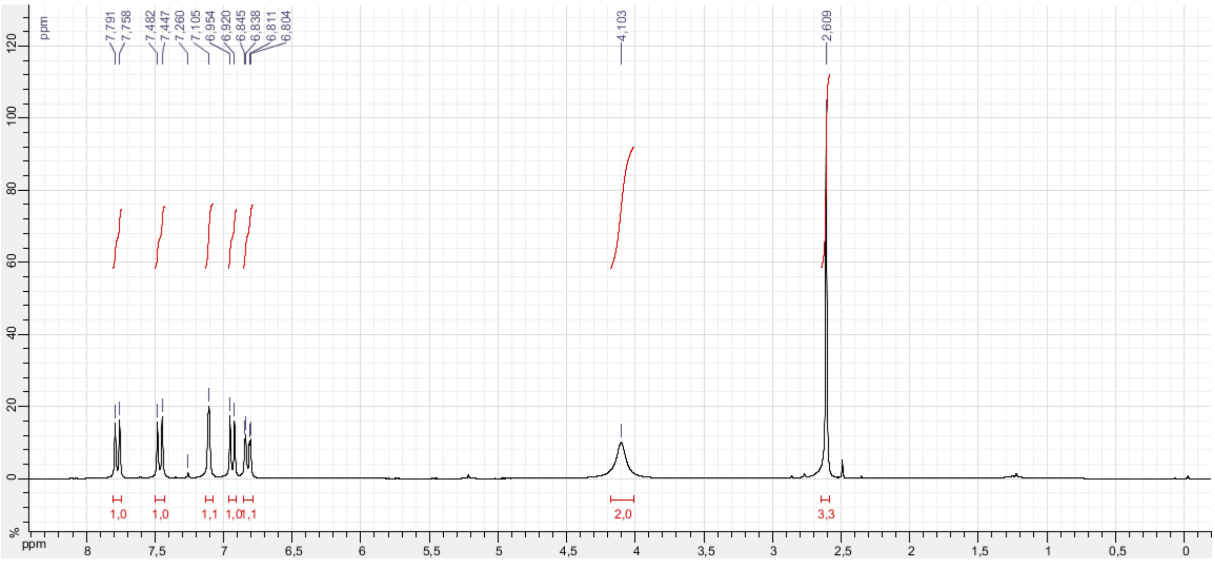
ppm
7,791
7,758
7,482
7,447
7,260
7,105
6,954
6,920
6,845
6,838
6,811
6,804
4,103
2,609
1,0
1,0
1,1
1,0
1,1
2,0
3,3
%
ppm
8
7,5
7
6,5
6
5,5
5
4,5
4
3,5
3
2,5
2
1,5
1
0,5
0
120
100
80
60
40
20
0

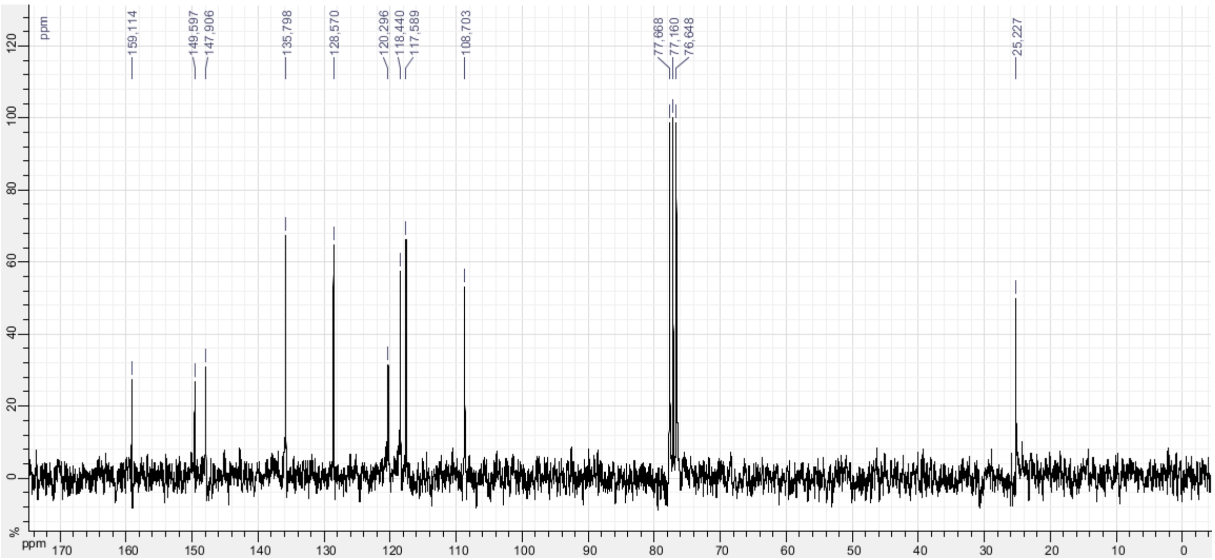
ppm
159,114
149,597
147,906
135,798
128,570
120,296
118,440
117,589
108,703
77,668
77,160
76,648
25,227
%
ppm
170
160
150
140
130
120
110
100
90
80
70
60
50
40
30
20
10
0
120
100
80
60
40
20
0

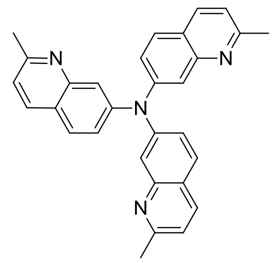
N
N
N
N

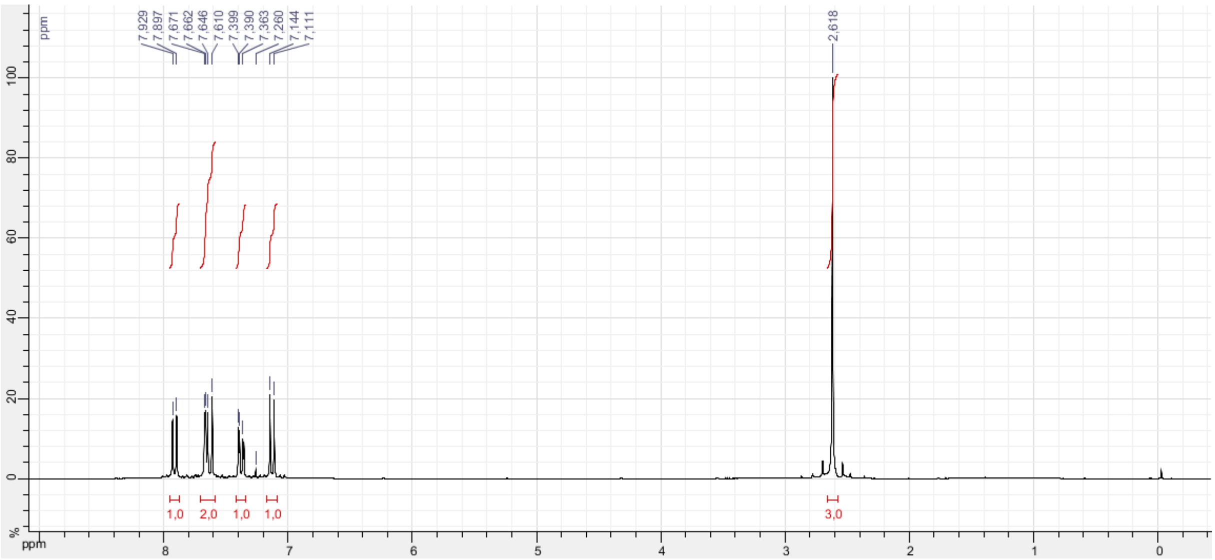
ppm
7,929
7,897
7,671
7,662
7,646
7,610
7,399
7,390
7,363
7,260
7,144
7,111
2,618
1,0
2,0
1,0
1,0
3,0

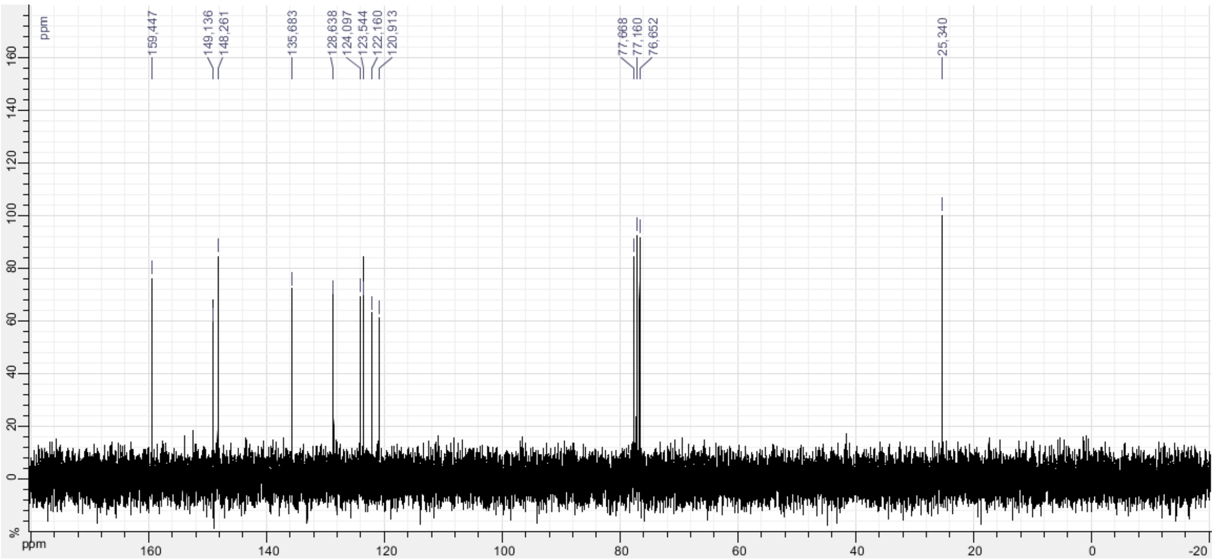
ppm
159,447
149,136
148,261
135,683
128,638
124,097
123,544
122,160
120,913
77,668
77,160
76,652
25,340

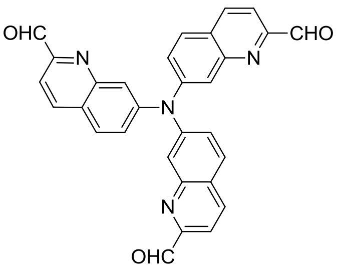
OHC
N
N
CHO
N
N
OHC

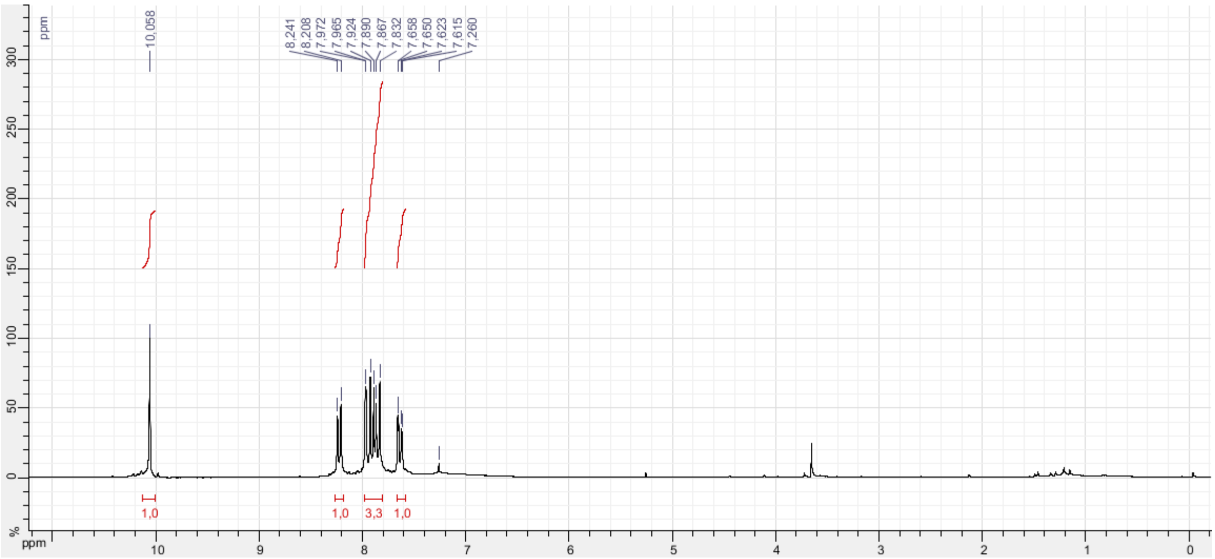
ppm
10,058
8,241
8,208
7,972
7,965
7,924
7,890
7,867
7,832
7,658
7,650
7,623
7,615
7,260
1,0
1,0
3,3
1,0
300
250
200
150
100
50
0
%
ppm
10
9
8
7
6
5
4
3
2
1
0

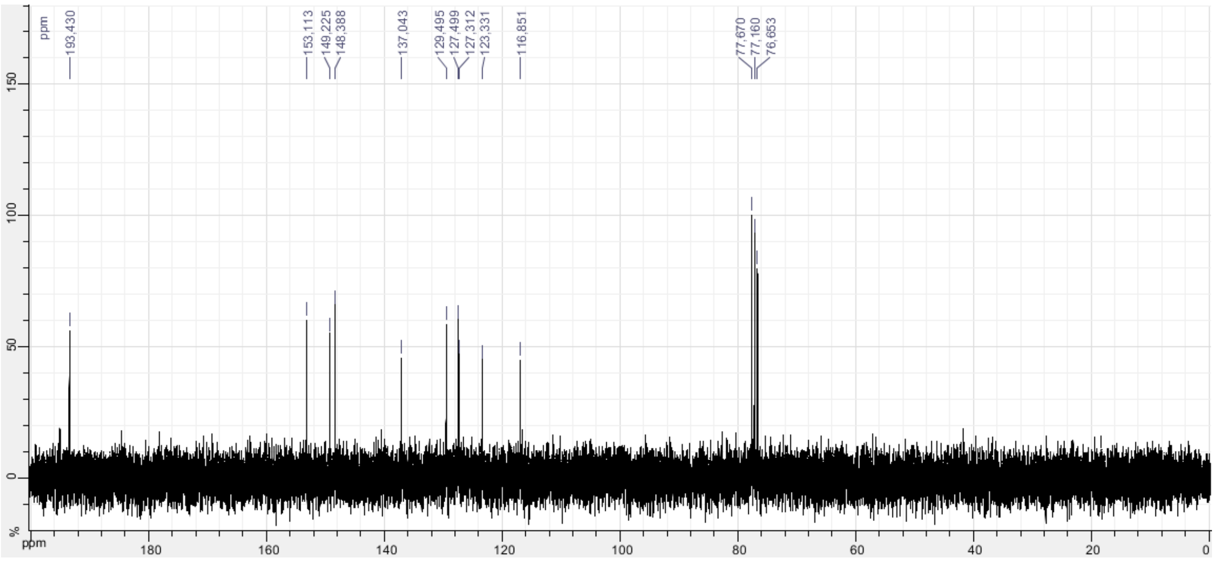
ppm
193,430
153,113
149,225
148,388
137,043
129,495
127,499
127,312
123,331
116,851
77,670
77,160
76,653
150
100
50
0
%
ppm
180
160
140
120
100
80
60
40
20
0

c) The Buchwald-Hartwig coupling strategy in the preparation of **3b**.

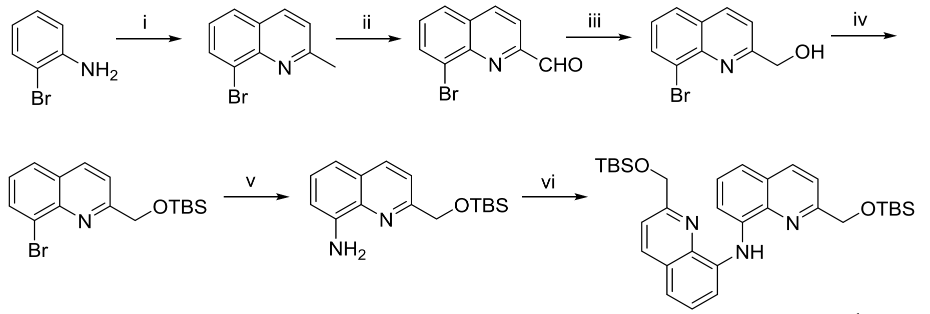

Scheme 3. Reagents and conditions: (i) crotonaldehyde (2.4 equiv), HCl (6M), toluene, 120°C, 3 h (78%)[1]; (ii) SeO_2 (1.1 equiv), dioxane, 80°C, 3 h (95%)[2]; (iii) $NaBH_4$ (1.2 equiv), EtOH, rt, 1 h (76%); (iv) TBSCl (1.1 equiv), imidazole (1.1 equiv), DMF, rt, 18 h (78%); (v) CuI (0.2 equiv), *L*-proline (0.4 equiv), K_2CO_3 (3.0 equiv), NH_4OH (aq. 28%, 10 equiv), DMSO, 80 °C, 18 h (25%); (vi) 8-bromo-2-(((*tert*-butyldimethylsilyl)oxy)methyl)quinoline (2.2 equiv), Pd_2dba_3 (0.2 equiv), $NaOC(CH_3)_3$ (2.2 equiv), P^tBu_3 (1M in toluene, 0.8 equiv), toluene, 110°C 18 h (83%).

8-Bromo-2-methylquinoline

2-Bromoaniline (5.0 g, 29.07 mmol, 1.0 equiv) was dissolved in a solution of HCl (6M, 140 mL). After addition of crotonaldehyde (5.74 mL, 69.87 mmol, 2.4 equiv) the mixture was stirred 1 h at room temperature. Then toluene (40 mL) was added and the reaction was heated at reflux temperature for 3 h. After cooling down to room temperature, the organic layer was removed. The aqueous layer was neutralized by adding NaOH, the solution was extracted with DCM and the organic layer was washed twice with water and brine, dried over Na_2SO_4, filtered and concentrated under reduced pressure. The crude product was purified by column chromatography on silica gel (cyclohexane:EtOAc 4:1). Beige solid (5.06 g, 78%). ^1H NMR (250 MHz, $CDCl_3$) δ 8.01-7.96 (m, 2H), 7.70 (dd, *J* = 8.0, 1.3 Hz, 1H), 7.30 (d, *J* = 8.0 Hz, 1H), 7.29 (t, *J* = 8.0 Hz, 1H), 2.80 (s, 3H). ^{13}C NMR (63 MHz, $CDCl_3$) δ 160.5, 144.9, 136.6, 133.1, 127.8, 127.5, 126.1, 124.3, 122.9, 25.8. MS (ESI): *m/z* = 222.0 $[M+H]^+$.

8-Bromoquinoline-2-carbaldehyde

A mixture of selenium dioxide (505 mg, 5.01 mmol, 1.1 equiv) and dioxane (15 mL) was heated at 80 °C for 30 min. 8-bromo-2-methylquinoline (1.00 g, 4.55 mmol, 1.0 equiv) was then introduced and the mixture was stirred for 3 h at 80 °C. After cooling down to room temperature, the mixture was filtered through cellite, eluted with DCM and the solvents were removed under reduced pressure. The crude product (beige solid, 1.02 g, 95%) was used without further purification. ^1H NMR (250 MHz, $CDCl_3$) δ 10.24 (d, *J* = 0.8 Hz, 1H), 8.24 (d, *J* = 8.5 Hz, 1H), 8.05 (dd, *J* = 7.5, 1.2 Hz, 1H), 7.97 (d, *J* = 8.3 Hz, 1H), 7.77 (dd, *J* = 8.3, 1.2 Hz, 1H), 7.44 (dd, *J* = 8.3, 7.5 Hz, 1H). ^{13}C NMR (63 MHz, $CDCl_3$) δ 193.3, 152.9, 144.9, 138.0, 134.1, 131.2, 129.4, 127.7, 126.1, 118.0. MS (ESI): *m/z* = 258.0 $[M+Na]^+$.

(8-Bromoquinolin-2-yl)methanol

To 8-bromoquinoline-2-carbaldehyde (1.08 g, 4.58 mmol, 1.0 equiv) in EtOH (7.5 mL) $NaBH_4$ (208 mg, 5.50 mmol, 1.2 equiv) was added at 0°C and the mixture was stirred at rt for 1 h before being quenched with 1M HCl. After removing the EtOH under reduced pressure, water was added to the residue, followed by extraction with DCM. The combined organic phases were washed twice with water and brine, dried over Na_2SO_4, filtered and evaporated to dryness. The crude product (beige solid, 825 mg, 76%) was used without further purification. ^1H NMR (250 MHz, $CDCl_3$) δ 8.13 (d, *J* = 8.5 Hz, 1H), 8.05 (d, *J* = 7.5 Hz, 1H), 7.79 (dd, *J* = 8.5 Hz, 1H), 7.40 (t, *J* = 7.5 Hz, 1H), 7.33 (d, *J* = 8.5 Hz, 1H), 4.95 (s, 2H), 4.68 (br s, 1H). ^{13}C NMR (63 MHz, $CDCl_3$) δ 160.0, 143.4, 137.3, 133.4, 128.7, 127.5, 126.7, 124.2, 119.1, 64.1. MS (ESI): *m/z* = 238.1, 241.1 $[M+H]^+$.

8-Bromo-2-(((*tert*-butyldimethylsilyl)oxy)methyl)quinoline

A solution of (8-bromoquinolin-2-yl)methanol (850 mg, 3.57 mmol, 1.0 equiv), TBSCl (592 mg, 3.93 mmol, 1.1 equiv) and imidazole (267 mg, 3.93 mmol, 1.1 equiv) in DMF (5 mL) was stirred at rt overnight, then the solvent was removed under reduced pressure. DCM was added and the organic phase was washed with water and brine, dried over Na_2SO_4, filtered and concentrated under reduced

---

[1] W. E. Childers, L. M. Havran, M. Asselin, J. J. Bicksler, D. C. Chong, G. T. Grosu, Z. Shen, A. Abou-Gharbia, A. C. Bach, B. L. Harrison, N. Kagan, T. Kleintop, R. Magolda, V. Marathias, A. J. Robichaud, A. L. Sabb, M.-Y. Zhang, T. H. Andree, S. H. Aschmies, C. Beyer, T. A. Comery, M. Day, S. M. Grauer, Z. A. Hughes, S. Rosenzweig-Lipson, B. Platt, C. Pulicicchio, D. E. Smith, S. J. Sukoff-Rizzo, K. M. Sullivan, A. Adedoyin, C. Huselton, W. D. Hirs, *J. Med. Chem.* **2010**, *53*, 4066-4084

[2] D. L. M. Suess, J. C. Peters, *Chem. Commun.,* **2010**, *46*, 6554–6556

pressure. The crude product was purified by column chromatography on silica gel (cyclohexane:EtOAc 9:1) to afford 984 mg (78%) of the corresponding protected alcohol as a pale yellow solid. ^{1}H NMR (250 MHz, $CDCl_3$) δ 8.12 (d, J = 8.5 Hz, 1H), 8.00 (dd, J = 7.5, 1.3 Hz, 1H), 7.75 (d, J = 8.5 Hz, 1H), 7.71 (dd, J = 8.5, 1.3 Hz, 1H), 7.29 (t, J = 7.5 Hz, 1H), 5.10 (s, 2H), 1.01 (s, 9H), 0.19 (s, 6H). ^{13}C NMR (63 MHz, $CDCl_3$) δ 163.1, 144.3, 137.0, 133.0, 128.6, 127.6, 126.2, 124.4, 119.2, 66.7, 25.9, 18.3, -5.3. MS (ESI): *m/z* = 352.2, 354.2 $[M+H]^+$. HRMS (ESI): *m*/*z* calcd for $[C_{16}H_{22}NOBrSi+H]^+$ 352.0727 and 354.0704, found 352.0744 and 354.0724.

2-(((*tert*-Butyldimethylsilyl)oxy)methyl)quinolin-8-amine

8-Bromo-2-(((*tert*-butyldimethylsilyl)oxy)methyl)quinoline (400 mg, 1.14 mmol, 1.0 equiv), copper iodide (44 mg, 0.23 mmol, 20 mol%), *L*-proline (52 mg, 0.46 mmol, 40 mol%) and K_2CO_3 (472 mg, 3.42 mmol, 3.0 equiv) were dissolved in DMSO (2 mL). Aqueous ammonia (28%) (1.1 mL, 11.40 mmol, 10 eq) was then introduced and the mixture was heated at 80°C for 18 h. After cooling to room temperature, DCM was added, followed by saturated NH_4Cl solution. The aqueous layer was extracted twice with DCM and the combined organic layers were washed again with saturated NH_4Cl solution. The product was purified by column chromatography on silica gel (cyclohexane:EtOAc 4:1). Yellow solid (81 mg, 25%). ^{1}H NMR (250 MHz, $CDCl_3$) δ 7.98 (d, J=9.0 Hz, 1H), 7.56 (d, J=8.5 Hz, 1H), 7.18 (dm, J=7.5 Hz, 1H), 7.04 (dd, J=8.5, 1.0 Hz, 1H), 6.80 (dd, J=7.5, 1.5 Hz, 1H), 4.93 (s, 2H), 4.90 (brs, 2H), 0.94 (s, 9H), 0.10 (s, 6H). ^{13}C NMR (63 MHz, $CDCl_3$) δ 158.7, 143.6, 136.5, 133.0, 127.8, 126.7, 118.8, 115.9, 110.0, 66-9. 26.0, 18.4, -5.2.

Bis(2-(((*tert*-butyldimethylsilyl)oxy)methyl)quinolin-8-yl)amine

In a sealed tube placed in a glove box, 2-(((*tert*-butyldimethylsilyl)oxy)methyl)quinolin-8-amine (27 mg, 0.09 mmol, 1.0 equiv), 8-bromo-2-(((*tert*-butyldimethylsilyl)oxy)methyl)quinoline (70 mg, 0.20 mmol, 2.2 equiv), Pd_2dba_3 (20 mg, 0.02 mmol, 20 mol%) and $NaOC(CH_3)_3$ (18 mg, 0.20 mmol, 2.2 equiv) were introduced. 1M solution (in toluene) of tri-*tert*-butylphosphine (15 µL, 0.07 mmol, 80 mol%) and distilled toluene (500 µL) were added and the tube was sealed. The mixture was heated at 110°C for 18 h. After cooling to room temperature, DCM was added and the organic layer was washed twice with water and brine, dried over Na_2SO_4, and concentrated under reduced pressure. The crude product was purified by column chromatography on silica gel (cyclohexane:EtOAc 4:1), to yield the dimer product as a yellow oil (42 mg, 83%). ^{1}H NMR (250 MHz, $CDCl_3$) δ 10.74 (s, 1H), 8.18 (d, J = 8.3 Hz, 2H), 7.87 (dd, J = 7.5, 1.0 Hz, 2H), 7.76 (d, J = 8.3 Hz, 2H), 7.49 (t, J = 8.3 Hz, 2H), 7.32 (dd, J = 8.3, 1.0 Hz, 2H), 5.15 (s, 4H), 1.04 (s, 18H), 0.22 (s, 12H). ^{13}C NMR (63 MHz, $CDCl_3$) δ 159.3, 139.0, 138.6, 136.9, 128.1, 126.7, 119.2, 117.6, 109.9, 67.3, 26.1, 18.6, -5.0. MS (ESI): *m/z* = 560.5 $[M+H]^+$. HRMS (ESI): *m*/*z* calcd for $[C_{32}H_{45}N_3O_2Si_2+H]^+$ 560.3123, found 560.3110.

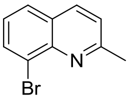
N
Br

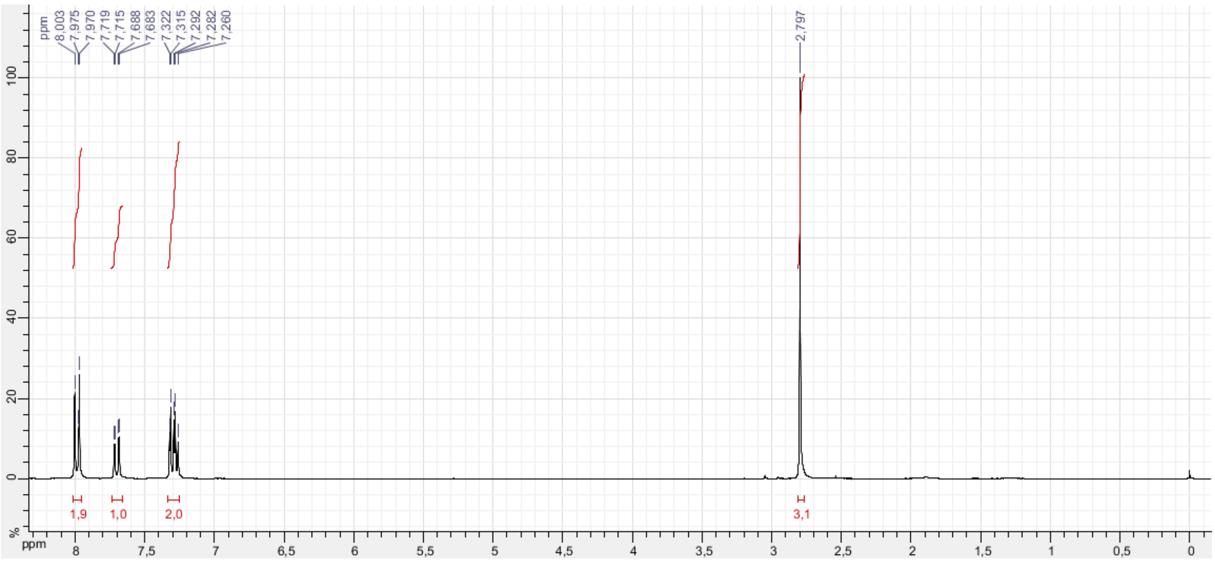
ppm
8,003
7,975
7,970
7,719
7,715
7,688
7,683
7,322
7,315
7,292
7,282
7,260
2,797
1,9
1,0
2,0
3,1
%
ppm

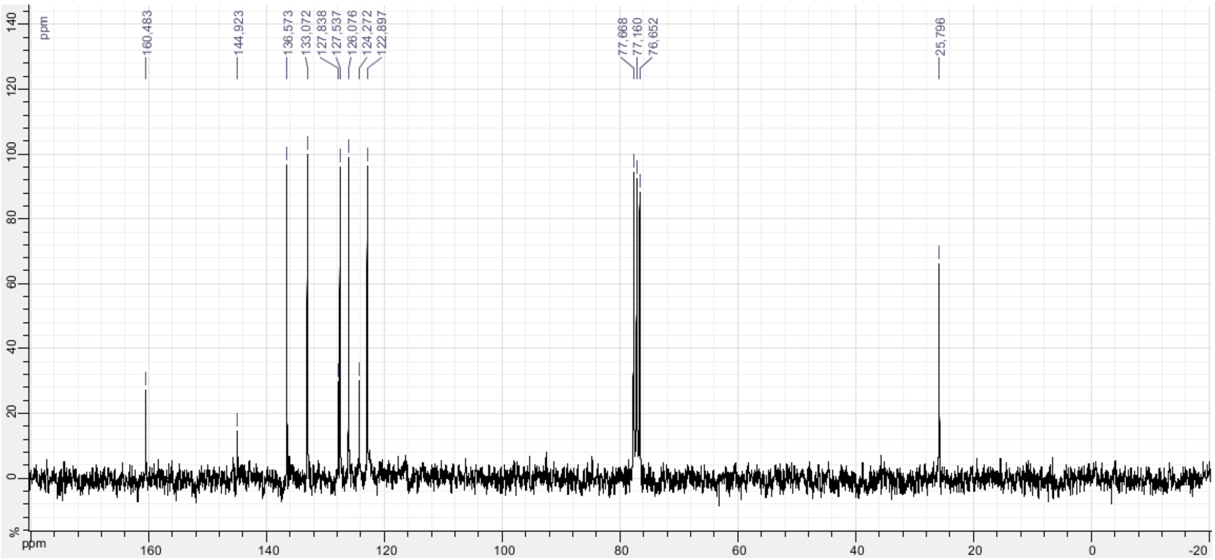
ppm
160,483
144,923
136,573
133,072
127,838
127,537
126,076
124,272
122,897
77,668
77,160
76,652
25,796
%
ppm

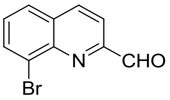
N
CHO
Br

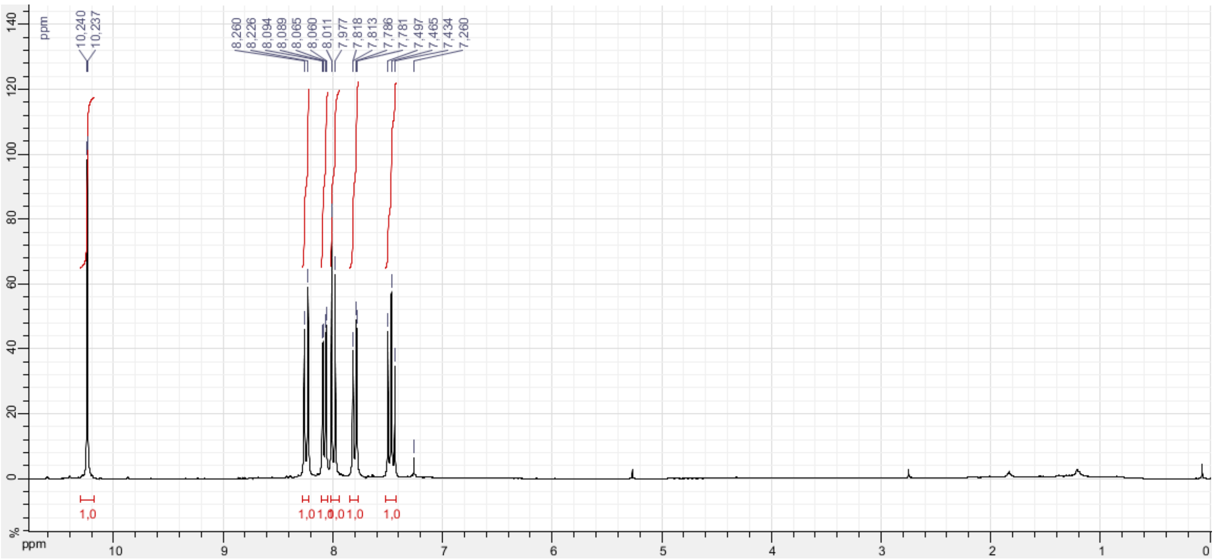
ppm
10,240
10,237
8,260
8,226
8,094
8,089
8,065
8,060
8,011
7,977
7,818
7,813
7,786
7,781
7,497
7,465
7,434
7,260
1,0
1,0
1,0
1,0
1,0
1,0

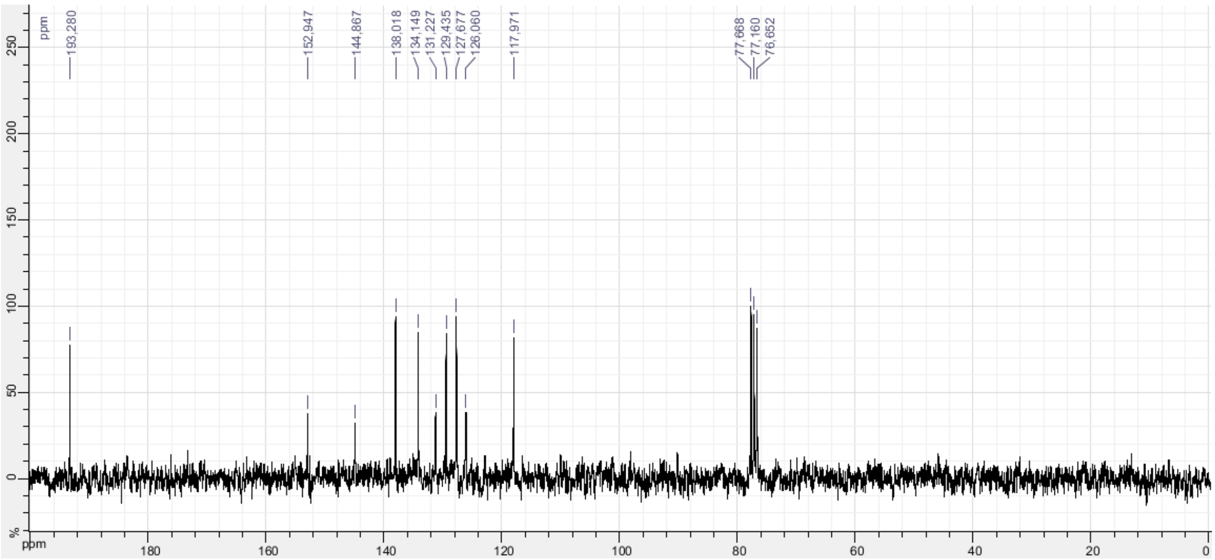
ppm
193,280
152,947
144,867
138,018
134,149
131,227
129,435
127,677
126,060
117,971
77,668
77,160
76,652

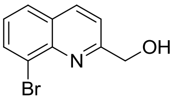
N
OH
Br

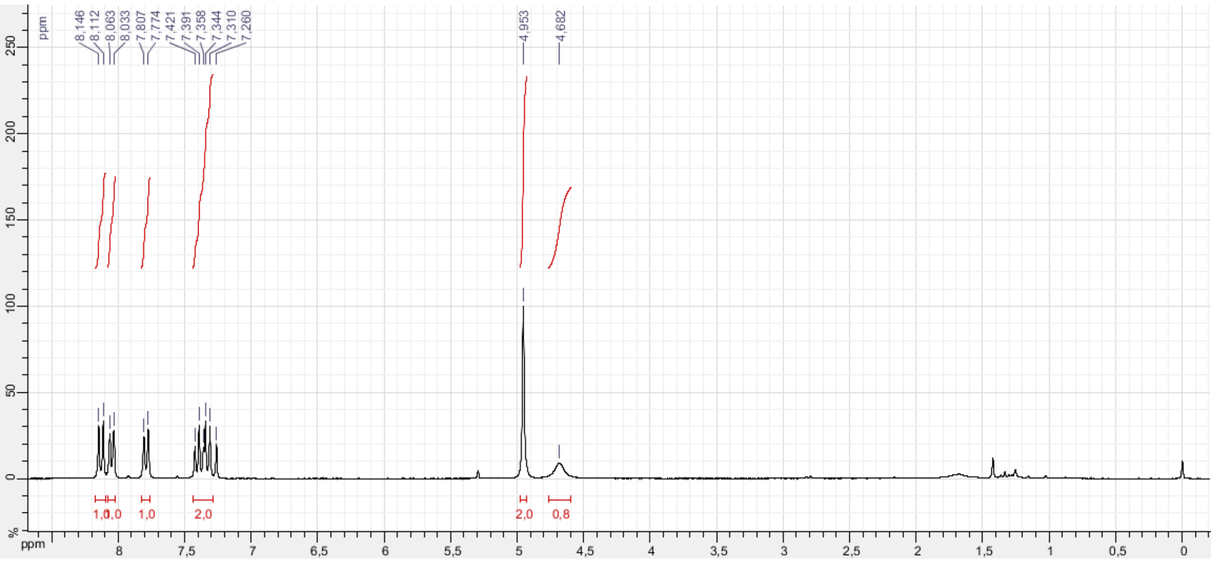
ppm
8,146
8,112
8,063
8,033
7,807
7,774
7,421
7,391
7,358
7,344
7,310
7,260
4,953
4,682
1,0
1,0
1,0
2,0
2,0
0,8
%
ppm

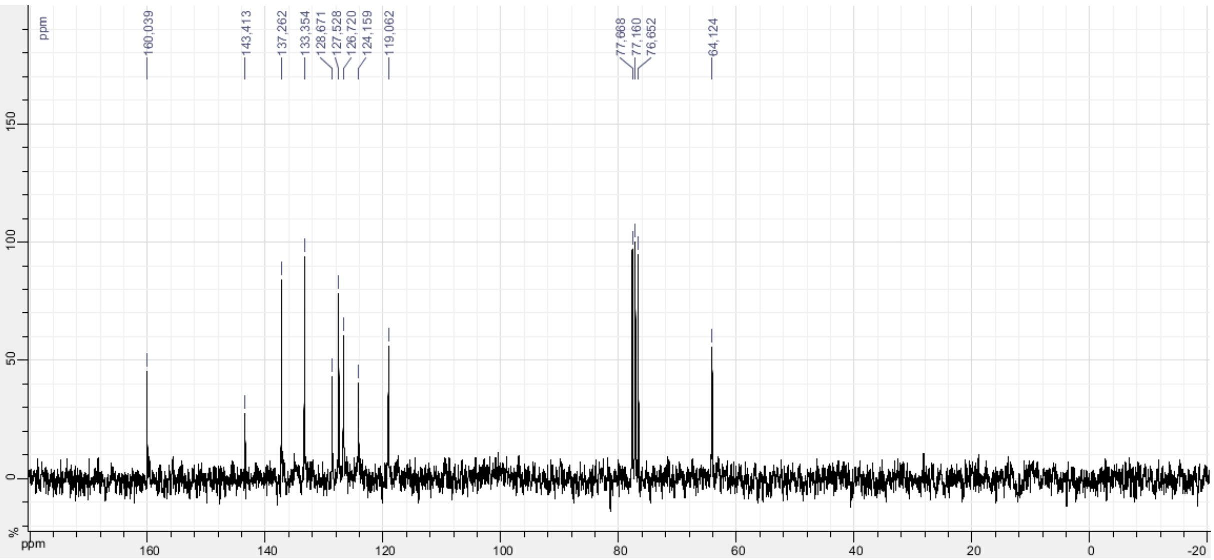
ppm
160,039
143,413
137,262
133,354
128,671
127,528
126,720
124,159
119,062
77,668
77,160
76,652
64,124
%
ppm

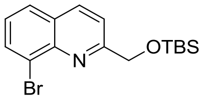
OTBS
N
Br

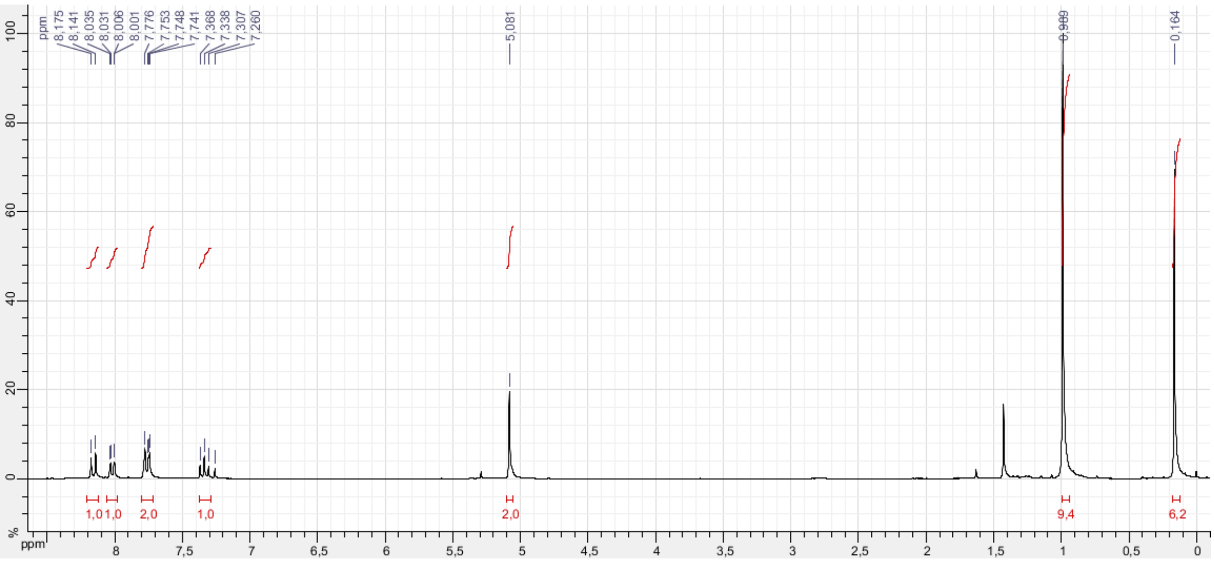
ppm
8,175
8,141
8,035
8,031
8,006
8,001
7,776
7,753
7,748
7,741
7,368
7,338
7,307
7,260
5,081
0,164
1,0
1,0
2,0
1,0
2,0
9,4
6,2
%
ppm
8
7,5
7
6,5
6
5,5
5
4,5
4
3,5
3
2,5
2
1,5
1
0,5
0

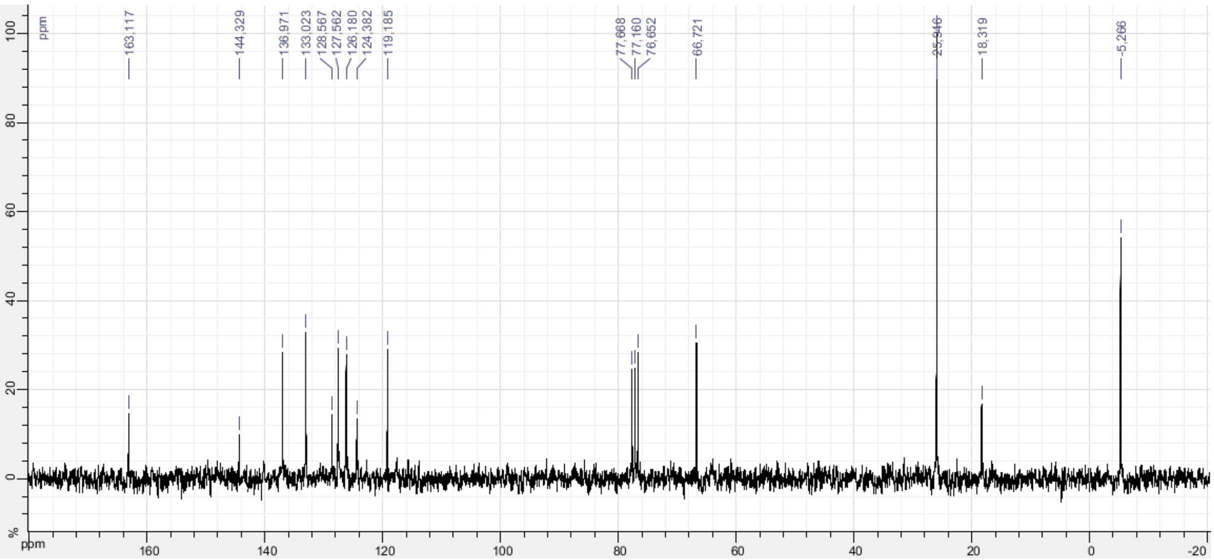
ppm
163,117
144,329
136,971
133,023
128,567
127,562
126,180
124,382
119,185
77,668
77,160
76,652
66,721
18,319
-5,266
%
ppm
160
140
120
100
80
60
40
20
0
-20

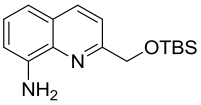
N
OTBS
NH_2

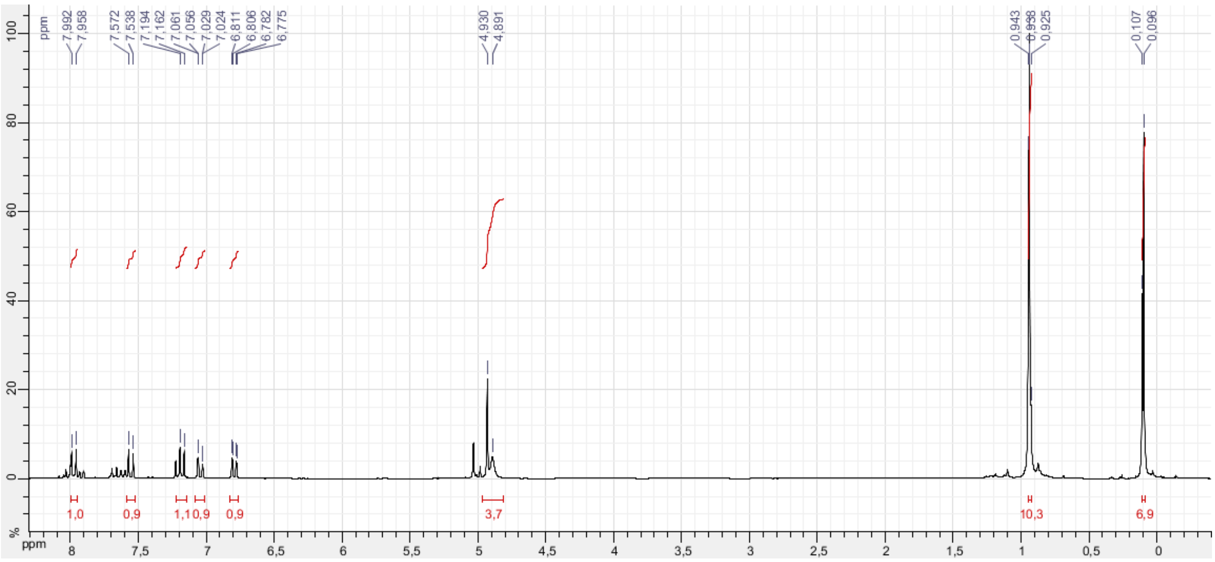
7,992
7,958
7,572
7,538
7,194
7,162
7,061
7,056
7,029
7,024
6,811
6,806
6,782
6,775
4,930
4,891
0,943
0,938
0,925
0,107
0,096
1,0
0,9
1,1
0,9
0,9
3,7
10,3
6,9
ppm
%

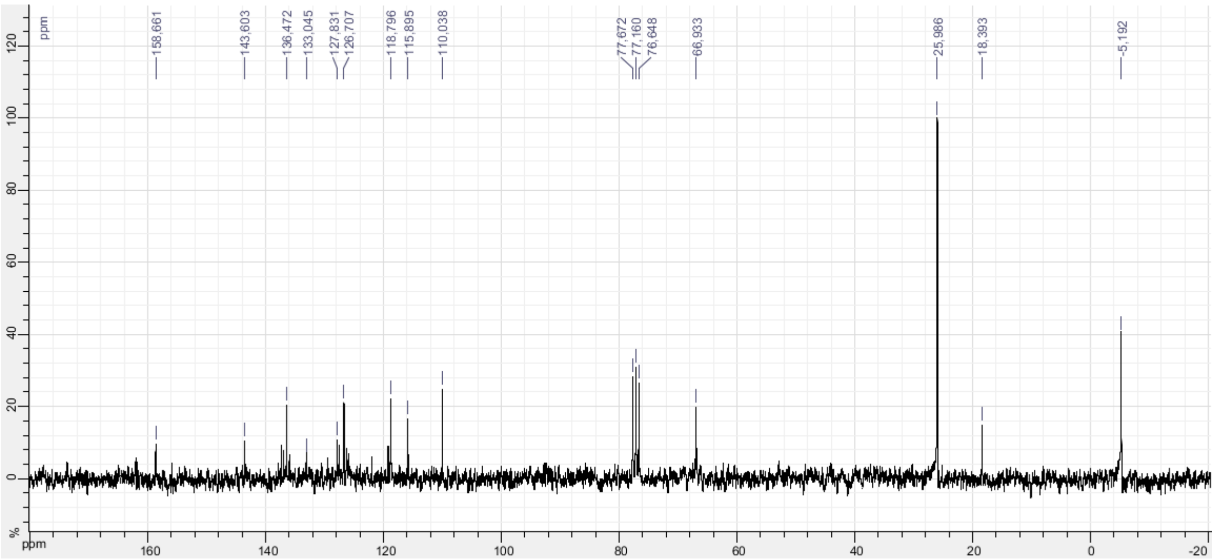
158,661
143,603
136,472
133,045
127,831
126,707
118,796
115,895
110,038
77,672
77,160
76,648
66,933
25,986
18,393
-5,192
ppm
%

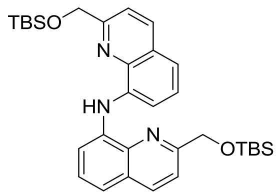
TBSO
N
HN
N
OTBS

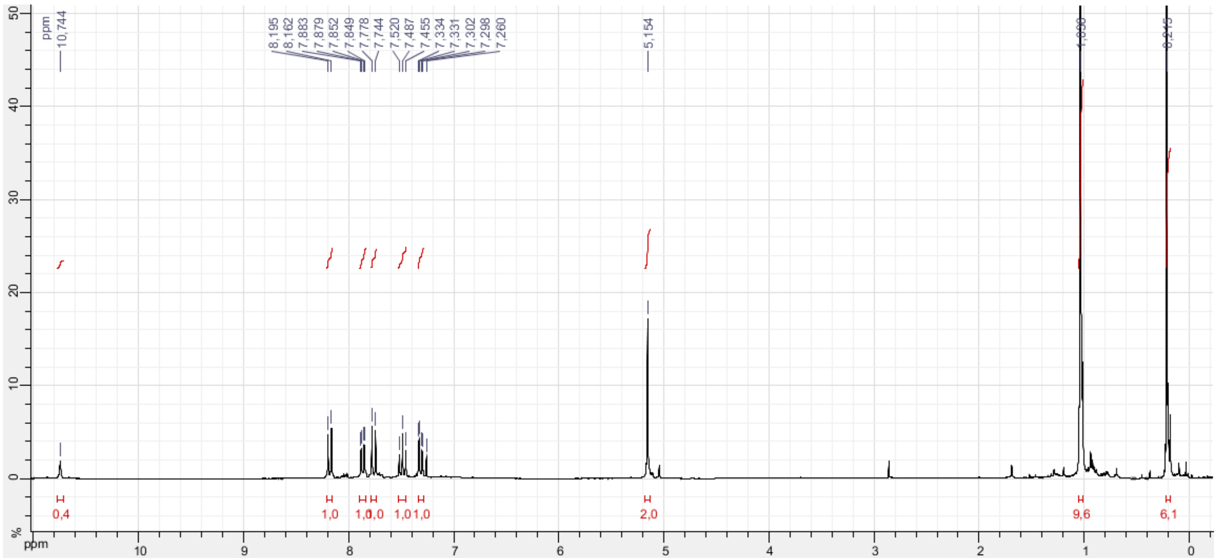
ppm
10,744
8,195
8,162
7,883
7,879
7,852
7,849
7,778
7,744
7,520
7,487
7,455
7,334
7,331
7,302
7,298
7,260
5,154
0,4
1,0
1,0
1,0
1,0
1,0
2,0
9,6
6,1

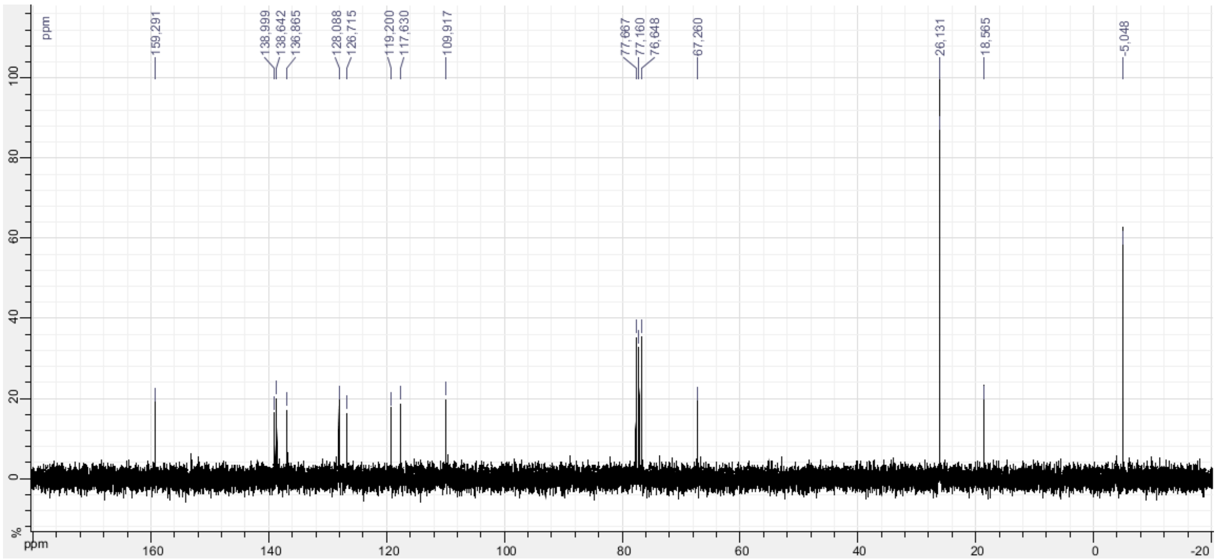
ppm
159,291
138,999
138,642
136,865
128,088
126,715
119,200
117,630
109,917
77,667
77,160
76,648
67,260
26,131
18,565
-5,048

5. ^{1}H and ^{13}C NMR spectra of all novel compounds

Compound 5

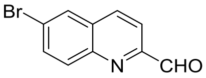

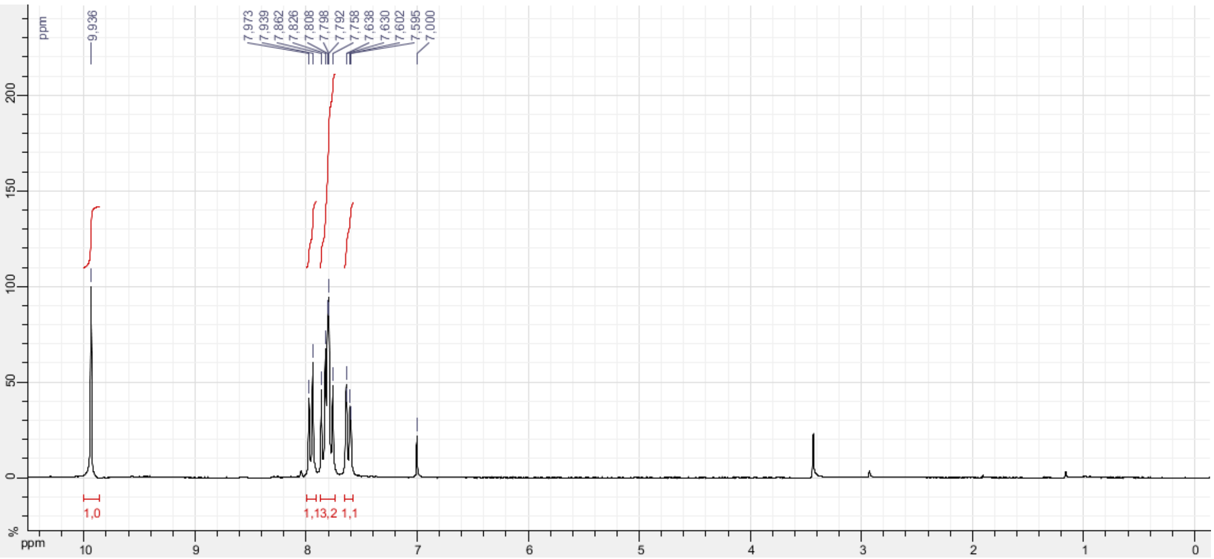

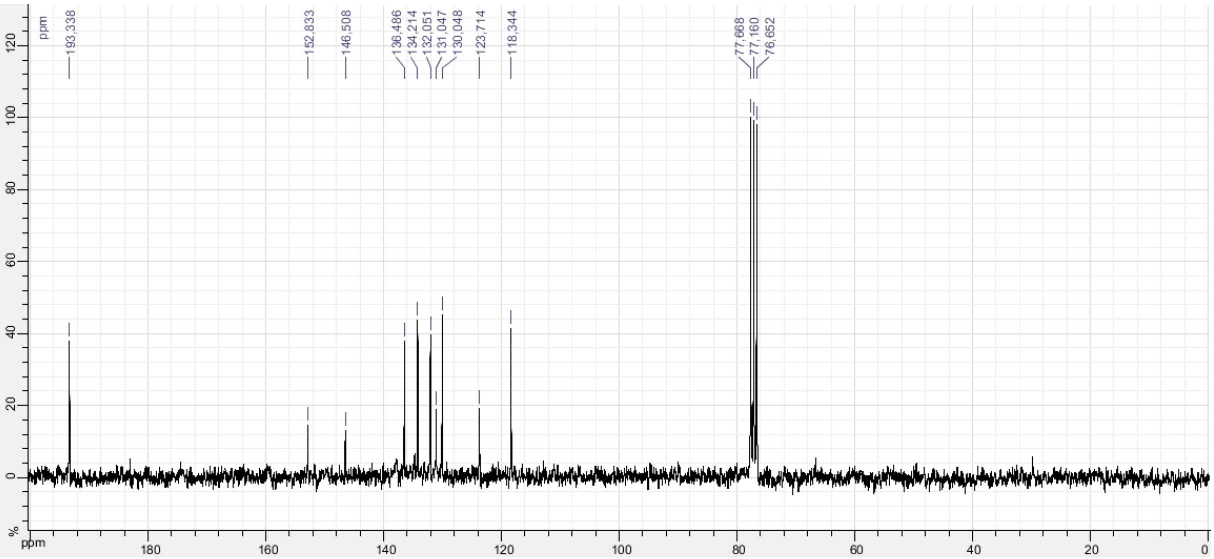

Compound 6

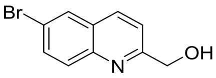

Br
N
OH

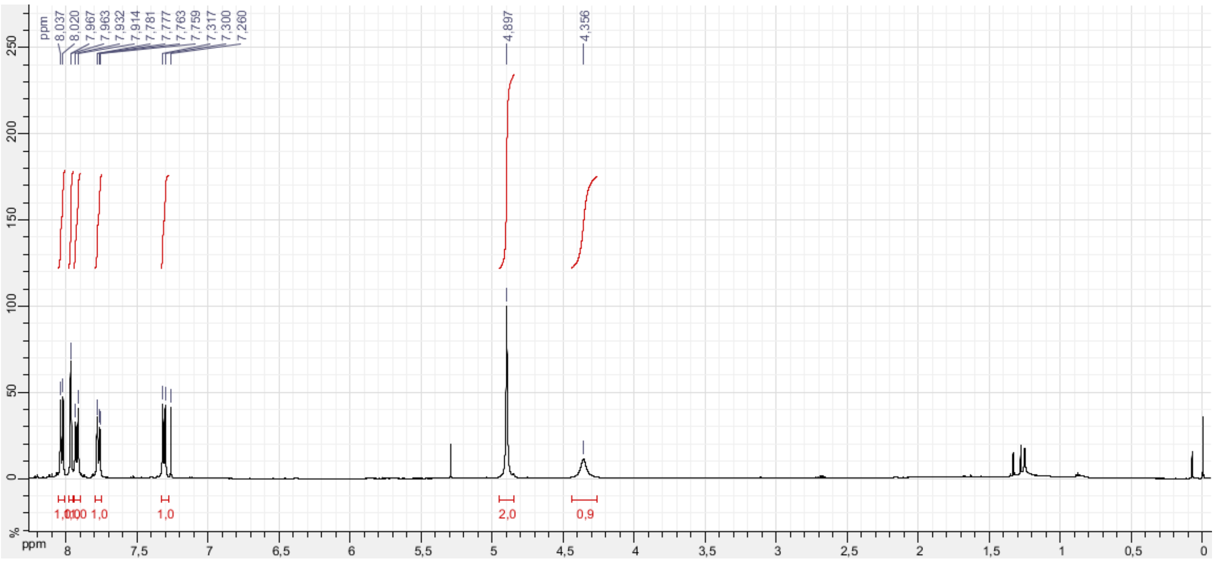

ppm
8,037
8,020
7,967
7,963
7,932
7,914
7,781
7,777
7,763
7,759
7,317
7,300
7,260
4,897
4,356
1,0
1,0
1,0
1,0
1,0
2,0
0,9

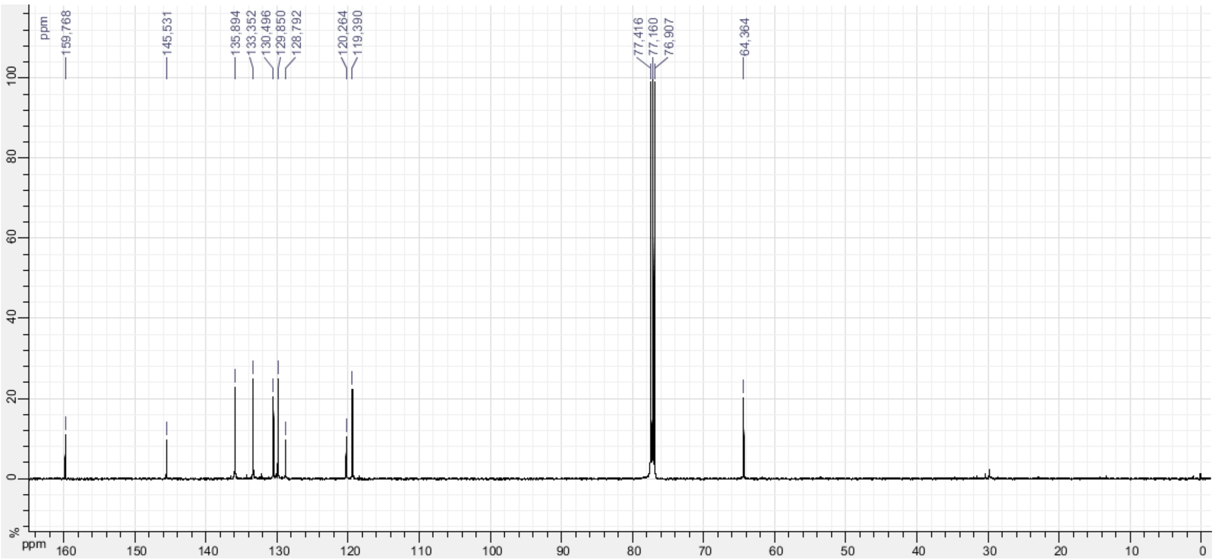

ppm
159,768
145,531
135,894
133,352
130,496
129,850
128,792
120,264
119,390
77,416
77,160
76,907
64,364

Compound 7

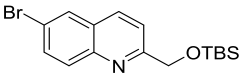
Br
N
OTBS

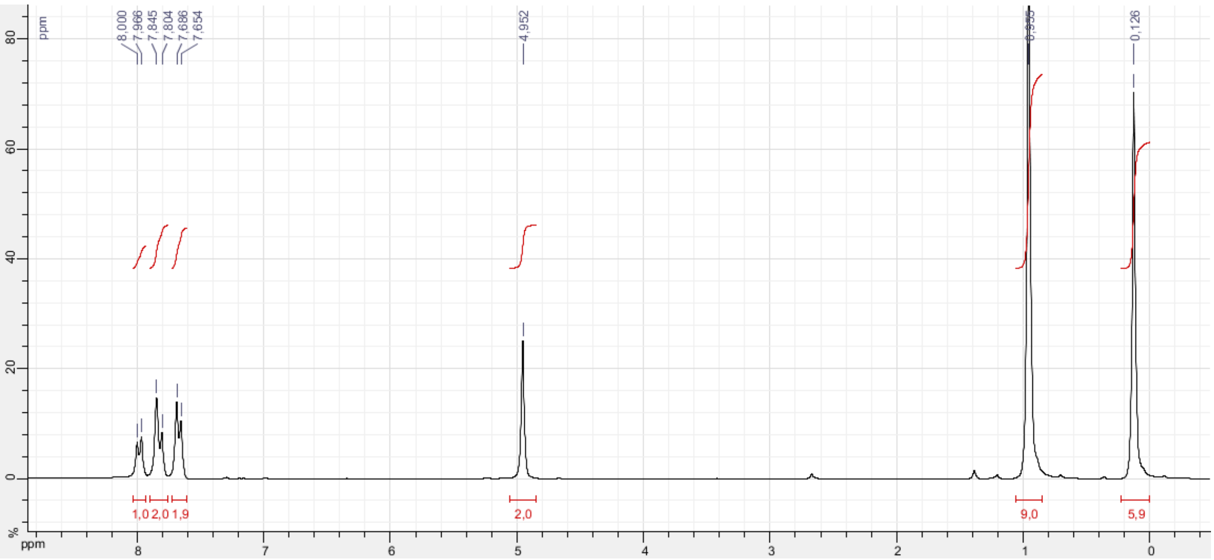
8,000
7,966
7,845
7,804
7,686
7,654
4,952
0,126
1,0
2,0
1,9
2,0
9,0
5,9
ppm
%

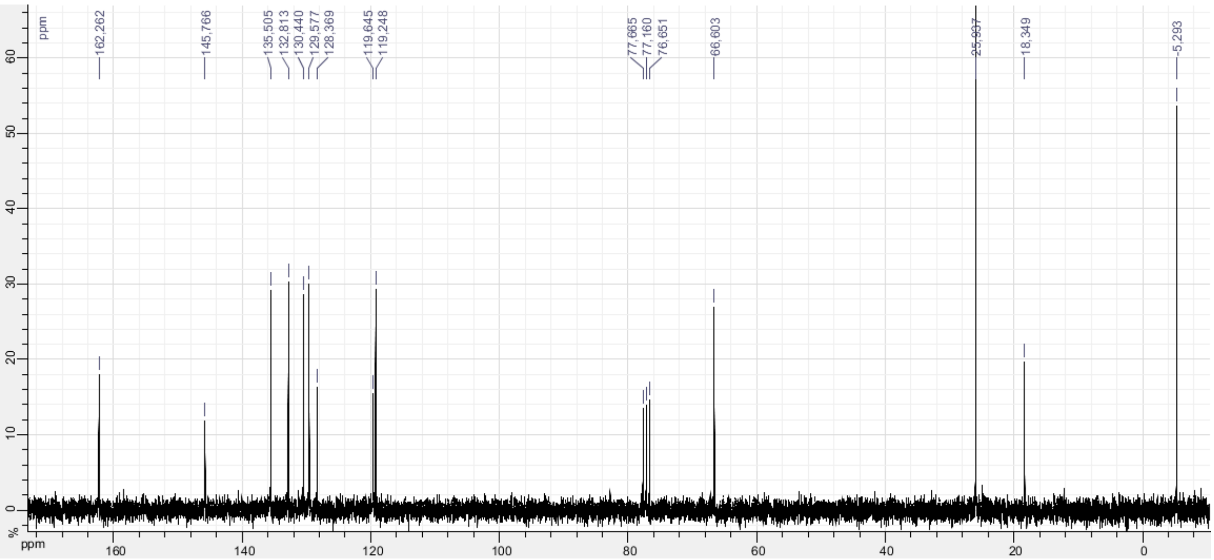
162,262
145,766
135,505
132,813
130,440
129,577
128,369
119,645
119,248
77,665
77,160
76,651
66,603
18,349
-5,293
ppm
%

Compound 8

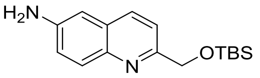
H2N
N
OTBS

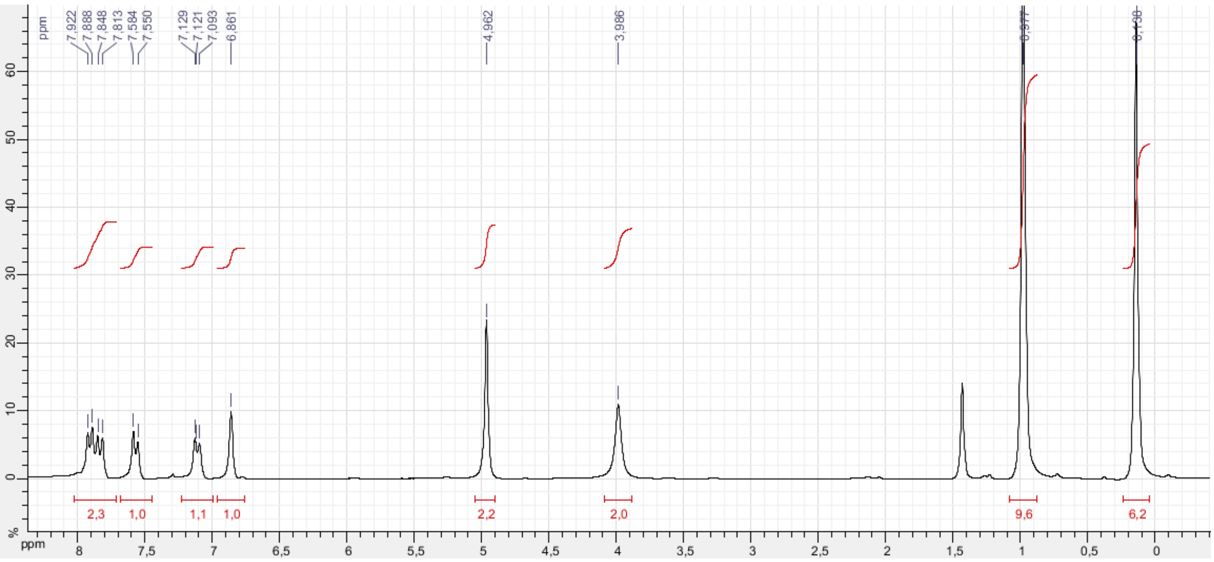
ppm
7,922
7,888
7,848
7,813
7,584
7,550
7,129
7,121
7,093
6,861
4,962
3,986
2,3
1,0
1,1
1,0
2,2
2,0
9,6
6,2

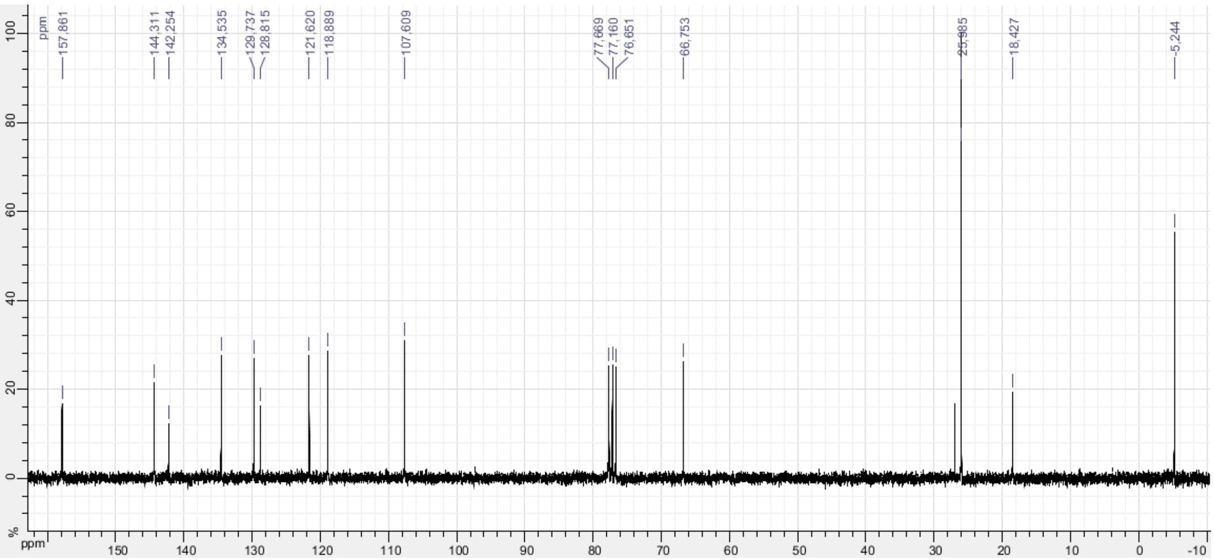
ppm
157,861
144,311
142,254
134,535
129,737
128,815
121,620
118,889
107,609
77,669
77,160
76,651
66,753
25,985
18,427
-5,244

Compound 9

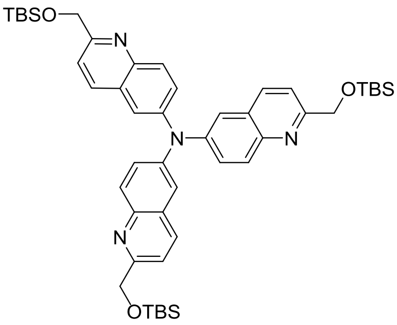
TBSO
N
OTBS
N
N
N
OTBS

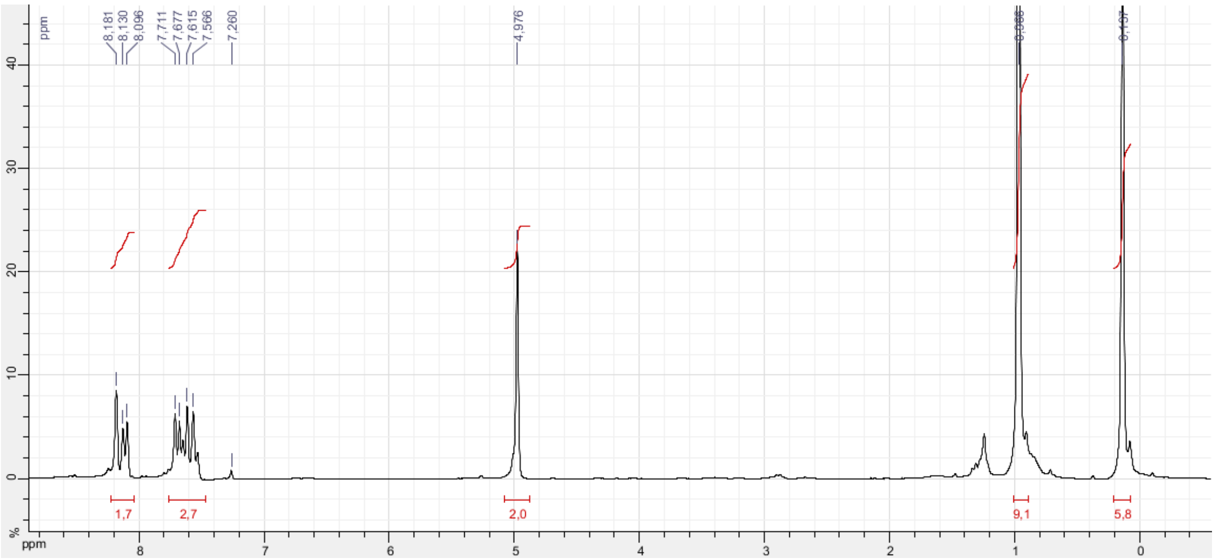
ppm
8,181
8,130
8,096
7,711
7,677
7,615
7,566
7,260
4,976
1,7
2,7
2,0
9,1
5,8
%

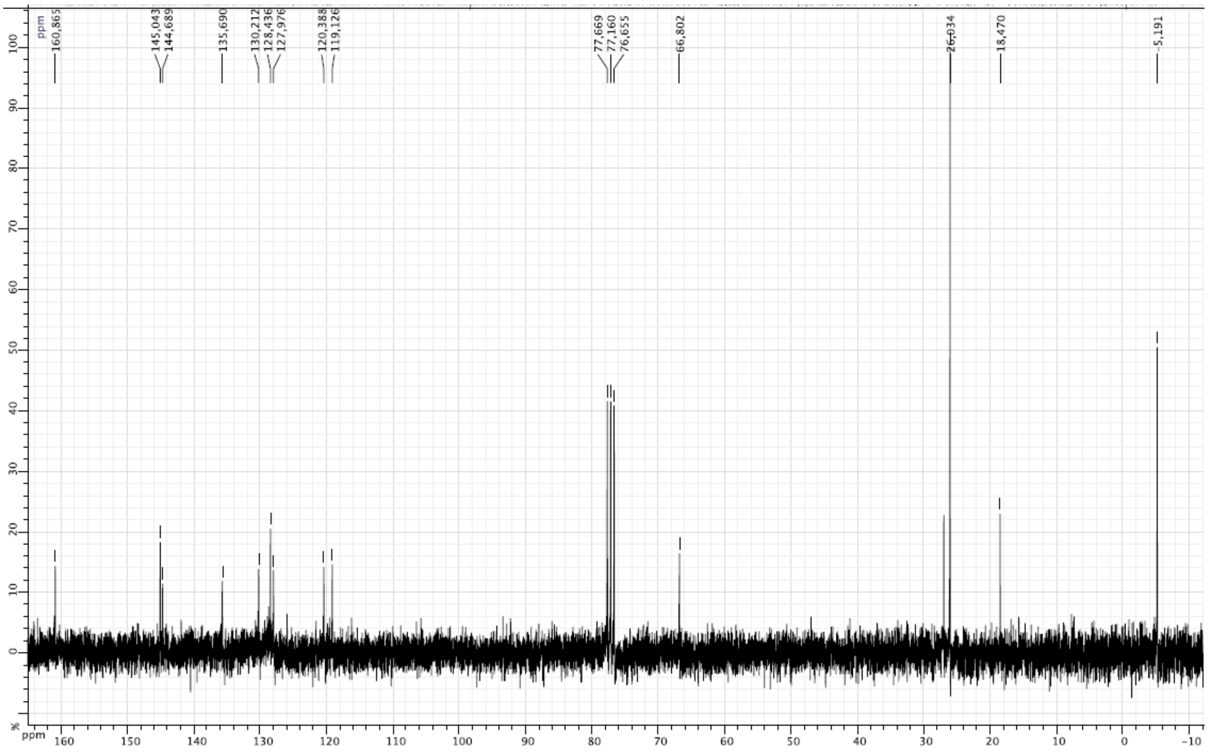
ppm
160,865
145,043
144,689
135,690
130,212
128,436
127,976
120,388
119,126
77,669
77,160
76,655
66,802
26,034
18,470
-5,191
%

Compound 10

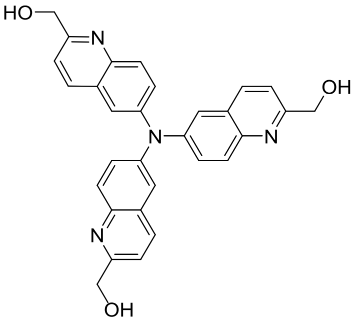
HO
N
OH
N
N
N
OH

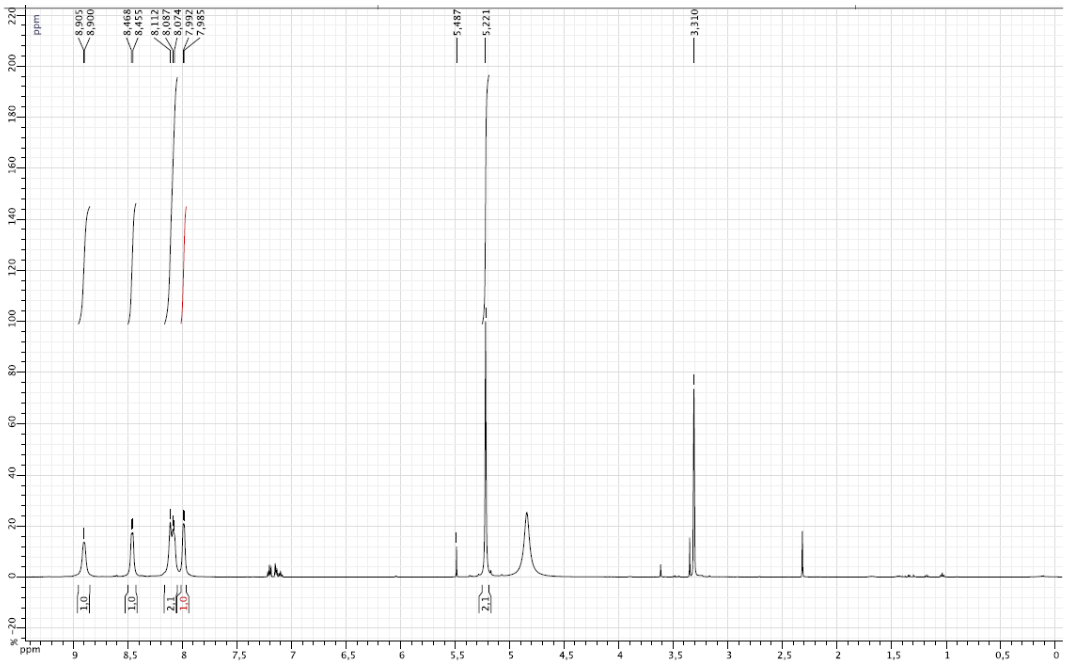
ppm
8,905
8,900
8,468
8,455
8,112
8,087
8,074
7,992
7,985
5,487
5,221
3,310
1,0
1,0
2,1
1,0
2,1
220
200
180
160
140
120
100
80
60
40
20
0
%
−20
9
8,5
8
7,5
7
6,5
6
5,5
5
4,5
4
3,5
3
2,5
2
1,5
1
0,5
0

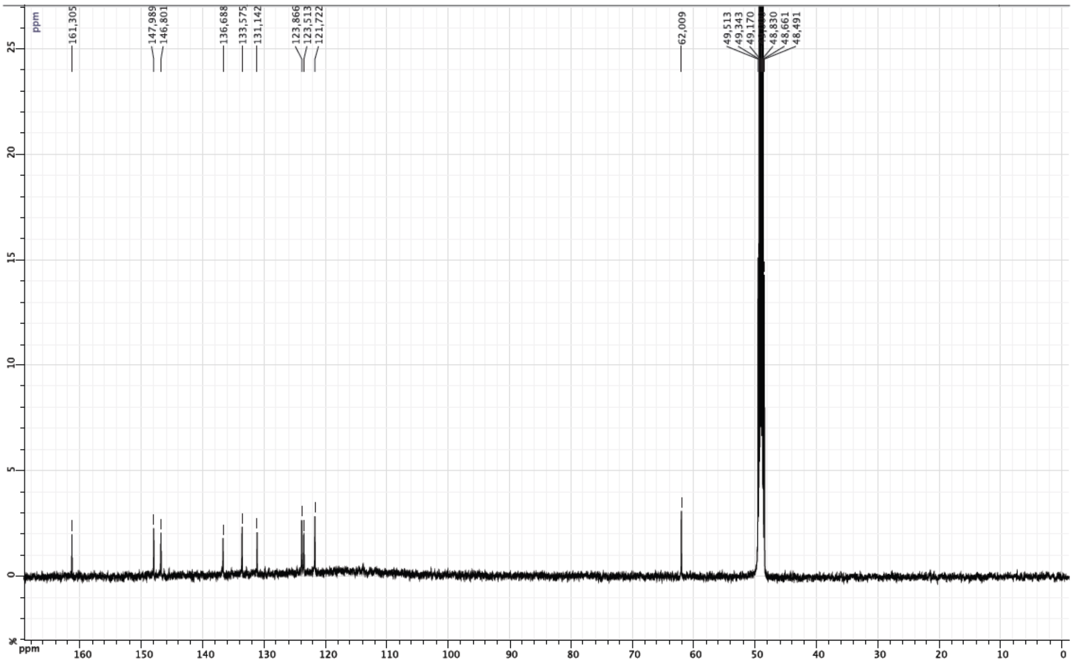
ppm
161,305
147,989
146,801
136,688
133,575
131,142
123,866
123,513
121,722
62,009
49,513
49,343
49,170
48,830
48,661
48,491
25
20
15
10
5
0
%
160
150
140
130
120
110
100
90
80
70
60
50
40
30
20
10
0

Compound 1b

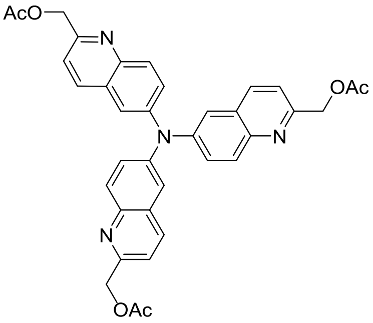
AcO
N
OAc
N
N
N
OAc

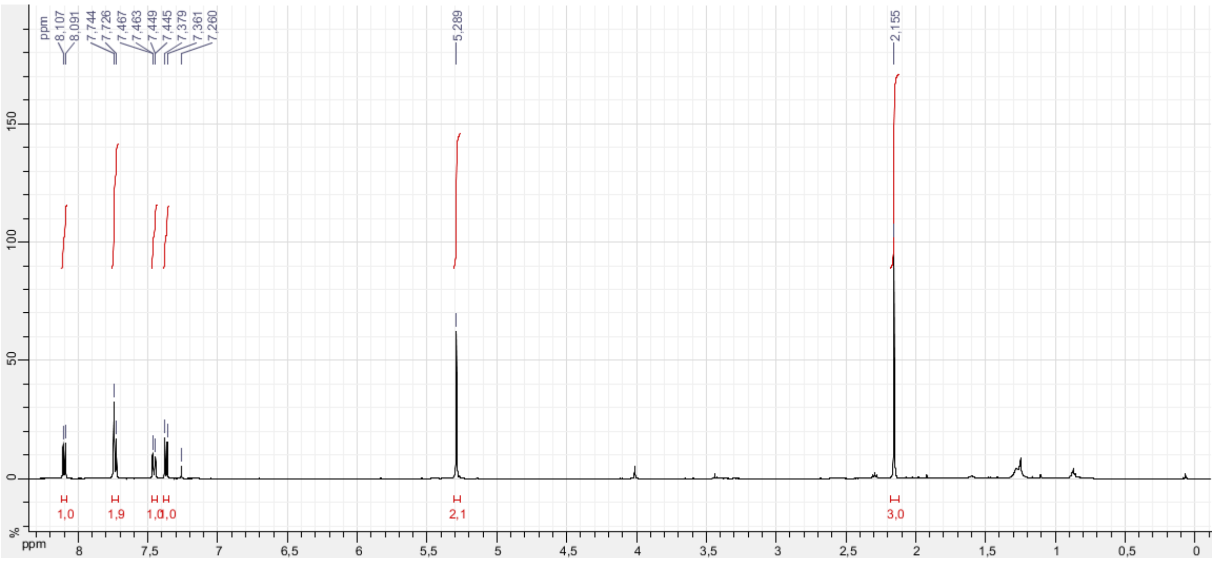
ppm
8,107
8,091
7,744
7,726
7,467
7,463
7,449
7,445
7,379
7,361
7,260
5,289
2,155
1,0
1,9
1,0
1,0
2,1
3,0
%
ppm

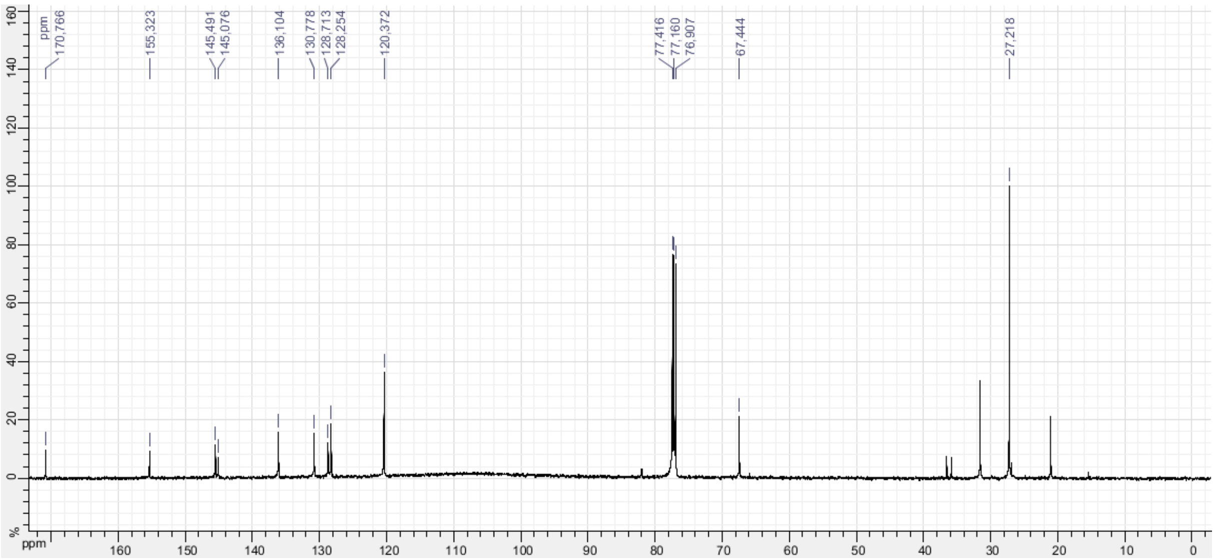
ppm
170,766
155,323
145,491
145,076
136,104
130,778
128,713
128,254
120,372
77,416
77,160
76,907
67,444
27,218
%
ppm

Compound 15

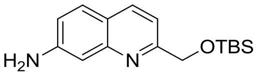
H2N
N
OTBS

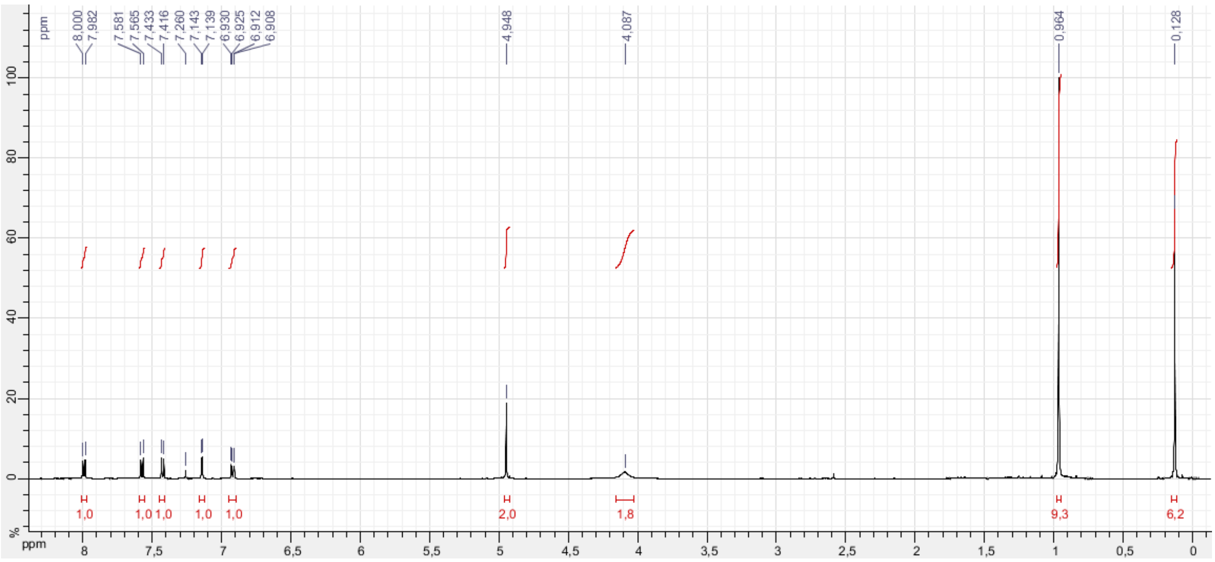
ppm
8,000
7,982
7,581
7,565
7,433
7,416
7,260
7,143
7,139
6,930
6,925
6,912
6,908
4,948
4,087
0,964
0,128
1,0 1,0 1,0 1,0 1,0 2,0 1,8 9,3 6,2
%
ppm
8 7,5 7 6,5 6 5,5 5 4,5 4 3,5 3 2,5 2 1,5 1 0,5 0

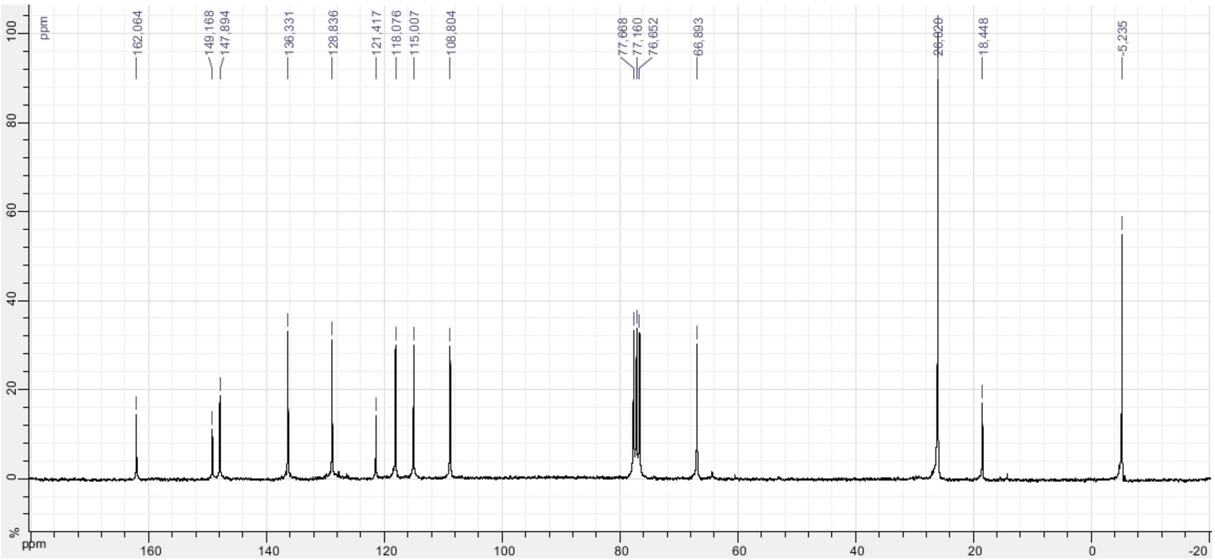
ppm
162,064
149,168
147,894
136,331
128,836
121,417
118,076
115,007
108,804
77,668
77,160
76,652
66,893
26,020
18,448
-5,235
%
ppm
160 140 120 100 80 60 40 20 0 -20

Compound 16

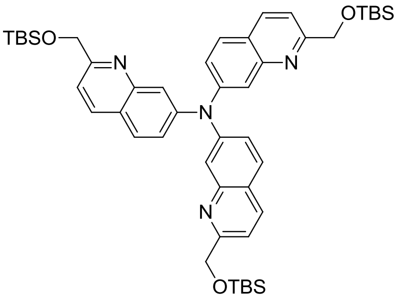
TBSO
N
N
N
OTBS
OTBS

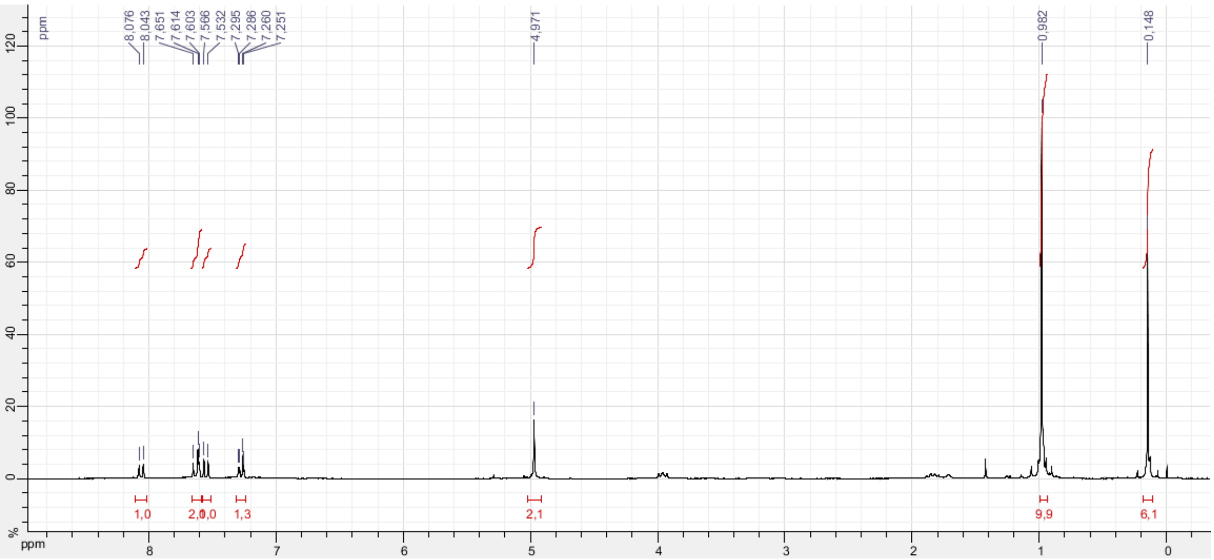
8,076
8,043
7,651
7,614
7,603
7,566
7,532
7,295
7,286
7,260
7,251
4,971
0,982
0,148
1,0
2,0
1,0
1,3
2,1
9,9
6,1
ppm
%

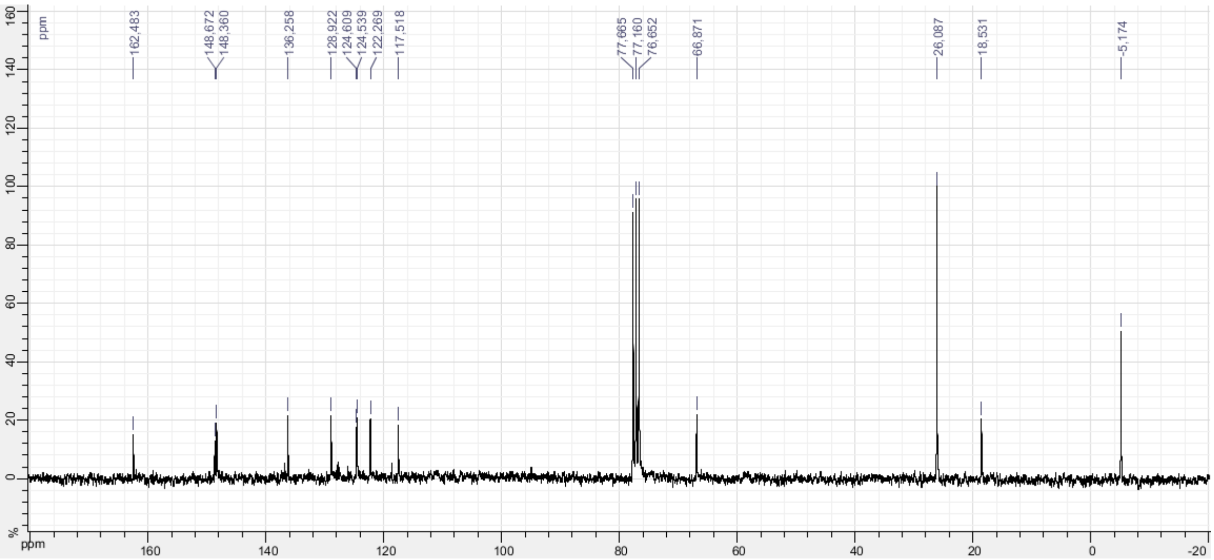
162,483
148,672
148,360
136,258
128,922
124,609
124,539
122,269
117,518
77,665
77,160
76,652
66,871
26,087
18,531
-5,174
ppm
%

Compound 17

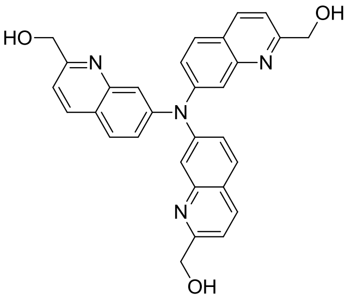

HO
N
OH
N
N
N
OH

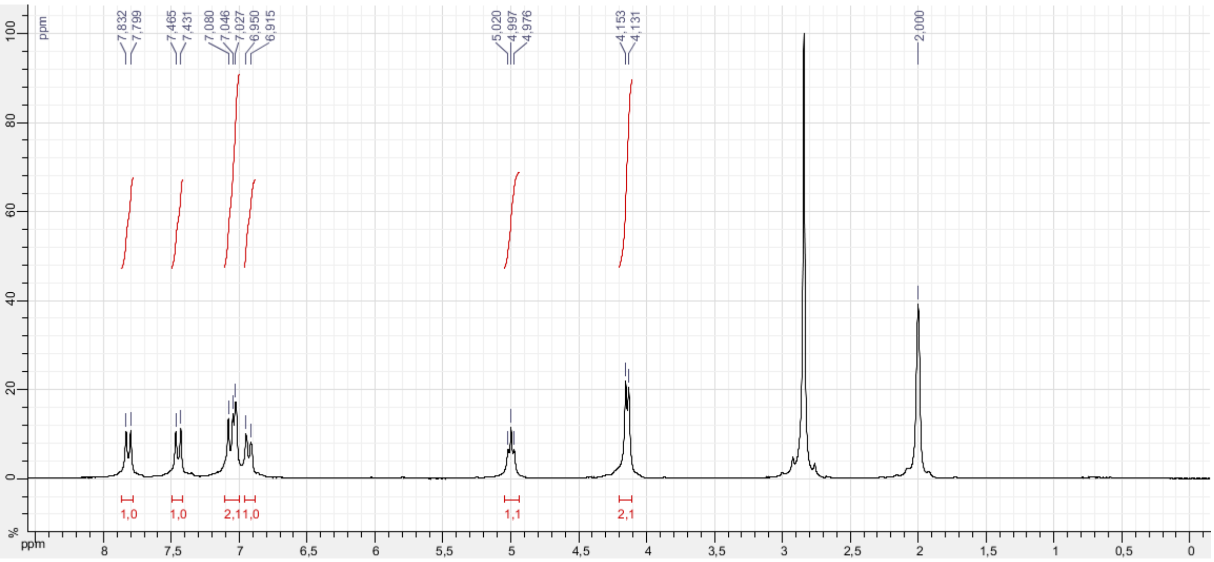

ppm
7,832
7,799
7,465
7,431
7,080
7,046
7,027
6,950
6,915
5,020
4,997
4,976
4,153
4,131
2,000
1,0
1,0
2,1
1,0
1,1
2,1
%

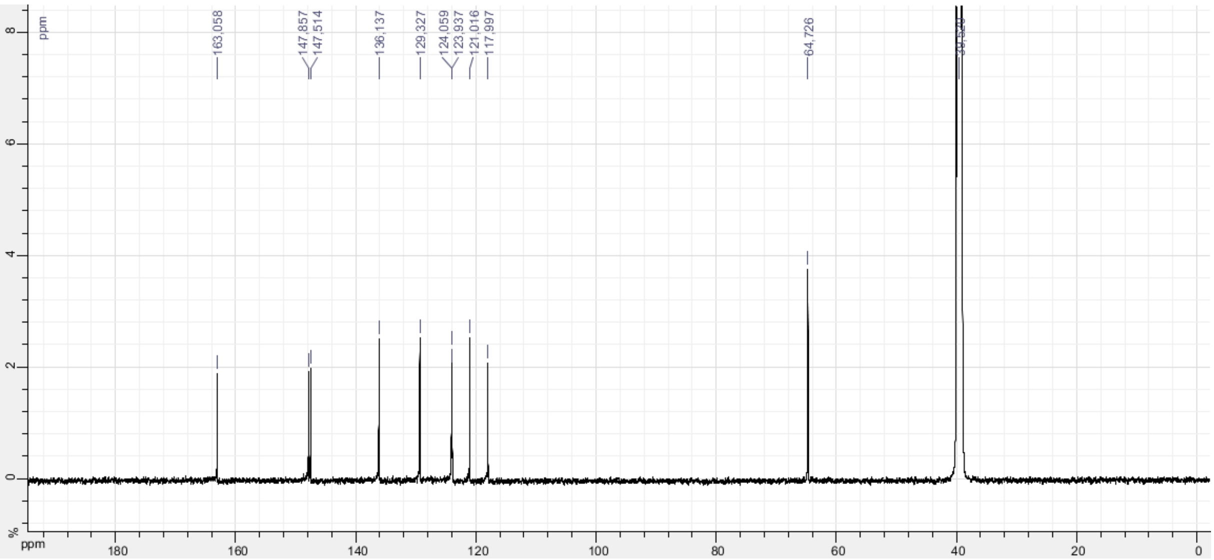

ppm
163,058
147,857
147,514
136,137
129,327
124,059
123,937
121,016
117,997
64,726
%

Compound 2b

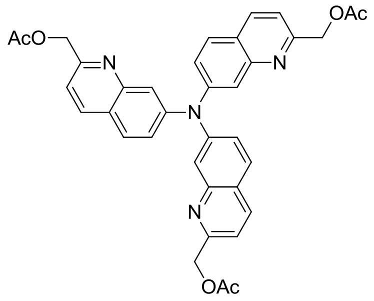
OAc
AcO
N
N
N
N
OAc

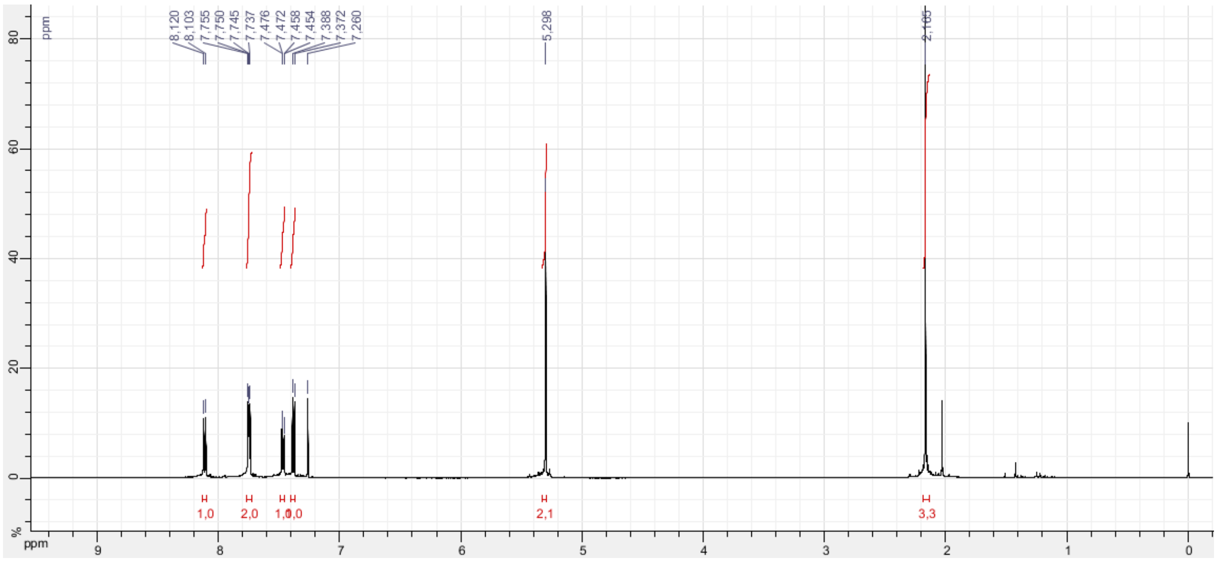
8,120
8,103
7,755
7,750
7,745
7,737
7,476
7,472
7,458
7,454
7,388
7,372
7,260
5,298
2,165
1,0
2,0
1,0
1,0
2,1
3,3
ppm
%

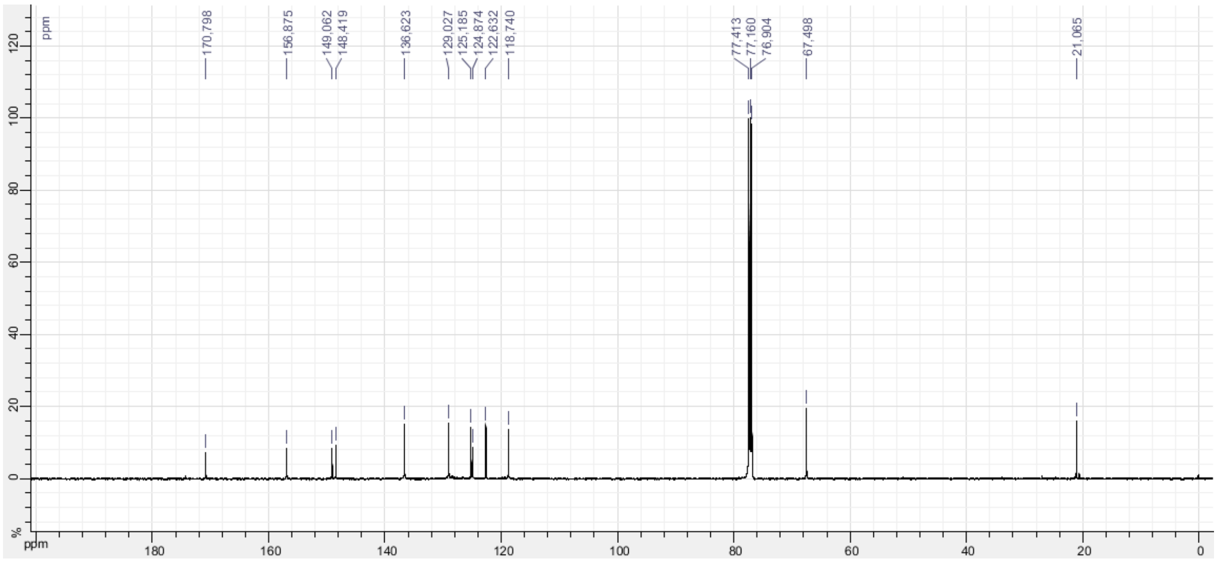
170,798
156,875
149,062
148,419
136,623
129,027
125,185
124,874
122,632
118,740
77,413
77,160
76,904
67,498
21,065
ppm
%

Compound 19a

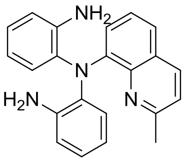

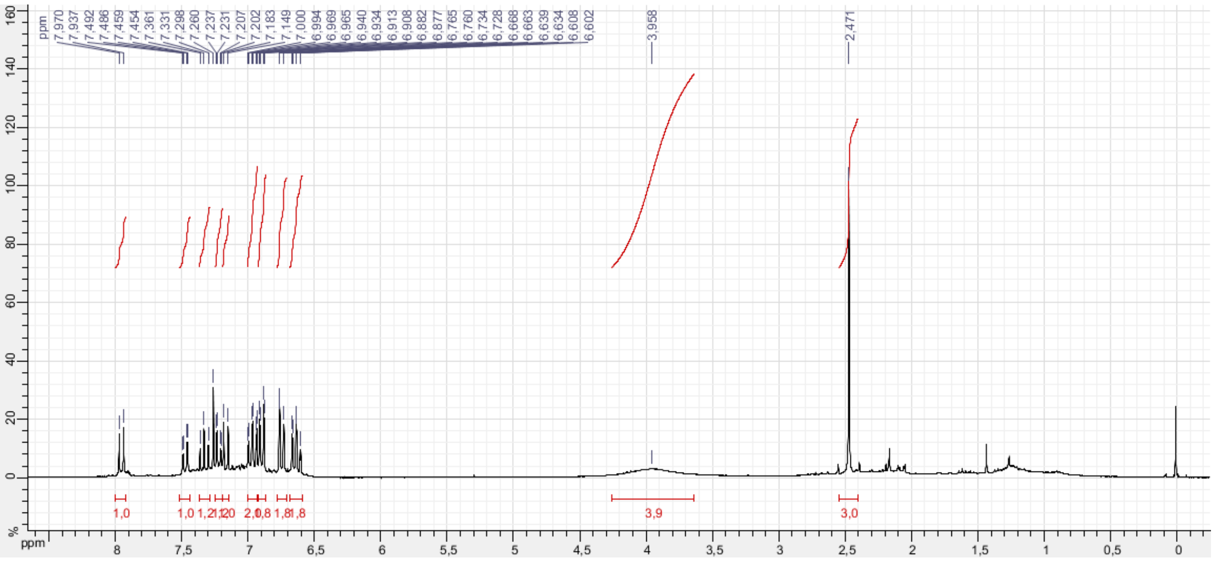

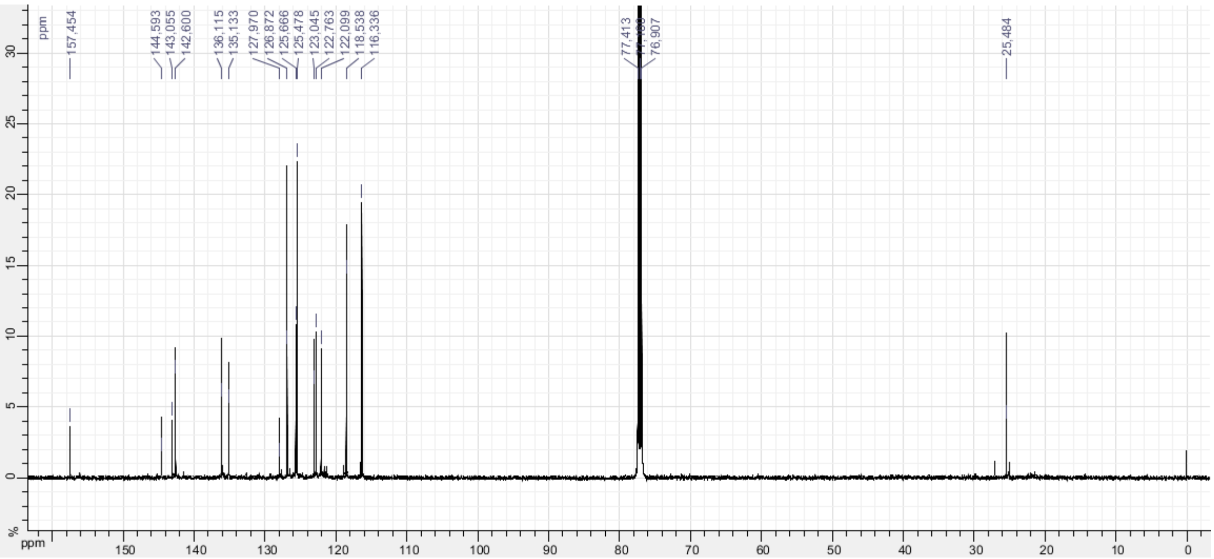

Compound 19b

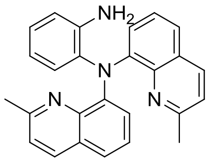

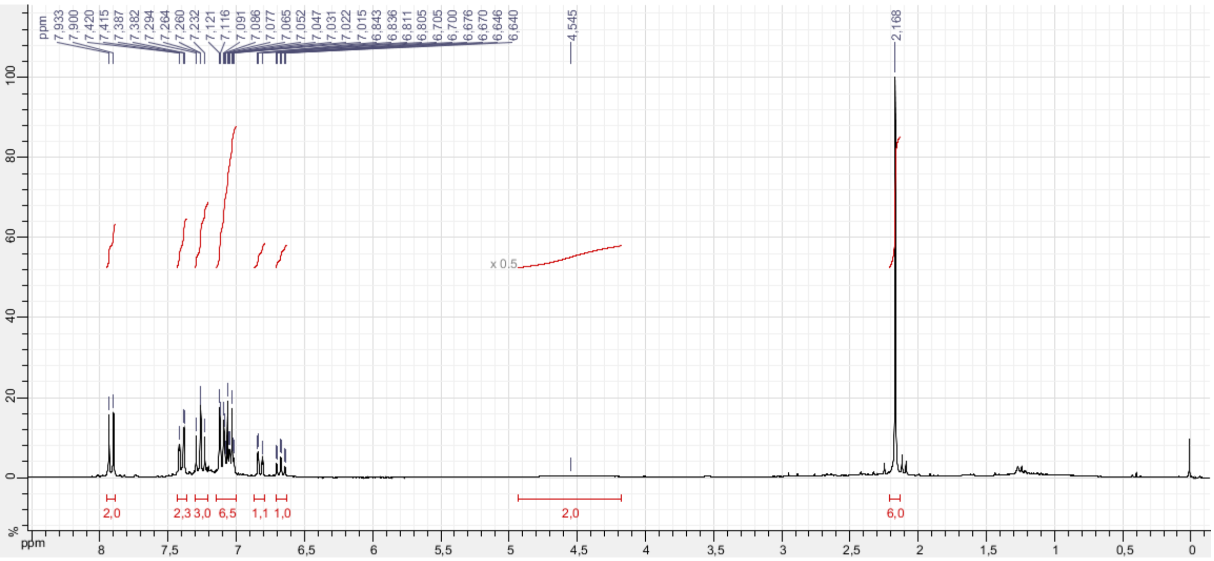

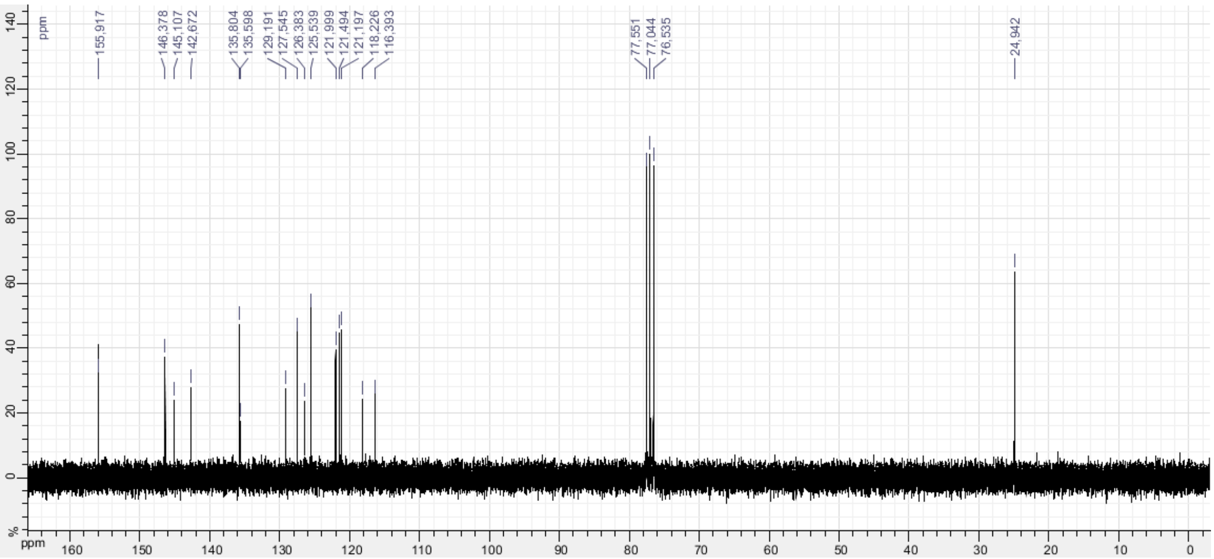

Compound 19c

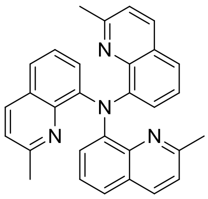

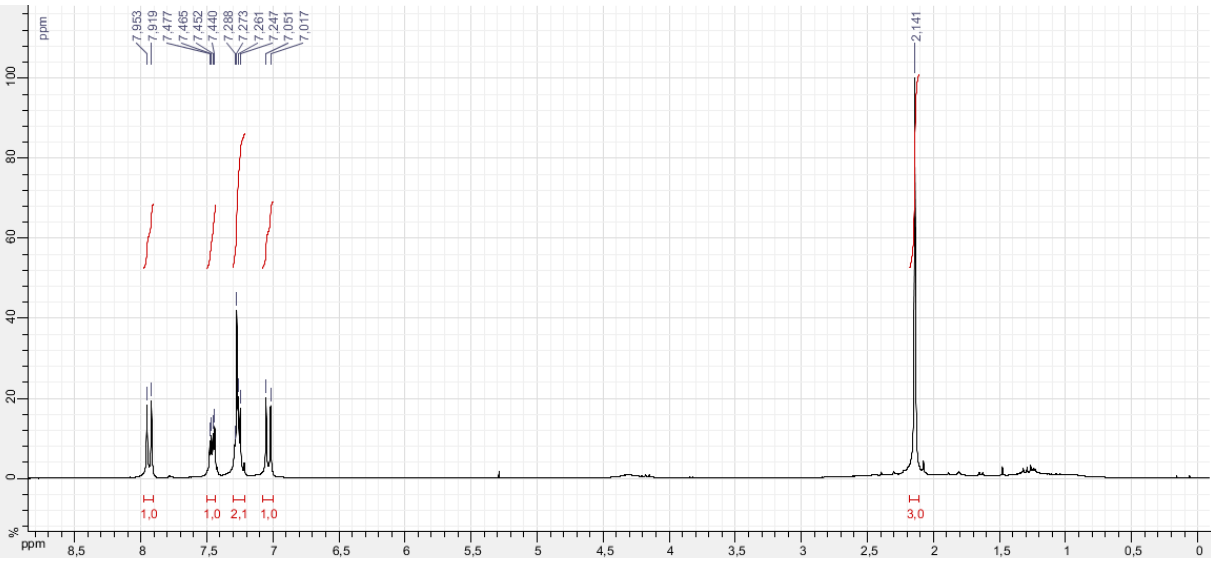

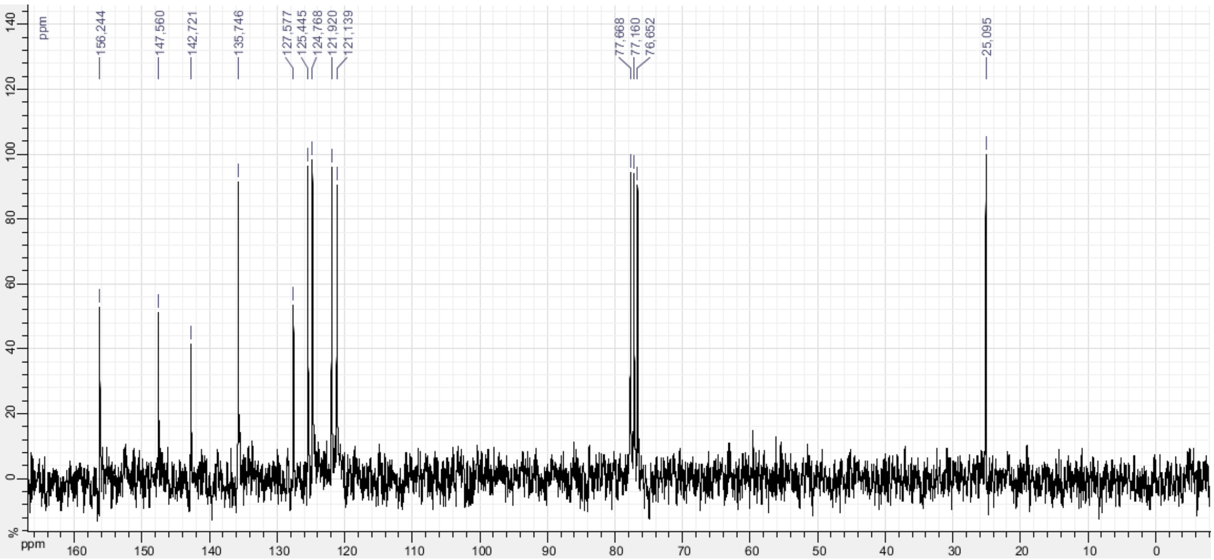

Compound 20

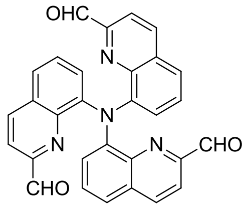
OHC
N
N
N
N
CHO
CHO

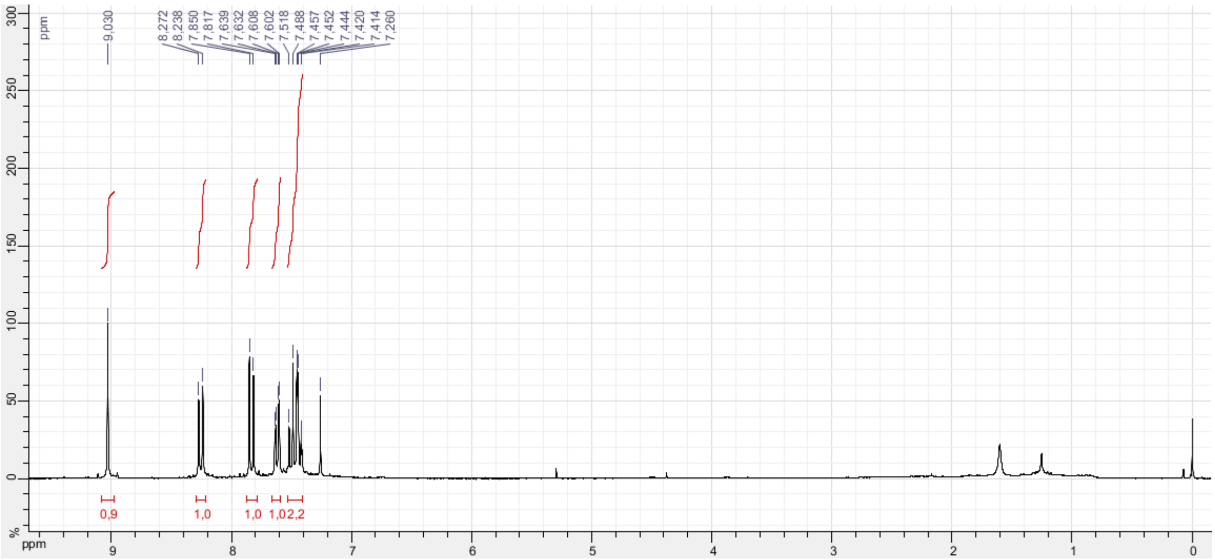
ppm
9,030
8,272
8,238
7,850
7,817
7,639
7,632
7,608
7,602
7,518
7,488
7,457
7,452
7,444
7,420
7,414
7,260
0,9
1,0
1,0
1,0
2,2
%
ppm
9
8
7
6
5
4
3
2
1
0
300
250
200
150
100
50
0

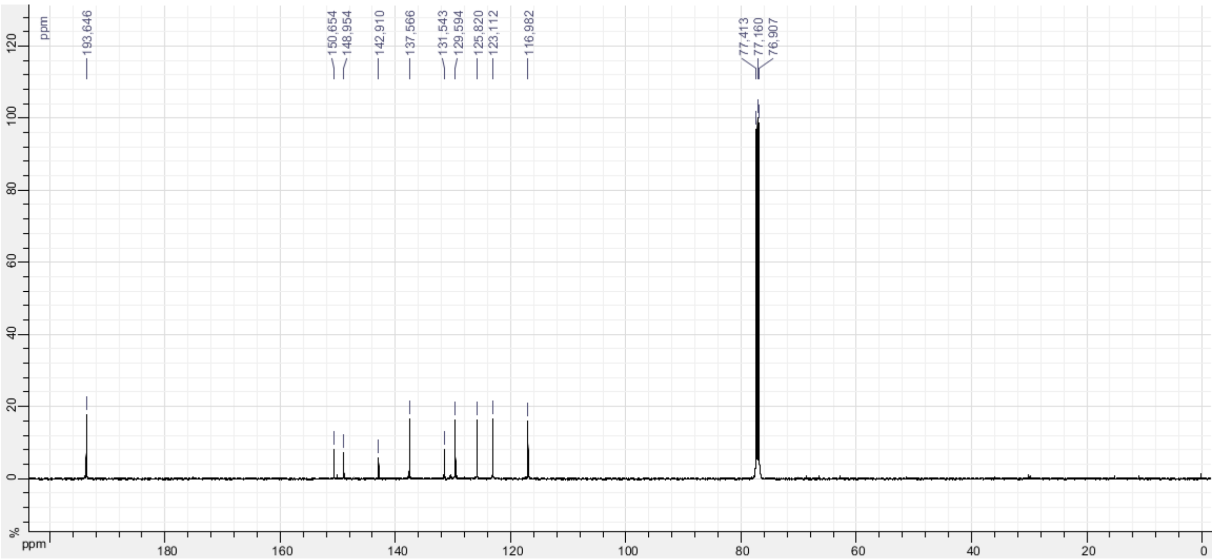
ppm
193,646
150,654
148,954
142,910
137,566
131,543
129,594
125,820
123,112
116,982
77,413
77,160
76,907
%
ppm
180
160
140
120
100
80
60
40
20
0
120
100
80
60
40
20
0

Compound 21

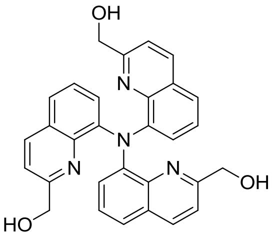
OH
N
N
N
N
OH
HO

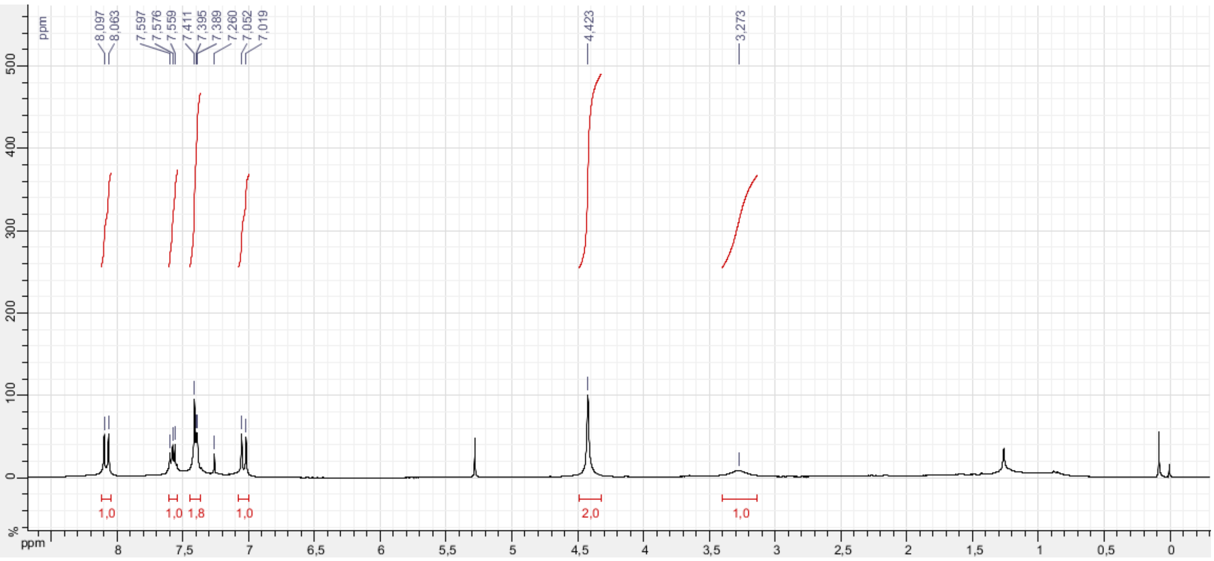
8,097
8,063
7,597
7,576
7,559
7,411
7,395
7,389
7,260
7,052
7,019
4,423
3,273
1,0
1,0
1,8
1,0
2,0
1,0
ppm

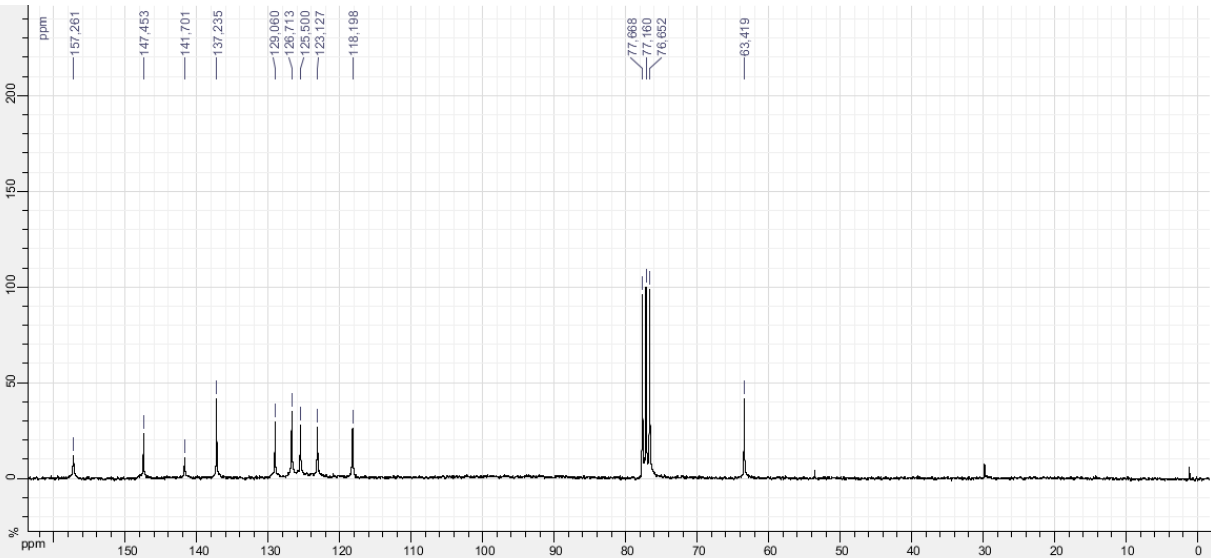
157,261
147,453
141,701
137,235
129,060
126,713
125,500
123,127
118,198
77,668
77,160
76,652
63,419
ppm

Compound 3b

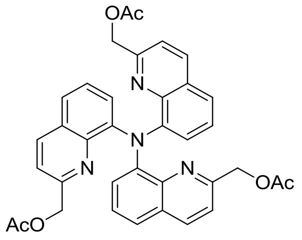
OAc
N
N
N
N
OAc
AcO

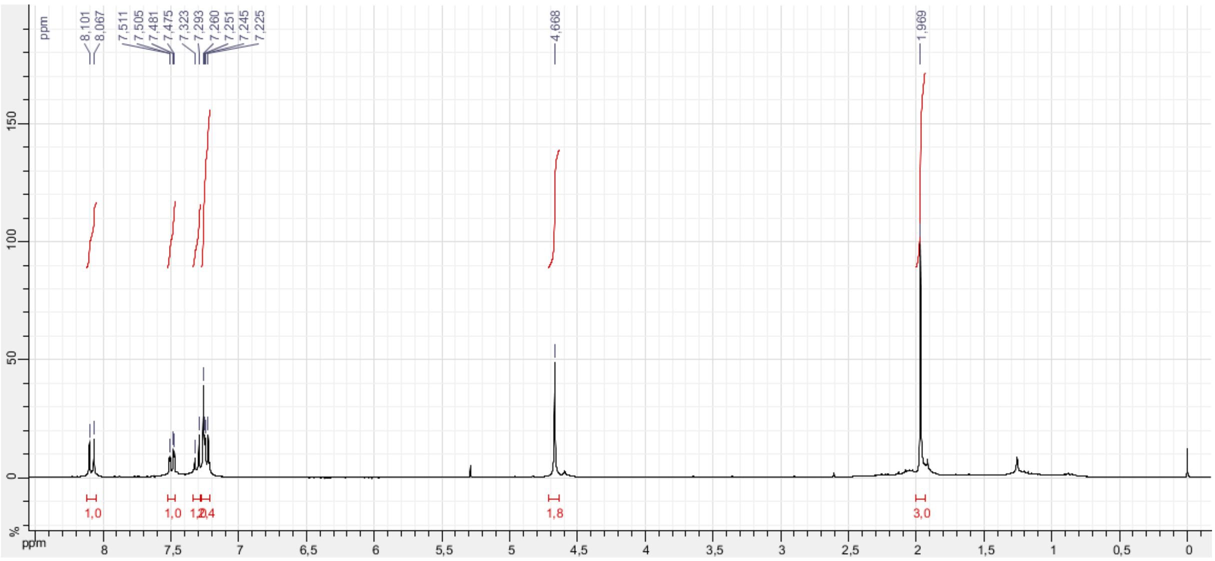
ppm
8,101
8,067
7,511
7,505
7,481
7,475
7,323
7,293
7,260
7,251
7,245
7,225
4,668
1,969
1,0
1,0
1,0
2,4
1,8
3,0
%
ppm

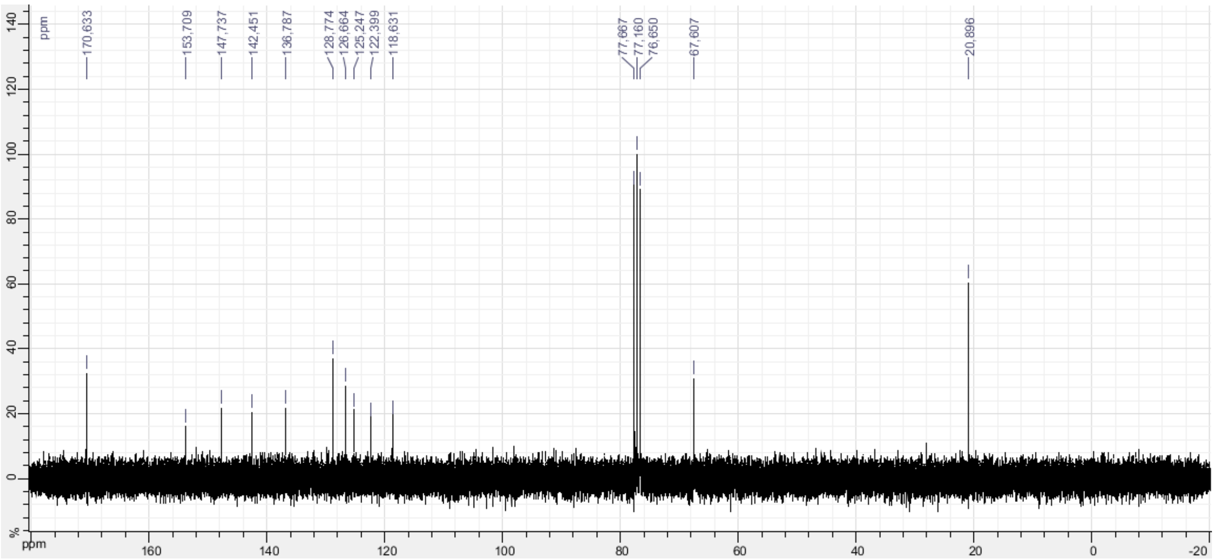
ppm
170,633
153,709
147,737
142,451
136,787
128,774
126,664
125,247
122,399
118,631
77,667
77,160
76,650
67,607
20,896
%
ppm
